# Coevolution of activating and inhibitory receptors within mammalian carcinoembryonic antigen families

**DOI:** 10.1186/1741-7007-8-12

**Published:** 2010-02-04

**Authors:** Robert Kammerer, Wolfgang Zimmermann

**Affiliations:** 1 Tumor Immunology Laboratory, LIFE Center, Klinikum Grosshadern, Ludwig-Maximilians-University, Marchioninistrasse 23, 81377 Munich, Germany; 2 Institute of Immunology, Friedrich-Loeffler-Institut, Paul-Ehrlich-Straße 28, D-72076 Tuebingen, Germany

## Abstract

**Background:**

Most rapidly evolving gene families are involved in immune responses and reproduction, two biological functions which have been assigned to the carcinoembryonic antigen (CEA) gene family. To gain insights into evolutionary forces shaping the CEA gene family we have analysed this gene family in 27 mammalian species including monotreme and marsupial lineages.

**Results:**

Phylogenetic analysis provided convincing evidence that the primordial CEA gene family in mammals consisted of five genes, including the immune inhibitory receptor-encoding *CEACAM1 *(CEA-related cell adhesion molecule) ancestor. Our analysis of the substitution rates within the nucleotide sequence which codes for the ligand binding domain of CEACAM1 indicates that the selection for diversification is, perhaps, a consequence of the exploitation of CEACAM1 by a variety of viral and bacterial pathogens as their cellular receptor. Depending on the extent of the amplification of an ancestral *CEACAM1*, the number of *CEACAM1*-related genes varies considerably between mammalian species from less than five in lagomorphs to more than 100 in bats. In most analysed species, ITAM (immunoreceptor tyrosine-based activation motifs) or ITAM-like motif-containing proteins exist which contain Ig-V-like, ligand binding domains closely related to that of CEACAM1. Human CEACAM3 is one such protein which can function as a CEACAM1 decoy receptor in granulocytes by mediating the uptake and destruction of specific bacterial pathogens via its ITAM-like motif. The close relationship between *CEACAM1 *and its ITAM-encoding relatives appears to be maintained by gene conversion and reciprocal recombination. Surprisingly, secreted CEACAMs resembling immunomodulatory CEACAM1-related trophoblast-specific pregnancy-specific glycoproteins (PSGs) found in humans and rodents evolved only in a limited set of mammals. The appearance of *PSG*-like genes correlates with invasive trophoblast growth in these species.

**Conclusions:**

These phylogenetic studies provide evidence that pathogen/host coevolution and a possible participation in fetal-maternal conflict processes led to a highly species-specific diversity of mammalian CEA gene families.

## Background

One of the major goals of evolutionary biology is to identify the genetic changes underlying phenotypic differences between organisms. Recent analyses of mammalian genomes revealed that the expansion and contraction of gene families contribute much more than expected to the genomic differences of mammals [1]. The shaping of gene families is a potent and safe mechanism which enables adaptation to environmental cues, since gene duplications provide new raw genetic material that natural selection can subsequently modify, without losing the function of the original gene. In addition, the repetitive sequences within gene families enhance the probability of recombination which can change the function of a gene dramatically within a short period of time through, for example, the exchange of cytoplasmic signalling motif-coding regions of transmembrane protein genes. Gene duplication, followed by such recombination and gene conversion events can lead to genes encoding pairs of proteins with very similar extracellular ligand interacting regions and opposite signalling cytoplasmic motifs, as recently described for the natural killer (NK) cell inhibitory receptor Ly49I. This inhibitory receptor is engaged by the murine cytomegalovirus-encoded major histocompatibility class I-related protein which is expressed on infected cells thereby suppressing a NK-mediated antiviral response. Subsequently, a paralog of Ly49I, Ly49H, has evolved which associates with an adaptor (DAP12) containing an immunoreceptor tyrosine-based activation motif (ITAM) in the cytoplasmic tail and, thereby, activates immune cells that encounter virus infected cells [2,3]. Such rapid modification of genes is of particular relevance for gene families that function in immune recognition and reproduction. Species-specific pathogens, as well as species-specific differences, in reproduction (for example, diverse types of placentation) may require fundamentally different properties of such gene families, even in otherwise closely related species.

The carcinoembryonic antigen (CEA) gene family may represent a paradigm for such gene families since it is highly divergent in humans, mice and dogs, and its members have functions in both immune regulation and reproduction [4,5]. The CEA family is a member of the immunoglobulin superfamily (IgSF), consists of the CEA-related cell adhesion molecule (CEACAM) and the pregnancy specific glycoprotein (PSG) subgroups. In humans and mice the CEA family comprises 23 and 31 members, respectively [5]. They are basically composed of a leader, one N-terminal immunoglobulin variable (IgV)-like domain (N domain), followed by a variable number of two different types of Ig constant (IgC)-like domains (named A and B) with a few notable exceptions which confuse the original nomenclature: for example, rodent PSGs contain 2-9 consecutive N domains and CEACAM16 one N- and one C-terminal N domain with one A and one B domain in between. Homotypic and heterotypic adhesion is the most prominent function of these extracellular domains [6]. Interestingly, several pathogens in humans and mice exploit these domains to adhere to and infect their target cells [7].

The physiological functions of CEA family members are very diverse. They are mediated by the different modes of membrane anchorage and the various types of signalling motifs within their cytoplasmic tails. Members of the CEA family can be secreted, glycosylphosphatidyl inositol (GPI)-anchored or transmembrane proteins with either a very short or a long cytoplasmic tail which can harbour immunoreceptor tyrosine-based inhibition motifs (ITIMs), immunoreceptor tyrosine-based switch motifs (ITSMs) or ITAMs. The GPI-anchored CEACAMs, which have been identified so far only within primate CEA families, were generated by mutational modification of a transmembrane domain exon in a *CEACAM1*-like ancestor [8]. The human GPI-linked CEACAM5/CEA and CEACAM6 are involved in the maintenance of the gastrointestinal tissue architecture and have been shown to contribute, when overexpressed, to tumour formation in the colon by the inhibition of differentiation and anoikis [9]. These functions are critically dependent on the presence of the GPI anchor [10].

*CEACAM1 *is the most widely expressed member of the CEA gene family, exhibiting extensive differential splicing which leads to multiple isoforms, some of which contain ITIM/ITSM. CEACAM1 was found in epithelial, endothelial and in a variety of immune cells including B cells, T cells, NK cells, dendritic cells, macrophages and granulocytes [11-14]. Due to its homophilic adhesion function, CEACAM1 seems to be one of the central receptors facilitating the communication of immune cells. Therefore, CEACAM1 seems to be an attractive receptor for pathogens to infect cells and simultaneously disrupt well coordinated immune responses. Indeed, it was previously shown that *neisserial *pathogens can use CEACAM1 to inhibit adaptive immune responses by ITIM signalling in CD4^+ ^T cells and, thereby, effectively suppressing B cell responses [15]. More recently, it was found that pathogens can also utilize CEACAM1 on bronchial epithelial cells to suppress TLR2 signalling, resulting in the downregulation of the innate immune response to bacterial pathogens [16]. Interestingly, CEA family members with N domains closely related to the corresponding CEACAM1 N domains, but with differently signalling cytoplasmic motifs, are found on granulocytes of humans (CEACAM3) and dogs (CEACAM28) [4,17]. CEACAM3 mediates the opsonin-independent recognition of a restricted set of human-specific Gram-negative bacterial pathogens, including *Neisseria gonorrhoeae, Haemophilus influenzae *and *Moraxella catarrhalis*, and supports their engulfment and elimination [18-20]. This property of CEACAM3 is critically dependant on the presence of the ITAM-related motif [17,19]. Since these bacteria use CEACAM1 as their cellular receptor, it is likely that the formation of such molecules with ITAM-like motifs is an adaptation to bacterial attacks. In mice, CEACAM1 is a cellular receptor for mouse hepatitis virus (MHV) [21]. Two distinct *CEACAM1 *alleles exist (*CEACAM1*^*a *^and *CEACAM1*^*b*^). Only CEACAM1^*a *^represents a high affinity receptor for MHV [22].

The trophoblast-specific PSGs are essential for a successful pregnancy [23]. They represent secreted proteins which are thought to regulate the maternal immune response to fetal trophoblast invasion which enables the fetus to access maternal resources [24]. They have been found in humans, mice and rats - species with haemochorial placentation [25,26]. Surprisingly, no *PSG *genes have been identified in dogs or cattle which have endotheliochorial and epitheliochorial placentae, respectively [4,27,28]. Analysis of *PSGs *in additional species and comparison with their type of placentation may strengthen the PSG/placentation type correlation.

In addition, more distantly related *CEACAMs *(*CEACAM16, CEACAM18, CEACAM19 and CEACAM20*) of unknown function exist for which orthologs could be assigned in rodents and primates [5]. The function of CEACAM/PSG family members as pathogens receptors, as well as their support of a successful outcome of pregnancy, suggests that pathogen-mediated and fetal-maternal conflict-induced selection are potential key drivers of CEA family evolution. Comprehensive analyses of mammalian *CEA *gene families may enable us to evaluate this hypothesis. Here we describe analyses of the *CEA *gene family in different mammals, including all five eutherian superclades, marsupial and monotreme clades. We discovered an unexpected diversity of the CEA family with respect to secreted PSG-like proteins between different clades, as well as within individual clades, and evidence for the coevolution of CEACAMs with ITIM and ITAM-like signalling motifs. Our results provide further evidence that the *CEA *gene family is shaped by pathogen-host interaction and species-specific reproductive requirements.

## Results

The few *CEA *gene families that have been studied in detail revealed a perplexing diversity, the reason for which is still unknown. In order to elucidate the forces which shaped the *CEA *gene families in mammals, we analysed all publicly available whole mammalian genomes with high (5-12X) as well as with low coverage (~2X) for the presence of *CEA *gene family members (Table 1). We used the sequences from known members of the *CEA *gene family of humans, mice and dogs to search for *CEACAM *genes in other species, assuming that the *CEACAM *genes of all mammals are composed of the principle exons found in these species [4,5]. Our exon predictions were validated by a comparison with EST information when available. With the possible exception of exons encoding cytoplasmic domains with ITAM in marsupials (see below), we did not find any new exons that had not been seen before in humans, mice, rats and dogs, indicating that the variability of the *CEA *gene families in eutherians is due to the different arrangement of these basic exons.

**Table 1:** Characteristics of CEACAM1-related genes of analyzed mammalian CEA families

abbreviation	Latin name	common name	clade	fold sequencing coverage	CEACAM1-related genes					
					
					total	pseudo-genes	GPI-liked members	soluble members	members with ITAM/ITAM-like motif	members with ITIM/ITSM
Bta	*Bos taurus*	cattle	laurasiatheria	7	6	1	0	1	0/2	0/1

Cfa	*Canis familiaris*	dog	laurasiatheria	7.6	9	2	0	0	0/4	0/1

Cpo	*Cavia porcellus*	Guinea pig	euarchontoglires	6.8	14	7	n.d.	n.d.	0/1	1/1

Dno	*Dasypus novemcinctus*	armadillo	xenarthra	2	10	0	n.d.	0	0/4	0/1

Eca	*Equus caballus*	horse	laurasiatheria	7	27	13	0	7	0/1	0/2

Eeu	*Erinaceus europaeus*	hedgehog	laurasiatheria	1.9	18	5	n.d.	n.d.	n.d	n.d

Ete	*Echinops telfairi*	lesser hedgehog tenrec	afrotheria	2	5	2	0	n.d.	0/1	n.d.

Fca	*Felis catus*	cat	laurasiatheria	1.9	3	0	n.d.	n.d.	0/1	0/1

Hsa	*Homo sapiens*	man	euarchontoglires	finished	29	11	4	10	0/2	1/0

Laf	*Loxodonto africana*	elephant	afrotheria	2	8	0	n.d.	n.d.	0/2	0/1

Mdo	*Monodelphis domestica*	opossum	marsupialia	7.3	21	6	0	5	4/0	1/1

Meu	*Macropus eugenii*	tammar wallaby	marsupialia	2	19	n.d.	n.d.	n.d.	4/0	0/0

Mlu	*Myotis lucifugus*	small brown bat	laurasiatheria	1.7	>100	n.d.	n.d.	n.d.	0/1	1^b^

Mml	*Macaca mulatta*	rhesus macaque	euarchontoglires	5.2	28	5	4	13	0/2	1/0

Mmu	*Mus musculus*	mouse	euarchontoglires	finished	28	1	0	24	0/0	0/2^a^

Mmr	*Microcebus murinus*	gray mouse lemur	euarchontoglires	2	7	2	n.d.	n.d.	0/1	0/1

Oan	*Ornithorhynchus anatinus*	platypus	monotremata	6	5	0	n.d.	n.d.	n.d.	1/0

Ocu	*Oryctolagus cuniculus*	rabbit	euarchontoglires	2	1	0	0	0	0/0	0/1

Oga	*Otolemur garnettii*	bush baby	euarchontoglires	1.5	3	1	1	0	n.d.	0/1

Opr	*Ochotona princeps*	American pika	euarchontoglires	1.9	2	1	n.d.	n.d.	n.d.	1^b^

Ppy	*Pongo pygmaeus*	orangutan	euarchontoglires	7	28	11	4	9	0/2	1/0

Ptr	*Pan troglodytes*	chimpanzee	euarchontoglires	6	27	11	4	9	0/2	1/0

Rno	*Rattus norvegicus*	rat	euarchontoglires	11.9	18	0	0	14	0/0	0/1^a^

Sar	*Sorex araneus*	European shrew	laurasiatheria	1.9	3	0	n.d.	n.d.	0/1	1^b^

Str	*Spermophilus tridecemlineatus*	ground squirrel	euarchontoglires	1.9	4	1	n.d.	n.d.	0/1	0/1

Ssc	*Sus scrofa*	pig	laurasiatheria	4	3	0	1	0	0/1	0/1

Tbe	*Tupaia belangeri*	tree shrew	euarchontoglires	2	5	1	n.d	n.d	0/1	0/1

CEACAM gene identification in species with unfinished genome sequences are based partly on N domain exon counts. Genes were registered as pseudogenes when stop codons or frame shift mutations were detected in N domain exons. The number of members with ITAM or ITAM-like motifs and ITIM or ITSM are separated by slashes. For low coverage genome sequences gene numbers represent a minimal estimate.

^a ^allelic variants exist which were not taken into account; ^*b *^neither classifiable as ITIM nor as ITSM (e.g. PxYxxI); n.d., not determined.

### The origin of orthologous *CEA *gene family members in mammals

**Figure 1:**
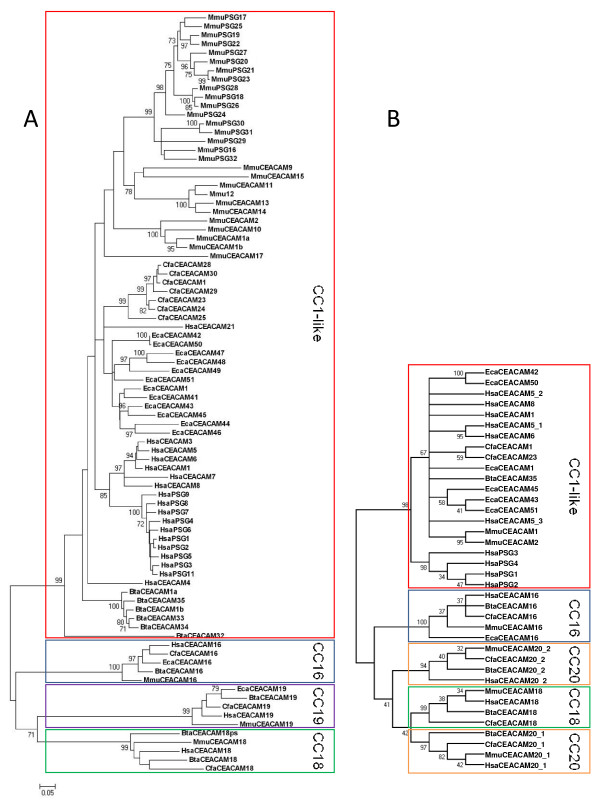
**Phylogeny of mammalian carcinoembryonic antigen related cell adhesion molecule (*CEACAM) *genes**. Phylogenetic trees were constructed from N domain exon (A) and B domain exon nucleotide sequences (B) from *CEACAM *(CC) of cattle, horses, dogs, humans and mice using the neighbour-joining method (MEGA 4.0 software). The reliability of a phylogenetic tree was assessed using the Bootstrap test applying 1000 replicates. The statistical support for selected nodes is shown. Values >70 and >30 are shown in (A) and (B), respectively. Due to space limitations, only selected murine and horse *Ceacams/CEACAMs *and *Psg *were included and the topology of the B domain phylogenetic tree is shown (B). N1 domain exons were used from murine *Psg *genes which contain multiple N domain exons. Five groups of N domain exons can be discriminated. The N domain exon sequences from the *CEACAM16, CEACAM18, CEACAM19 *and *CEACAM20 *genes each cluster together, while N exon sequences from *CEACAM1*-like genes including *PSG *genes form species-specific groups. Similar groups were discriminated using B domain exons (B). *CEACAM1 *genes were identified based on the presence of immunoreceptor tyrosine-based inhibition motifs (ITIM)/immunoreceptor tyrosine-based switch motif (ITSM) in the encoded cytoplasmic domains. In horse, two genes with intact ITIM/ITSM were identified and named arbitrarily *CEACAM1 *and *CEACAM43*. For abbreviation of species names see Table 1. The bar below the phylogenetic tree in (A) shows the scale for the number of substitutions per site. ^a, b^, allelic *CEACAM1 *variants. Multiple B domains present in a CEACAM are indicated by numbers.

**Figure 2:**
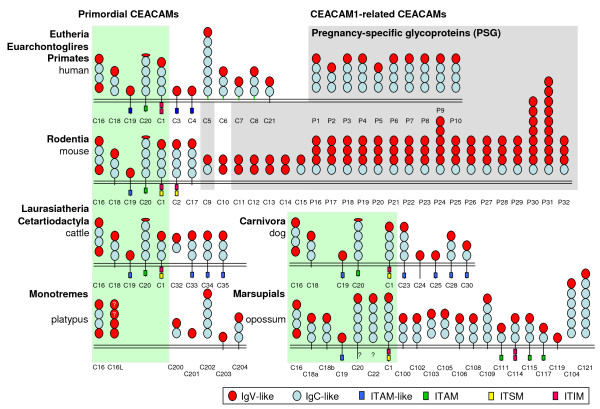
**Domain organization of mammalian carcinoembryonic antigen (CEA) family members**. The domain organization of CEA family members from selected species was predicted by gene analysis and confirmed, where available, by EST (cattle) and cDNA sequences [4,5,27,35]. The conserved members are shown in green, members expressed predominantly in trophoblast cells of the placenta in grey boxes. The predicted signaling motifs in the cytoplasmic domains are schematically shown as green (immunoreceptor tyrosine-based activation motifs; ITAM), blue (ITAM-like motif, no acidic amino acid present at position -1 to -3 from first Y in consensus motif E/Dx_0-2_YxxL/Ix_6-8_YxxL/I), red (immunoreceptor tyrosine-based inhibition motifs) and yellow boxes (immunoreceptor tyrosine-based switch motif). Due to gaps in the genomic sequences, some domains could not be clearly identified for a few proteins which are indicated by a question mark. C, CEA-related cell adhesion molecule.

**Figure 3:**
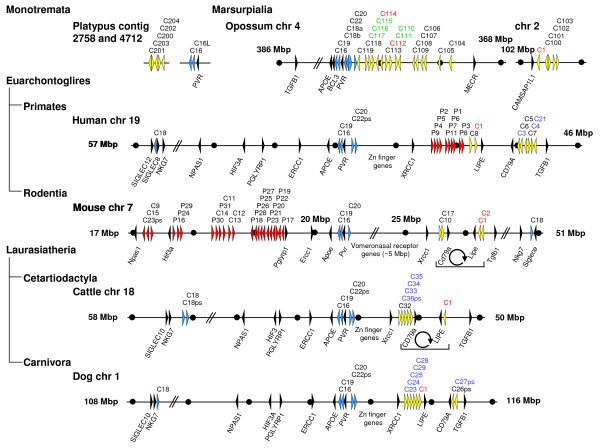
**Syntenic relationship of the carcinoembryonic antigen related cell adhesion molecule *(CEACAM) *gene loci in platypus, opossum, human, mouse, cattle and dog genomes**. The syntenic chromosome regions from six mammalian species are shown except for platypus where only contig information is available. Arrowheads represent genes with their transcriptional orientation. *CEACAM1*-related genes are indicated in yellow or red when predominantly expressed in trophoblast cells, the orthologous *CEACAM *genes in blue and marker genes in black. Names of *CEACAM1*-like genes with immunoreceptor tyrosine-based inhibition motif/immunoreceptor tyrosine-based switch motif encoding exons are shown in red and with immunoreceptor tyrosine-based activation motif (ITAM) and ITAM-like motif-encoding exons in green and blue, respectively. Clockwise oriented arrows symbolize inversion events of the regions between the two *CEACAM1*-like gene clusters which have taken place independently in two different clades. Note that, in general, the genes in the same subcluster show the same transcriptional orientation, between the two subclusters opposite transcriptional orientation. The ancestral gene arrangement is unknown. Therefore, indication of species with inversion events is arbitrary; inversions relative to the human gene order are shown. The nucleotide numbering of the chromosomes starts at the telomere of the short arms. The scale indicated by dots is 1 Mbp unless interrupted by slanted lines. Note the inverse orientation of the opossum chromosome 4, cattle chromosome 18 and human chromosome 19 regions. C, *CEACAM/Ceacam*; chr, chromosome; P, *PSG/Psg*.

In order to determine the relationship of *CEA *family genes in mammals we constructed a phylogenetic tree using N domain exon nucleotide sequences from representative *CEACAMs *of selected species with almost completely sequenced genomes. While N exon nucleotide sequences of *CEACAM16, CEACAM18, CEACAM19 *and *CEACAM20 *from different species cluster together, all other *CEACAM *N domain exon sequences of a given species are most closely related with the corresponding *CEACAM1 *sequence of that species, indicating that these genes were generated independently by multiple rounds of duplication of an ancestral *CEACAM1 *gene possibly followed by gene conversion events (Figure 1A). This notion is supported by similar analyses using IgC-like A and B domain exons. Results are shown to be exemplarily for the latter (Figure 1B). Again, *CEACAM1*-like B domain exons are clearly separated from that of *CEACAM16, CEACAM18 *and *CEACAM20 *which form their own clusters with representatives from each species. However, no species-specific clustering is observed for *CEACAM1*-like B domain exons as is seen for the N domain exons. This could be due to the lack of strong selection pressure to conserve sequences such as those found for the N domain exons (see below). In all high coverage species one or two *CEACAM1 *genes can be identified by the presence of ITSM and/or ITIM in the cytoplasmic domain encoded by a conserved set of three exons. We consider, therefore, the ancestors of the conserved (orthologous) members of the *CEA *gene family, namely *CEACAM1, CEACAM16, CEACAM18, CEACAM19 *and *CEACAM20 *to be the primordial *CEA *gene family. Since they are found in all therian species (marsupials and placental mammals) with high coverage genomic sequencing, ancestral versions of these conserved genes must have been present in the most recent common ancestor of these mammals. In the primordial *CEA *gene family, except for *CEACAM1*-related genes, only very rarely have gene duplications/deletions taken place. A second *CEACAM16*-related gene exists in the platypus (*CEACAM16L*, L stands for like) which consists of four N domain exons (Figure 2). Furthermore, in the short-tailed opossum *CEACAM18 *was duplicated (*CEACAM18a, CEACAM18b*). In cattle, one of the duplicated *CEACAM18 *genes most probably represents a pseudogene (*CEACAM18ps*; Figures 2 and 3). A second *CEACAM20*-like gene (*CEACAM22*) with identical extracellular domain composition (LNA1B1A2B2) exists in the opossum. Remnants of *CEACAM22 *can still be found in the human, cow and dog genomes at a syntenic location (Figure 3). So far, only *CEACAM16 *from the *CEACAM16-CEACAM20 *gene cluster has been identified in the monotreme platypus despite completion of its genome sequence (6X coverage; Figure 3).

### Reconstruction of the ancestral *CEA *gene locus

In order to learn more about the evolution of the *CEA *gene locus we determined the chromosomal arrangement of the *CEA *gene family in distantly related mammalian species with high coverage genome sequencing or finished genomes. In the opossum, except for *CEACAM1*, all genes with identifiable orthologues form a cluster (*CEACAM16, CEACAM19, CEACAM18a, CEACAM18b, CEACAM22, CEACAM20*) only interrupted by one non-*CEA*-related gene (*polio virus receptor*) directly upstream of *CEACAM19 *(Figure 3). Directly downstream of *CEACAM16 *the *BCL3 *is found. This syntenic neighbourhood could be demonstrated for all analysed mammalian species. Analysis of the *CEA *gene loci of selected placental mammals (humans, mice, cattle, dogs) revealed that translocation of *CEACAM18 *from the *CEACAM16-CEACAM20 *gene cluster to the *SIGLEC *gene cluster must have occurred in the common ancestor of placental mammals after radiation from marsupials (Figure 3). Interestingly, in the opossum the conserved members of the *CEA *family are directly located next to an expanded cluster of 16 *CEACAM1*-related genes. After the separation of placental mammals from marsupials, the insertion of Zn finger genes in the eutherian lineage followed by insertion of vomeronasal receptor genes in rodents separated the orthologous *CEACAM *genes from the *CEACAM1*-related genes. The latter genes were further split into two loci by the insertion of a chromosomal region flanked by *LIPE *and *CD79A *(Figure 3). Surprisingly, compared to humans and dogs, inversions must have taken place independently within different clades in cattle and mice between the two *CEACAM1*-like gene loci. This is evident from the inverted order of the intervening genes bracketed by *LIPE *and *CD79A *(Figure 3). Furthermore, the expansion of PSG-like genes took place either close to *CEACAM1*, as in higher primates, or distal to the orthologous *CEACAM *gene cluster, as found in rodents (Figure 3).

### Delineation of functionally important domains and sequence motifs

**Figure 4:**
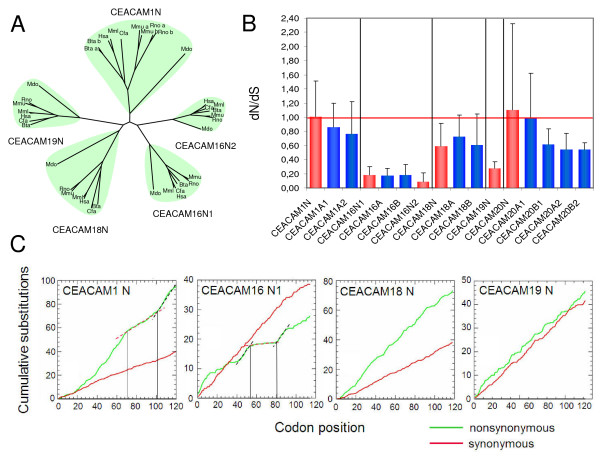
**Identification of functionally important extracellular regions of carcinoembryonic antigen related cell adhesion molecules (CEACAMs) by multispecies sequence comparisons**. N domain exon nucleotide sequences from orthologous *CEACAMs *of a selected set of mammalian species were aligned using the program ClustalW and the results were displayed as unrooted dendrogram (A). Note the high and low conservation of the *CEACAM16 *N2 and *CEACAM1 *N nucleotide sequences, respectively, as reflected by the size of the colored area. In order to determine the selective pressure on the maintenance of the nucleotide sequences, the number of nonsynonymous nucleotide substitution per nonsynonymous site (dN) and the number of synonymous substitutions per synonymous site (dS; B) as well as the accumulation of nonsynonymous and synonymous substitutions along immunoglobulin (Ig) variable- and constant-like domain exons (C) were determined after manual removal of sequence gaps for the following species: cattle, dog, human, mouse, opossum, rat and rhesus macaque (due to missing sequence information for the rhesus macaque *CEACAM1 *A2 exon, the corresponding exon from cat was used instead). The dN/dS ratios were calculated after manual editing of sequence gaps or insertions guided by the amino acid sequences for all branches of the resulting phylogenetic trees and displayed as mean values and standard deviations. Whole N domain exons were used for analysis including the regions (the first 12 codons) which encode part of the leader. Due to the variable truncation of the CEACAM20 N domain exons, the first common 27 codons were analysed. The mean dN/dS ratios were calculated and displayed as red (N domain exons) or blue columns (Ig constant-like domain exons); standard deviations are shown as bars. dN/dS values significantly greater than 1 (above the red line) indicate selective pressure for variability, a ratio less than 1 indicates pressures to conserve the protein sequence. Regions with high and low accumulation rates of nonsynonymous substitutions have been marked exemplarily in some graphs by black and red dotted lines. Note that the synonymous substitutions accumulate at the same rate for all exons.

Since the extracellular N domains of CEACAMs have been demonstrated to be most instrumental for homo- and heterotypic adhesion, as well as for the interaction with pathogens, we performed phylogenetic analyses with the N domain nucleotide sequences of all orthologous *CEACAM *members from different species. The degree of nucleotide sequence conservation of the N domain exons differs greatly between the orthologous *CEACAMs *(Figure 4A). The *CEACAM16 *N2 domain exon is the most conserved N domain exon and the *CEACAM1 *N domain exon the least conserved. The conservation of the N domain exon sequences of *CEACAM18 *and *CEACAM19 *lies in between that of *CEACAM16 *and *CEACAM1 *(Figure 4A). Determination of ratios of nonsynonymous mutation rates and synonymous mutation rates (dN/dS) to normalize for variable evolutionary distances revealed that the exon sequences from the various *CEACAMs *are under different selective pressure (Figure 4B). Highest selective pressure is exerted on the *CEACAM16 *N2 exons (low dN/dS ratio) while *CEACAM1 *N domain exons do not appear to be under selective constrain (dN/dS ratio ≅ 1). Interestingly, in the *CEACAM20 *gene only B1, A2 and B2 exons exhibit some selective pressure (dN/dS ratio < 1). Based on the accumulation of nonsynonymous substitutions along codons for the extracellular domains of seven species from five different mammalian orders, regions with high and low pressure for conservation marked by red and black dotted lines, respectively, could be identified (Figure 4C: note the extended conserved regions (>20 codons) in the *CEACAM1 *N and *CEACAM16 *N1 domain exons). In contrast, only short stretches of higher sequence conservation can be seen in the *CEACAM19 *N domain exon. In comparison, the synonymous substitutions accumulate at the same rate (34.5 +/- 1.6 substitutions per 100 codons) for all N domain exons (Figure 4C).

In the N domain amino acid sequence of CEACAM1 from 13 species, including mice, rats and cattle CEACAM1 allelic variants, only seven positions are conserved. Most of these positions appear to be critical for the formation of the typical β-strands and β-sheets in IgV-like domains since they are also conserved in other CEACAM (Additional File 1) and IgSF members [29]. Among the conserved amino acids is an arginine which forms in most IgV-like N domains of the CEA family, a salt bridge with an aspartic acid [30] and an asparagine which is part of a highly conserved N-glycosylation consensus sequence. Most amino acid positions, which are important for interactions between CEACAMs and their viral and bacterial ligands, are located in the CFG face where no conservation is observed at all (Additional File 1). Together with the overall dN/dS ratio ≈ 1 observed for the *CEACAM1 *N domain exon this indicates selection for divergence in the regions dispensable for maintenance of the basic Ig structure.

**Figure 5:**
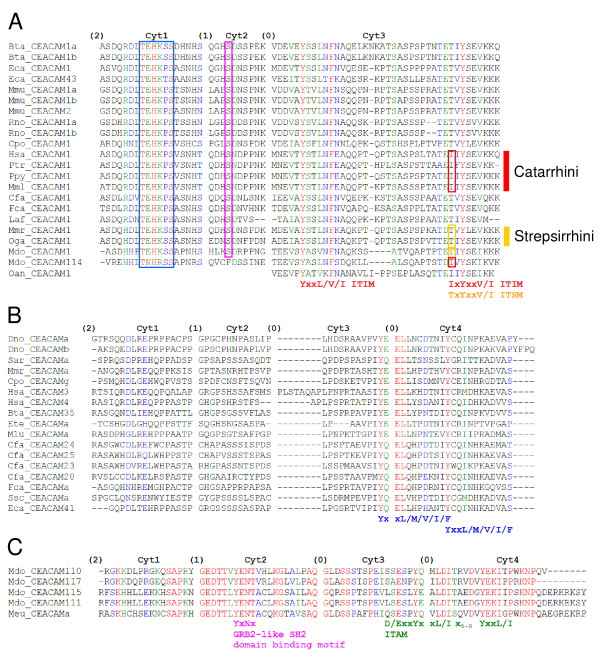
**Identification of functionally important intracellular sequence motifs of carcinoembryonic antigen related cell adhesion molecule (CEACAM1) and CEACAM1-related proteins by multispecies sequence comparisons**. The amino acid sequences encoded by cytoplasmic domain exons of *CEACAM1 *(A), CEACAM1-like members containing an immunoreceptor tyrosine-based activation motif (ITAM)-like motif from eutherians (B) and marsupials (C) from the indicated species were aligned. The sequences were separated according to exon borders. The names of the cytoplasmic domain encoding exons (Cyt) and the intron types (0, xxx-intron-xxx; 1, x-intron-xx; 2, xx-intron-x; xxx = codon) are indicated. The following potential motifs could be detected where x represents any amino acid, and slashes separate alternative amino acids (in one letter code) that may occupy a given position and are indicated below the corresponding sequences: an immunoreceptor tyrosine-based inhibition motif (ITIM) is defined by the sequence (I/L/V/S)xYxx(L/V), an immunoreceptor tyrosine-based switch motif (ITSM) by TxYxx(V/I), an ITAM by (D/E)xxYxxX(L/I)x_6-8_Yxx(L/I) and an endocytic, ITAM-like motif by Yxx(L/M/V/I/F). In catarrhinian primates and in platypus the ITSM has been switched to an ITIM. The opossum has two CEACAM1-like proteins one with two ITIM, and one with an ITIM and an ITSM. Note the characteristic split of the YxxL motif in the ITAM and ITAM-like motifs by phase 0 introns. The opossum ITAM domains could be predicted using an EST sequence [GenBank: EX196902] from the marsupial *Macropus eugenii *(tammar wallaby). An additional highly conserved motif (TEHKxS) and a conserved serine in the cytoplasmic domain of CEACAM1 proteins are boxed. For abbreviation of species names see Table 1.

Alignment of the cytoplasmic amino acid sequences of CEACAM1 from 16 different species revealed that only a few positions have been conserved between mammals. In the region encoded by cytoplasmic domain exon 3 of most species one ITIM and one ITSM each are found (Figure 5A). Catarrhinian apes, including humans, contain a second ITIM instead of the ITSM while, in more distantly related primates (strepsirrhini), the ITSM is still present. Two ITIM are also present in platypus and two copies of CEACAM1, one containing ITIM/ITSM, and the other two ITIM were found in the opossum. A second prominent motif (TEHKxS) is encoded by the cytoplasmic domain exon 1. This motif is also conserved in one of the two potential CEACAM1 proteins of opossum (Figure 5A). Finally, a serine, encoded by the second cytoplasmic exon of *CEACAM1 *is highly conserved and might be a target of serine kinases.

Many of the non-CEACAM1 transmembrane-bound members of the CECAM1-related group of proteins contain a cytoplasmic domain encoded by four exons mostly conserved in size. The sequences of nine species from four different mammalian clades were analysed and found to be highly variable except for a conserved ITAM-like motif which, however, lacks the negatively charged amino acid (D or E) N-terminal to the first tyrosine of canonical ITAM (D/ExxYxxL/Ix_6-8_YxxL/I; Figure 5B). Interestingly, no such members are found in mice and rats. In marsupials, CEACAM1-related members with canonical ITAM in their cytoplasmic domains and, unlike those in eutherian mammals, with a growth factor receptor-bound protein 2 (GRB2)-like SH2 domain binding motif (YxN) encoded by the cytoplasmic domain exon 2 could be identified. These domains are also encoded by four exons separated by same type introns (Figure 5C).

### The expansion of *CEACAM1*-related genes is species-specific and highly variable in mammals

As shown above, species-specific differences within the *CEA *gene families are mainly observed within the *CEACAM1*-related subgroup (Figure 1). We determined the size of the CEACAM1-related groups and the domain organization of their members in selected species of all four clades of placental mammals (euarchontoglires, laurasiatheria, afrotheria, xenarthra), as well as one member each from the marsupial infraclass and the monotreme order. In most species only a single gene encoding the CEACAM1 typical ITIM/ITIM or ITIM/ITSM signalling motifs was found. In a few species, such as mice (*CEACAM1, CEACAM2*), horses (*CEACAM1, CEACAM43*), bats (*CEACAM1, CEACAMa*) and opossum (*CEACAM1, CEACAM114*), two such genes were detected. In humans, the *CEACAM1*-related members of the *CEA *gene family including *CEACAM1 *consist of 18 genes and 11 pseudogenes (Table 1). Similar numbers of genes were found in other great apes (orangutans, chimpanzee) and in the rhesus macaques (*Macaca mulatta*). In more distantly related primates (*Otolemur garnettii, Microcebus murinus, Tupaia belangeri*) with low shotgun sequencing coverage (1.5-2X) only four to six *CEACAM1*-related genes could be detected. In mice, also members of the euarchontoglires clade, 27 *CEACAM1*-related genes and one pseudogene had been previously identified [5]. In contrast, the canine *CEA *gene family contains only nine *CEACAM1*-related genes, two of which seem to represent pseudogenes [4]. In cattle, the *CEACAM1 *subgroup of the *CEA *gene family is extremely small consisting of only six *CEACAM1*-related genes. These findings indicate that members of the laurasiatherian clade have contracted or less expanded *CEA *gene families. However, the genome of the small brown bat (*Myotis lucifugus*), another member of this clade, contains about 100 *CEACAM1*-related genes, most of which are apparently coding for secreted proteins (see below). The *CEA *gene families of two members of the afrotherian clade have also been studied. The African elephant (*Loxodonta africana*) and the Lesser Hedgehog Tenrec (*Echinops telfairi*) contain just eight and four *CEACAM1*-related genes, respectively. For both species, however, only low coverage genome sequence data are available (2X), which might have led to an underestimation of the size of the *CEA *gene families. In the armadillo (*Dasypus novemcinctus*), the only member of the fourth clade of placental mammals (xenarthra), we identified 10 putative *CEACAM1*-related genes. In the genome of the opossum (*Monodelphis domestica*), a representative of the marsupial lineage, we could identify 21 *CEACAM1*-related genes located on two chromosomes (chromosome 2 and 4). Monotremata represent the most divergent mammals and exhibit both reptilian and mammalian features. Analyses of the genome sequence in the platypus, the only member of this group for which sequence data are publicly available, revealed the presence of at least five *CEACAM1*-related genes (Figure 3; Table 1).

**Figure 6:**
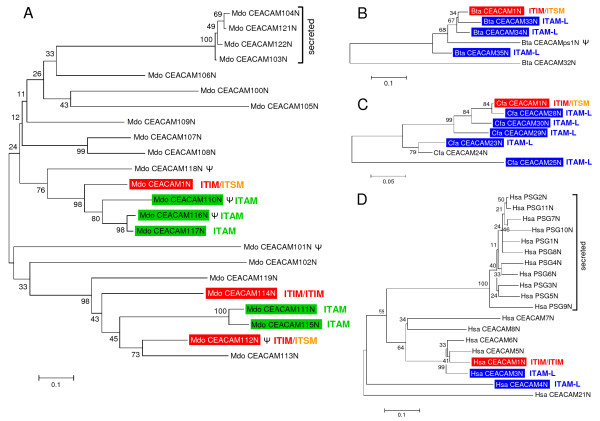
**Coevolution of carcinoembryonic antigen related cell adhesion molecule (CEACAM)1-like members with immunoreceptor tyrosine-based inhibition motif (ITIM) and immunoreceptor tyrosine-based activation motif (ITAM)-like signalling motifs.** The phylogeny of CEACAM1-like CEA family members from opossum (A), cattle (B), dogs (C) and humans (D) was reconstructed based on the amino acid sequences differences of mature N domains (minus leader signal peptide sequence) using the neighbour-joining method. The statistical support for each node is expressed as bootstrap values. Genes containing ITIM/immunoreceptor tyrosine-based switch motif encoding exons are marked with red boxes, such with ITAM and ITAM-like motif-encoding exons green and blue, respectively. Note, genes with ITIM or ITAM/ITAM-like signalling motif exons are either the closest relatives (B, C) or belong to the same subgroup of closely related members (A, D). Both opossum and humans contain a group of highly similar genes which code for secreted (human pregnancy-specific glycoproteins) or apparently secreted proteins. Bars below the phylogenetic trees indicate the scale for the number of substitutions per site.a, b, pseudogene

Human and rodent genomes comprise a cluster of genes (human: *PSG1-PSG10*; mouse: *Psg16-Psg32, Ceacam9, Ceacam11-Ceacam15*; rat: *Psg36-Psg45, Ceacam9, Ceacam11-Ceacam12; Ceacam15*) which encode secreted proteins specifically expressed in trophoblast cells (shown as red arrowheads in Figure 3 and data not shown). No such genes could be identified in the dog. Structure alone, however, is not sufficient to place a CEACAM1-like protein into the PSG group, since secreted CEACAM1-related proteins, like CEACAM10, exist in the mouse which is not expressed in trophoblast cells [5,31]. Bearing this uncertainty in mind, we searched the genomes for genes encoding secreted CEACAM. We were able to detect PSG-related genes in the human, chimpanzee, orangutan, rhesus macaque, African green monkey (*Chlorocebus aethiops*) and the baboon (*Papio hamadryas*). Surprisingly, no PSG-related genes could be found in more distantly related primates such as the small-eared galago (*Otolemur garnettii*) and the grey mouse lemur (*Microcebus murinus*). However, at least five equine *CEACAM*-related genes might represent *PSG *genes since they encode most probably secreted placentally expressed proteins consisting of a single N domain (Additional File 2, part A). Evidence for placental expression is based on the identification of two EST sequences in horse trophoblast cDNA libraries. The extreme expansion of the small brown bat *CEA *gene family is mostly due to the amplification of genes, probably encoding small secreted proteins composed of one N and one IgC-like A domain. The latter domains are encoded by A exons with homologous defective splice donor sites directly followed by two in-frame stop codons (AT**TAGTGA**; G to A and G to T mutations are underlined; stop codons in bold letters) (Additional File 2, part B). The opossum might also contain a group of closely related secreted proteins (Figures 2 and 6).

### GPI anchored CEACAMs are predominantly found in primates

Two phylogenetically related hydrophobic membrane domains have been identified in eutherian CEACAM1-like proteins: transmembrane domains found in family members with an ITIM/ITSM-related or with an ITAM-like signalling motif in their cytoplasmic domains [4]. Presently it is thought that the independent introduction of stop codons (and further mutations) into transmembrane exons associated with ITIM-encoding exons of old world (catarrhini) and new world monkeys (platyrrhini), led to the formation of exons coding for hydrophobic membrane signal sequences for GPI anchorage [32]. In humans and great apes, as well as in more distantly related primates (such as the small-eared galago), such signal peptide sequences are present in various CEACAMs (for example, in human CEACAM5-CEACAM8) but have not been detected in rodents, dogs, horses or cattle. Surprisingly, there is evidence that a GPI-linked CEACAM exists in swine. Three different EST sequences, derived from intestinal cDNA libraries, indicate that three nucleotides were deleted from an ancestral C*EACAM1 *transmembrane domain exon and a stop codon six nucleotides downstream was introduced by mutations exactly as found in the New World monkeys *Ateles geoffroyi *and *Callicebus molloch *(Additional File 3; [32]). This contrasts with the formation of GPI signal peptide-encoding exons in Old World monkeys where a single nucleotide deletion (followed directly by a stop codon) occurred. This indicates repetitive independent formation of signal sequences for GPI anchorage.

### Coevolution of CEACAM1-like members with ITIM and ITAM-like signaling motifs

**Figure 7:**
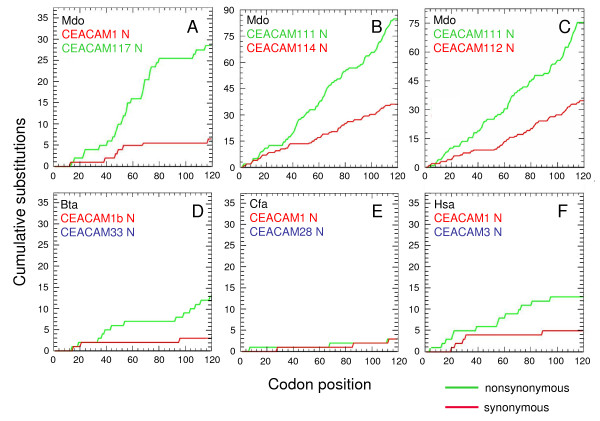
**Evidence for gene conversion/recombination between putative paired carcinoembryonic antigen related cell adhesion molecule *(CEACAM) *receptor genes**. The accumulation of nonsynonymous (red curve) and synonymous substitutions (green curve) along immunoglobulin (Ig) variable- and Ig constant-like domain exons of putative paired CEACAM1-like receptors were determined after manual removal of sequence gaps. The type of encoded signalling motif is indicated by the colour of the gene name: red, immunoreceptor tyrosine-based inhibition motif/immunoreceptor tyrosine-based signal motif; green, immunoreceptor tyrosine-based activation motif (ITAM); blue, ITAM-like motif. Stretches of codons with no or minimal accumulation of synonymous substitutions suggest recent recombination events. No such recent events are evident for the putative opossum paired receptors CEACAM114 and CEACAM111 and CEACAM112 and CEACAM111. For abbreviation of species names see Table 1.

**Figure 8:**
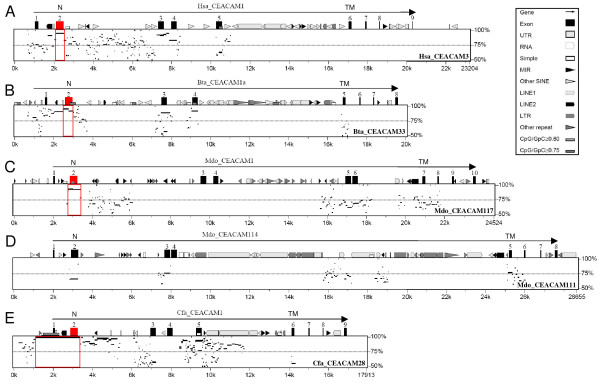
**Delineation of regions with gene conversion/recombination events between putative paired carcinoembryonic antigen related cell adhesion molecule *(CEACAM) *receptor genes**. Nucleotide sequences of immunoreceptor tyrosine-based inhibition motif (ITIM)/immunoreceptor tyrosine-based switch motif (ITSM)-encoding *CEACAM *genes from humans (A), cattle (B), opossum (C, D) and dogs (E) were compared with the sequences of *CEACAM *genes coding for their most closely related, putative paired receptor (gene names are shown in the lower right corner of the plot). For contiguous stretches of nucleotides conserved between gene pairs, the degree of identity was calculated and displayed as horizontal lines. The location of exons is indicated by numbered boxes. Genomic regions involved in gene conversion/recombination are marked with red boxes, involved N exons are shown in red. Note that these regions exhibit the highest degree of conservation. Repeat sequences indicated by differently shaped forms (see box for detailed explanation) have preferentially accumulated in most ITIM/ITSM-encoding genes between the immunoglobulin constant-like domain and the transmembrane domain exons. N, N domain exon; TM, transmembrane domain exon.

We have recently discovered potential paired receptors in the canine CEA family [4]. Such extracellularly very similar receptors contain oppositely signalling (inhibitory versus activating) motifs in their cytoplasmic domains [33]. They can be part of defense mechanisms against bacterial pathogens as has been found for the human CEA family members CEACAM1 and CEACAM3. In order to find out whether potential paired CEACAM receptor systems also exist in other species, we analysed the phylogeny of N domain amino acid sequences of CEACAM proteins from opossum and cattle and determined the presence of ITIM and ITAM/ITAM-like motifs in their cytoplasmic domains. As in humans and dogs, CEACAM members with cytoplasmic ITIM and members with ITAM-related motifs are always found in the same group of closely related members (Figure 7). The close similarity between paired receptors during evolution can be maintained by gene conversion which often involves recombination events within, or in the neighborhood of, exons encoding the ligand-binding domains [34]. In order to identify regions where such events may have taken place, we calculated cumulative synonymous and nonsynonymous substitutions along the N domain exons from pairs of *CEACAM1*-related paralogous genes coding for potential paired receptors. Analysis of the cumulative synonymous substitutions along the exons of expressible genes indicated that recombination events between ITIM and ITAM-carrying paralogs leading to very similar N domain amino acid sequences has taken place during evolution for all paired receptor candidates except for the *CEACAM114/CEACAM111/CEACAM115 *or *CEACAM112/CEACAM111/CEACAM115 *set of opossum genes. This is suggested from the stretches of codons with no or minimal accumulation of synonymous substitutions (Figure 6A and 6D-F). The corresponding N domain amino acid sequences probably contain segments which are important for (bacterial) ligand binding. An almost steady accumulation of synonymous substitutions is observed for *CEACAM114*/*CEACAM112 *(ITIM) and *CEACAM111 *or *CEACAM115 *genes (ITAM) from opossum which indicates a lack of recent recombinations between these genes (Figure 7B and 7C; only shown for *CEACAM111*). They might, therefore, represent a second paired receptor gene set created by gene duplication which became defunct or acquired different functions later during evolution. In order to narrow down the gene regions involved in the maintenance of the high similarity between the N domain sequences of putative paired receptors, we compared the nucleotide sequences of ITIM- and ITAM-encoding *CEACAM *genes using the *PipMaker *program. These analyses revealed that the paired receptor genes probably originated from gene duplication of ancestral *CEACAM1 *genes as indicated by long stretches of homologous exon and intron sequences including often transmembrane domain-encoding exons (Figure 8). In paired receptor genes of the opossum homology also encompasses the first cytoplasmic domain exon (Figure 8C and 8D). The last two (three in opossum) cytoplasmic domain exons from a *CEACAM *ancestor encoding ITIM/ITSM have been replaced by four (three in opossum) cytoplasmic domain exons from an ITAM-encoding ancestor. Interestingly, in all receptor pairs (with the exception of the opossum *CEACAM114*/*CEACAM111 *genes; Figure 8D) the highest similarity is found for the N domain exon region, barely extending into the flanking intron sequences (Figure 8A-C). In the canine *CEACAM1*/*CEACAM28 *receptor gene pair, however, the high similarity region covers the first intron, the leader exons as well as about 1 kb of the upstream sequences (Figure 8E).

### Variability of the extracellular domain organization of CEACAM1-like proteins

**Figure 9:**
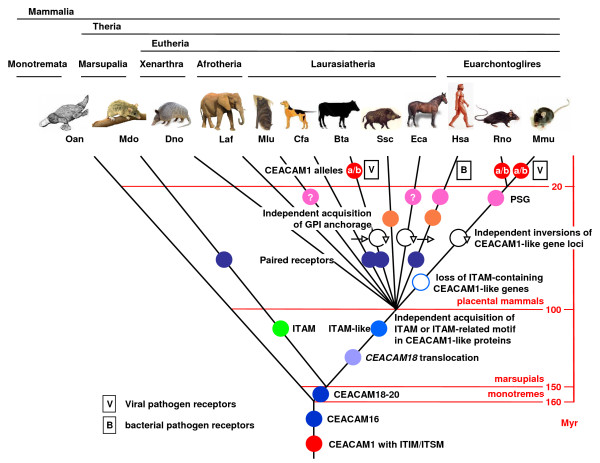
**Gain and loss of carcinoembryonic antigen related cell adhesion molecule (CEACAM) family features in mammals during evolution**. The phylogenetic and taxonomic relationship of selected mammalian species is shown schematically. Major radiation events with estimated radiation times in million years (Myr) are indicated [55]. The appearance of various features during radiation is marked by coloured dots. Linear and circular arrows indicate the absence or presence of an inversion event between the two main *CEACAM1*-like gene loci (see Figure 3 for details). V, B (boxed) indicate that CEACAM1 serves as bacterial (B) or viral (V) pathogen receptor in the marked lineage. Question marks in the pregnancy-specific glycoproteins dots indicate that the assignment to this CEACAM1-like group is still unclear due to a lack of expression data. For abbreviation of species names see Table 1.

In orthologous members, the domain numbers and arrangement are mostly conserved, with the exception of CEACAM1 which contains two to four IgC-like domains caused by exon deletion (Figure 2). In contrast, an extremely large variability of domain type and arrangement is observed for the CEACAM1-like members. Proteins expressed in trophoblast tissues vary in their number of IgV-like and IgC-like domains (Figure 2). So far, proteins with multiple contiguous N domains are only predicted to exist in rodents (trophoblast-expressed proteins) and platypus (CEACAM16-like protein). Non-PSGs are mostly composed of one IgV-like N domain and a variable number (0-6) of IgC-like domains, with the exception of CEACAM10 which contains two N domains in the mouse (Figure 9).

## Discussion

This comprehensive study of *CEA *gene families from more than 20 mammalian species, including members from all four eutherian clades as well as from a monotreme and a marsupial, revealed that a number of features were repeatedly acquired or lost during the evolution of this relatively young gene family (summarized in Figure 9). However, the birth of the *CEA *gene family during evolution is not yet clear. *CEA*-related genes have been identified in various fishes, the most distantly related vertebrate species analysed to date (WZ, unpublished results). Although sequence conservation between fish and mammalian *CEACAM *genes is low, the assignment to the *CEA *gene family is strongly supported by their syntenic location and conservation of intron phasing which is not conserved between Ig superfamilies in general. Since *CEA *family genes which code for proteins with ITIM/ITSM have been found in fishes, a *CEACAM1*-like gene probably represents the ancestor of the family (WZ, unpublished results). Surprisingly, the other conserved members of the *CEA *family (*CEACAM16, CEACAM18, CEACAM19, CEACAM20*) with identifiable orthologs have not so far been found outside the mammalian class (such as birds, amphibians, fishes; WZ, unpublished results). Therefore, *CEACAM18, CEACAM19 and CEACAM20 *probably did not evolve in therians until separation of the monotreme lineage.

The ancestral *CEA *gene family probably arose from a single locus next to the *BCL3 *gene by successive gene duplication. In marsupials, as exemplified by the opossum, this primordial arrangement has been mostly maintained except for *CEACAM1 *and four other closely linked *CEACAM1*-like genes which were displaced to chromosome 2. In the ancestor of placental mammals, the insertion of Zn finger and other genes separated the orthologous genes from the *CEACAM1*-related genes. The latter genes were split into two loci by non-*CEACAM *genes (Figure 3). This arrangement might help to create and maintain closely related paired ITAM- or ITIM-containing receptors (see below).

The sequence similarity of N domains, which are presumably instrumental for the function of all CEA-related proteins, differs dramatically among the ancestral genes with *CEACAM16 *being the most highly and *CEACAM1 *the least conserved member. This is evident from analyses of synonymous (dS) and nonsynonymous substitution rates (dN) within the nucleotide sequences of their N domain exons (Figure 4). The observed dN/dS ratio of about 0.1 for the *CEACAM16 *N2 domain exons indicates a high selective pressure on the conservation of this domain while the dN/dS ratio of ~1 found for *CEACAM1 *N domain exons suggests no selection at all. However, taking into account the differential sequence conservation observed along the N domain exon sequences for *CEACAM1 *(Figure 4C), selection for diversification and purifying selection for structurally important regions probably exist side by side in the N domain regions leading to an average dN/dS value of ~1. CEACAM1 N domain regions corresponding to the CFG face (codons 41-72 and 99-111; Figure 4C and Additional File 1) are the least conserved and probably represent regions selected for diversification, which is consistent with the CFG face being responsible for (bacterial and viral) ligand binding [30,35]. Selection for diversification is not observed for all the mammalian species analysed (which is indicated by the high standard deviation of the mean of the dN/dS values), possibly indicating lack of, or only intermittent interaction of, CEACAM1 with pathogens during evolution of these species (data not shown).

Through purifying selection during evolution only sequence positions which provide the bearer with an adaptive advantage are retained. Multispecies amino acid sequence comparison of the cytoplasmic regions of CEACAMs, therefore, allowed identification of sequence motifs which are indispensable for signalling of CEACAM receptors. In all species analysed, a membrane proximal THEKxS motif has been conserved in the CEACAM1 cytoplasmic domain which has been recently shown to serve as an interaction site for β-catenin [36]. In this study, mutational analysis revealed that the conserved histidine (H) and, to a lesser degree, the lysine (K) are most instrumental for the specific interaction of CEACAM1 with β-catenin. This possibly links CEACAM1 to the Wnt signalling pathway which could explain CEACAM1's role in differentiation and tumour formation upon downregulation [36,37]. Furthermore, an ITIM/ITSM set is conserved. Interestingly, in catarrhinian apes, including humans, the ITSM of CEACAM1 has been replaced by an ITIM. The ITSM can convey both stimulatory and inhibitory signals, while ITIM predominantly transmit inhibitory signals [38]. ITSM signaling is mediated by the signaling lymphocyte activation molecule-associated protein (SAP) which does not bind to ITIM [39]. More distantly related primates (strepsirrhini) still contain the ancestral ITSM. Therefore, the second ITIM has been acquired by a common ancestor of catarrhinian apes, after the divergence from the strepsirrhinian ancestor. The functional consequences of this modification of CEACAM1 have not yet been systematically addressed. However, in a previous study we noticed that targeting CEACAM1 on human T cells with monoclonal antibodies enhances T cell activation. The molecular mechanisms of the dual function of CEACAM1 in human T cells is still unclear but may be an functional adaption to the acquisition of a second ITIM in its cytoplasmic tail [12]. Surprisingly, such a switch occurred several times during mammalian evolution, for example, *bona fide *ITIM are also present in platypus and opossum (Figure 5A). Most of the genes coding for transmembrane-anchored eutherian CEACAM1-related proteins contain a set of four phylogenetically related cytoplasmic domain exons which encode a non-canonical ITAM (YxxLx_7_YxxI/M). In two members (human CEACAM3 and a swine CEACAM1-related protein, provisionally named CEACAMa) the second part of the ITAM represents an YxxM motif known to mediate Vav binding upon tyrosine phosphorylation. This can be induced by the binding of neisserial pathogens to the granulocyte-specific CEACAM3 followed by rapid stimulation of the GTPase Rac, eventually leading to phagocytosis and killing of the bacteria [20]. Whether the ITAM-like motifs with an isoleucine instead of the methionine found in the CEACAM1-like proteins of other species are functionally equivalent and also mediate phagocytosis of bacterial pathogens is presently unclear. Another prerequisite for such a function would be expression in granulocytes. This has at least been demonstrated for most of the canine CEACAM1-related proteins with ITAM-like motifs [4]. In the marsupial lineage (opossum, wallaby) *CEACAM1*-related genes could be identified which also contain four cytoplasmic domain exons with identical intron phasing found in eutherian CEACAM1-related proteins with ITAM-like motifs. Interestingly, these exons encode conventional ITAM (ExxYxxLx_7_YxxI) and, since they differ in size, sequence and orthology, they appear to have been independently acquired from a different ancestral gene. This is underscored by the presence of a conserved GRB2-like SH2 domain-binding motif (YxN) which is not present in the eutherian CEACAM1-related proteins with ITAM-like motifs (Figure 5C). Interestingly, a similar YxN motif is encoded by the same sized cytoplasmic domain exon 2 of *CEACAM20 *which suggests common ancestry [5]. Therefore, marsupials have probably evolved or maintained additional signalling capabilities in their CEACAM1-like proteins.

The most obvious difference between mammalian *CEA *gene families is their extreme size variation of the *CEACAM1*-related members with gene numbers ranging from less than five in pika rabbit to more than 100 in bat (Table 1). Identification of low numbers of *CEACAM1*-related genes is probably not due to low coverage of the genome sequencing in some species, since high and low gene numbers can be found in species with both low and high coverage sequencing. Most of the size variation is due to the variable number of *CEACAM1*-related genes with ITAM-like motifs and *CEACAM1*-related genes encoding secreted PSG-related proteins (Figures 1 and 2). Murine and human PSGs have been shown to have immunoregulatory functions due to their ability to modulate cytokine secretion of macrophage and monocytic cells. In mice, CD9 serves as a receptor for PSG17 and PSG19 [24,40]. Murine PSG23, on the other hand, induces the proangiogenic factors transforming growth factor β1 (TGFβ1) and vascular endothelial growth factor A (VEGFA) in cell types involved in vascular remodelling in pregnancy independent of CD9 [41]. This supports the notion that PSGs play a diverse role in the protection of allotypic fetus from maternal immune system, as well as in the promotion of invasive growth of cytotrophoblast, and in the remodelling of the maternal vasculature in order to gain optimal access to maternal resources. Indeed, *PSG *genes have so far been found only in species with haemochorial placentation (primates and rodents), a type of placentation with the most intimate contact of fetal trophoblast cells with maternal (immune) cells. As reported here, the only other species with candidate *PSG *genes is the bat (*M. lucifugus*) and the horse comprising at least 18 and five potential PSGs, respectively (Additional File 2). Interestingly, the microbat *M. lucifugus *also exhibits highly invasive haemochorial placentation [42]. Although the horse has an epitheliochorial placenta without the direct contact of the fetal trophoblast cells with maternal immune cells, a specialized highly invasive trophoblast cell population exists that forms so-called endometrial cups responsible for the secretion of chorionic gonadotrophin in the maternal decidua. These fetal trophoblast cells have to survive there, despite being immunogenic and recognized by the maternal immune system [43]. Therefore, it is conceivable that PSG secreted by these cells suppress the maternal immune system locally. Indeed, we could identify two partial cDNAs from invasive and non-invasive trophoblast cells of an equine candidate PSG in EST data bases. Both, the need for diversification of functions as seen in the mouse and a possible maternal-fetal conflict scenario would favour the expansion of *PSG *genes [35].

Highly sophisticated regulatory circuits have been developed during evolution to enable the host to cope efficiently with pathogenic insults and, at the same time, to avoid overreactions of the immune system. Inhibitory (ITIM) as well stimulatory sequence modules (ITAM) in immune regulatory proteins are instrumental in these processes. The paired receptors within the CEA family could be part of this regulatory system [4]. Indeed, the ITIM-containing isoform of CEACAM1 has been shown to downregulate innate and adaptive immune responses [16,44,45]. This might apply to humans and dogs where ITAM-like motif-bearing members (human - CEACAM3 and probably CEACAM4; dog - CEACAM25, CEACAM28, CEACAM30) are coexpressed with CEACAM1 in granulocytes.

Pathogens exploit this system in order to interfere with efficient immune responses. Expression of ligands in virus-infected cells, which downregulate the immune response via inhibitory receptors, has been identified as immune evasion mechanisms. For example, binding of the MHC class I-related proteins UL18 encoded by human cytomegalovirus (CMV) and m157 from murine CMV to the inhibitory leukocyte Ig-like receptor (LILR) B1 or LILRB4 and Ly49I, respectively, inhibit NK cells [3,46]. This counteracts the recognition of virus-infected cells in which the MHC class I antigen presentation machinery is often suppressed upon virus infection since the MHC class I-related protein m157 is not able to present (viral) peptides [2].

In this respect, CEACAM1, from a pathogen point of view, represents an ideal receptor since it allows both easy entry into the host due to its wide-spread expression in epithelia and, at the same time, suppression of the adaptive and innate immune system. Upon engagement with pathogenic bacteria, CEACAM1 acts as inhibitory receptor and down-regulates T cell receptor and TLR2-mediated signalling in CD4-positive T cells and pulmonary epithelial cells, respectively [15,16]. However, various counter strategies have been developed by the hosts to fend off CEACAM1-binding pathogens. To date, two strategies have been well characterized: allelic variation of CEACAM1 or formation of a paired internalization receptor. An allelic variant of CEACAM1 with lowered pathogen ligand affinity has been identified for mouse hepatitis virus (see Introduction). This pathogen-host 'arms race' might be more wide-spread since *CEACAM1 *alleles which encode highly divergent N domains have also been identified in rats and cattle [27,47]. Indeed, recently, a candidate viral pathogen has been found to use bovine CEACAM1 as a receptor (K Holmes, personal communication). On the other hand, CEACAM3 has been found to represent an opsonin-independent granulocyte receptor for internalization of neisserial pathogens. The trapping of pathogens by human GPI-anchored CEACAM1-related proteins on microvili of colonic enterocytes, such as CEA and CEACAM6, which also bind neisserial pathogens, followed by the shedding of vesicles from the CEACAM-positive microvili or hydrolysis of the GPI linker has been suggested by Hammarström and Baranov as an additional defense mechanism involving CEACAM1-related proteins [48]. A countermeasure against downregulation of immune responses by pathogens via inhibitory receptors has been observed with other receptor systems: the formation of paired activating receptors. A well studied example of such a mechanism is the generation of the NK receptor Ly49H from its inhibitory killer cell receptor which renders carrier strains resistant to murine CMV infections [2,3]. Paired receptors are common in killer Ig-like receptor (KIR), LILR and sialic acid Ig-like lectins (Siglec) gene families in the human extended LRC [33]. Most of the positive signalling receptors from these receptor families associate via a characteristic positively charged amino acid in their transmembrane domain with the ITAM-containing adaptor DAP12 [49,50]. CD33r Siglec-14/Siglec-5 and Siglec-16/Siglec-11 represent such paired receptors, which contain very similar extracellular domains [34,49]. It is postulated that binding to pathogen-derived sialic acid might be the driving force behind the formation of these paired receptors [49].

Our studies indicate, that paired receptors also exist in the CEA family, which might share endogenous or pathogen ligands (Figure 9). At present it is not clear whether the ITAM-like motifs found in the paired CEACAM receptors represent internalization or activation signals as found in KIR, LILR and Siglec paired receptors. Pathogen uptake experiments involving domain swapping of the CEACAM3 cytoplasmic domain with those of other ITAM-like motif containing members have to be performed to clarify this issue. On the other hand, opossum ITAM-containing CEACAMs together with their inhibitory receptors (CEACAM1 and CEACAM114) could represent *bona fide *paired receptors. Although a number of bacterial pathogens have been demonstrated to gain access to epithelial cells of their host through binding to CEACAM1, phagocytosis by neutrophilic granulocytes via CEACAM3 has been evaluated only for one bacterial strain (*Neisseria gonorrhoeae*) [18]. Analysis of internalization through CEACAM3 of a wider selection of CEACAM1-binding bacterial pathogens would strengthen the proposed hypothesis.

Ligand binding domains of decoy receptors and corresponding receptors have to be kept similar enough during evolution to allow efficient interaction with pathogens. This can be achieved among others by gene conversion, recombination and gene duplication. Inversion of all non-*CEACAM *genes between the *CEACAM1*-related gene loci which occurred independently in species from different clades indicates that a recombination between closely related members of the different *CEACAM1*-related gene subclusters with inverted orientation has occurred, possibly leading to the formation of paired receptor genes (Figure 3). This observation strongly supports the notion that continuous selective pressure exists to maintain close similarity between the immune regulatory CEACAM1 with its ITAM-like motif-containing counterpart(s) or secreted proteins (CEACAM10 in rodents) which might serve as soluble decoy receptors. However, the formation of paired receptor genes might also occur by an illegitimate recombination between sister chromatids. Such a mechanism possibly has been at work in the dog where the ITAM-like motif-containing *CEACAM28 *which encodes an N domain nearly identical with that of *CEACAM1 *lies next to the canine *CEACAM1 *gene in the same subcluster [4]. The target gene was possibly created by gene duplication of the closely related neighbour gene (*CEACAM29*) which then exchanged a 2.2 kb DNA fragment comprising the leader and the N domain exons with *CEACAM1 *to form *CEACAM28 *(Figures 3 and 8E). Interestingly, *Siglec *and *LILR *genes are also arranged in two inverted clusters [49,51]. *LILR *subclusters too are interrupted by non-*LILR *genes like the *CEACAM1*-like genes in eutherian mammals. It is assumed that this arrangement might facilitate intrachromosomal gene conversion events thus avoiding or minimizing the 'concertino effect' of expansion and contraction of tandemly repeated gene families [49].

In summary, abuse of the inhibitory receptor CEACAM1 by viral and bacterial pathogens to gain access to host cells as well as the paternal/maternal conflict in which PSGs possibly are involved have probably strongly contributed to the divergent evolution of the CEA family.

## Conclusions

The *CEACAM *gene family which appears to be restricted to vertebrates exhibits an extreme diversity between different species and varies widely in the number (from five in lagomorphs to > 100 in bats) and type of members even among species from the same clade. Phylogenetic analyses of the *CEA *families from over 20 mammalian species revealed two mechanisms which could be responsible for the evolutionary diversity: pressure exerted by pathogens binding to CEA members and the possible involvement in fetal-maternal conflict processes of members expressed in the fetal trophoblast. CEACAM1 which acts as receptor for viral and bacterial pathogens in several species and mediates both pathogen access to host cells and inhibition of anti-pathogen immune response seems to be central for the diversification process. This is apparent from the selection for the diversification of the CEACAM1 pathogen binding domain, as well as from the formation of paired receptors with closely related pathogen binding domains by gene conversion and intrachromosomal recombination and ITAM-like or GPI anchorage motifs which allow internalization and destruction or shedding of bound pathogens, respectively. Birth and death of such genes contribute to diversity of the *CEA *gene family. Haemochorial placentation which involves invasive trophoblast growth correlated with the presence of *CEACAM1*-related often numerous *PSG *genes in the species investigated. The trophoblast-specific PSG have been shown to have an immunomodulatory function, thus involvement in maternal-fetal conflict processes can be envisaged. Expansions of *PSG *gene families could counteract a possible reduction of the affinity of maternal receptors by mutation. Taken together, our findings should aid identification of CEACAM pathogen receptors in other species and possibly lead to the identification of additional gene families with immunoregulatory members the evolution of which is driven by host/pathogen coevolution or fetal-maternal conflict processes.

## Methods

### Datasets and nomenclature of genes

Sequence similarity searches were performed using the NCBI BLAST tools http://www.ncbi.nlm.nih.gov/BLAST and Ensembl BLAST/SSAHA search programs http://www.ensembl.org/Multi/blastview. For identification of *CEACAM *genes and exons, in part unannotated genomic and cDNA sequences from humans, mice and dogs, *CEACAMs *as well as human and mouse *PSGs *were run against the whole genome shot gun sequence databases, NCBI genomes and Ensemble genome builds. Individual genomes were reprobed with corresponding exon sequences from newly discovered genes. For estimation of the number of *CEACAM1*-like genes present in a given species, distinct *CEACAM1*-like N domain exons with a sequence divergence > 1% were counted. No evidence was found that genes with multiple N domain exons as found in rodents exist in any of the newly analysed species. Genes that contained stop codons within their N domain exons or lacked appropriate splice acceptor and donor sites were considered to represent pseudogenes. For opossum, *CEACAM *genes with a corrupted leader peptide exon were also registered as pseudogenes. Genes were assigned to their respective groups based on phylogenetic analyses. Their assignment was confirmed by synteny and gene structure. Due to their non-orthologous relationship the new *CEACAM1*-like genes were numbered as follows: cattle, *CEACAM32*-*CEACAM35*; horses, *CEACAM41*-*CEACAM51 *and opossum, *CEACAM100*-*CEACAM122*. Nucleotide sequences from the N domain exons can be used as gene identifier for the non-annotated genes (Additional File 4). Genes with partial exon characterization were named provisionally (*CEACAMa, CEACAMb*). The following Ensembl/NCBI releases were used: platypus, Ornithorhynchus_anatinus-5.0; opossum, MonDom5; human, NCBI 36; mouse, NCBI m36; Cattle, Btau_4.0; dog, CanFam 2.0; horse, EquCab2.

### Sequence motif identification

The presence of ITAM and ITIM were confirmed using the amino acid sequence pattern search program ELM http://elm.eu.org/. Transmembrane, glycosylphosphatidyl inositol (GPI) signal domains and leader peptide sequences were identified using the TMHMM http://www.cbs.dtu.dk/services/TMHMM-2.0/, the big-PI predictor http://mendel.imp.ac.at/gpi/gpi_server.html, GPI-SOM http://gpi.unibe.ch/ and the SignalP programs http://www.cbs.dtu.dk/services/TMHMM-2.0/, respectively [52]. Multiple amino acid sequence alignments were performed with ClustalW programs http://npsa-pbil.ibcp.fr/cgi-bin/npsa_automat.pl?page=/NPSA/npsa_clustalw.html.

### Phylogenetic analyses

Phylogenetic analyses based on nucleotide and amino acid sequences were conducted using MEGA version 4 [53]. The neighbour-joining (NJ) method with bootstrap testing (100 or 1000 replicates) and Poisson correction was applied. Unrooted phylogenetic trees based on amino acid sequences were constructed with ClustalW http://align.genome.jp/ using default parameters. N order to determine the selective pressure on the maintenance of the nucleotide sequences, the number of nonsynonymous nucleotide substitution per nonsynonymous site (dN) and the number of synonymous substitutions per synonymous site (dS) were determined for N, A and B exons. The dN/dS ratios were calculated after manual editing of sequence gaps or insertions guided by the amino acid sequences for all branches of the resulting phylogenetic trees using the Ka/Ks Calculation tool (Bergen Center for Computational Science, Computational Biology Unit, Bergen, Sweden; http://services.cbu.uib.no/tools/kaks). Due to the variable truncation of the *CEACAM20 *N domain exons in different species, the first common 27 codons were analysed. The mean dN/dS ratios and standard deviations were calculated. The SNAP program (Synonymous Nonsynonymous Analysis Program; http://www.hiv.lanl.gov/content/sequence/SNAP/SNAP.html) allowed the calculation of cumulative average synonymous and nonsynonymous substitutions along coding regions of N domain exons from paralogous and orthologous genes. The program *PipMaker *http://bio.cse.psu.edu/ was used to identify conserved contiguous stretches of nucleotides between gene pairs and to calculate the degree of identity which is summarized as a 'percent identity plot' [54].

## Abbreviations

CEA: carcinoembryonic antigen; CEACAM: carcinoembryonic antigen-related cell adhesion molecule; Cyt: cytoplasmic domain; GPI: glycosylphosphatidyl inositol; Ig: immunoglobulin; IgC: Ig constant; IgSF: Ig super family; IgV: Ig variable; ITAM: immunoreceptor tyrosine-based activation motif; ITIM: immunoreceptor tyrosine-based inhibition motif; ITSM: immunoreceptor tyrosine-based switch motif; MHV: mouse hapatitis virus; NJ: neighbour joining; NK: natural killer; PSG: pregnancy-specific glycoprotein; TM: transmembrane domain.

## Competing interests

The authors declare that they have no competing interests.

## Authors' contributions

RK conceived the study, did most of the biocomputing and participated in manuscript writing. WZ participated in biocomputing and did most of the manuscript writing. Both authors participated in the design of the study, and both read and approved the final version.

## Supplementary Material

Additional file 1 **Figure S1 - Identification of functionally critical amino acid positions in N domains from carcinoembryonic antigen related cell adhesion molecule 1 (CEACAM1) by multispecies sequence alignments**. Amino acid sequences of mature CEACAM1 N domains without leader peptide were aligned using the program ClustalW. The following colour code was used: red, identical amino acids; green, conserved; blue, less conserved amino acids. Sequence gaps are depicted as dashes. The amino acid positions highly conserved in CEACAM1 which are probably important for the basic β-sheet structure are marked with red bars. Potential N-glycosylation consensus sequences are boxed. The conserved salt bridge characteristic for the CEACAM1 N domain is indicated by red brackets. The location of β-strands are indicated by arrows [30]. Regions involved in the formation of the CFG face are boxed with blue lines. For abbreviations of species names see Table 1. Click here for file

Additional file 2 **Figure S2 - Species-specific mechanisms for the generation of secreted carcinoembryonic antigen related cell adhesion molecules (CEACAMs)**. (A) Formation of secreted CEACAM proteins by truncation of A domains in the horse by nonsense mutations. Partial nucleotide sequence of A domain exons from putative secreted CEACAMs (composed of a leader, an N domain and an A domain) of the horse have been aligned. The sequences (truncation at the 3'-end is indicated by dots) are closely related except for CEACAM48. Stop codons are indicated in red. Homologous codons of CEACAM44, CEACAM46, CEACAM47 and CEACAM49 which could be changed into stop codons by one mutation are indicated in blue. Note that there is a common stop codon starting at nucleotide position 63 (TAA). Additional in-frame stop codons in *CEACAM46 *and *CEACAM44 *lead to a shortening of the A domain of the secreted molecule. (B) Formation of secreted CEACAM proteins by the mutation of the splice donor site of A domain exons in the microbat *Myotis lucifugus*. The splice donor site (consensus sequence is shown on top) is mutated (marked with the black box) leading to read-through into the intron and the generation of one or two in-frame stop codons (indicated in red and red boxes). Closely related A domain exons from whole genome shot gun sequences were aligned and a selection is depicted with indicated reading frame. The accession numbers are indicated in the left margin. The numbers to the left of the sequences indicate their positions within the shot gun sequence fragments. Click here for file

Additional file 3 **Figure S3 - Independent generation of glycosylphophatidyl inositol (GPI)-anchored carcinoembryonic antigen related cell adhesion molecules (CEACAMs)**. (A) Nucleotide sequences of exons encoding GPI anchor signal peptides from primate CEACAMs and swine CEACAMa as well as the exon sequences encoding the transmembrane domain of human CEACAM1 were aligned. Nucleotides conserved in all species are indicated in red. (B) Partial amino acid sequence of the hydrophobic GPI anchorage signal peptide proceeded by an A domain from swine CEACAMa. The predicted omega-site for signal peptide cleavage is indicated in red and a possible alternative site in yellow. The omega-sites and its probability were calculated with the *big-GPI predictor *program. Expression of Ssc_CEACAMa is supported by three EST sequences from intestinal cDNA libraries [GenBank: EW073632, EW434309, EV988922]. *Ateles geoffroyi *(Age) and *Callicebus molloch *(Cmo) sequences were taken from [32]. For abbreviation of the names of additional species see Table 1. Click here for file

Additional file 4 **Sequence data**. Nucleotide sequences from N domain exons as gene identifier for carcinoembryonic antigen related cell adhesion molecule (CEACAM) genes. Click here for file
