# Supplementary figures and images for: Coevolution of activating and inhibitory receptors within mammalian carcinoembryonic antigen families
Source: BMC Biol. 2010 Feb 4;8:12. doi: 10.1186/1741-7007-8-12 (PMC2832619; doi:10.1186/1741-7007-8-12)

## Slide 1
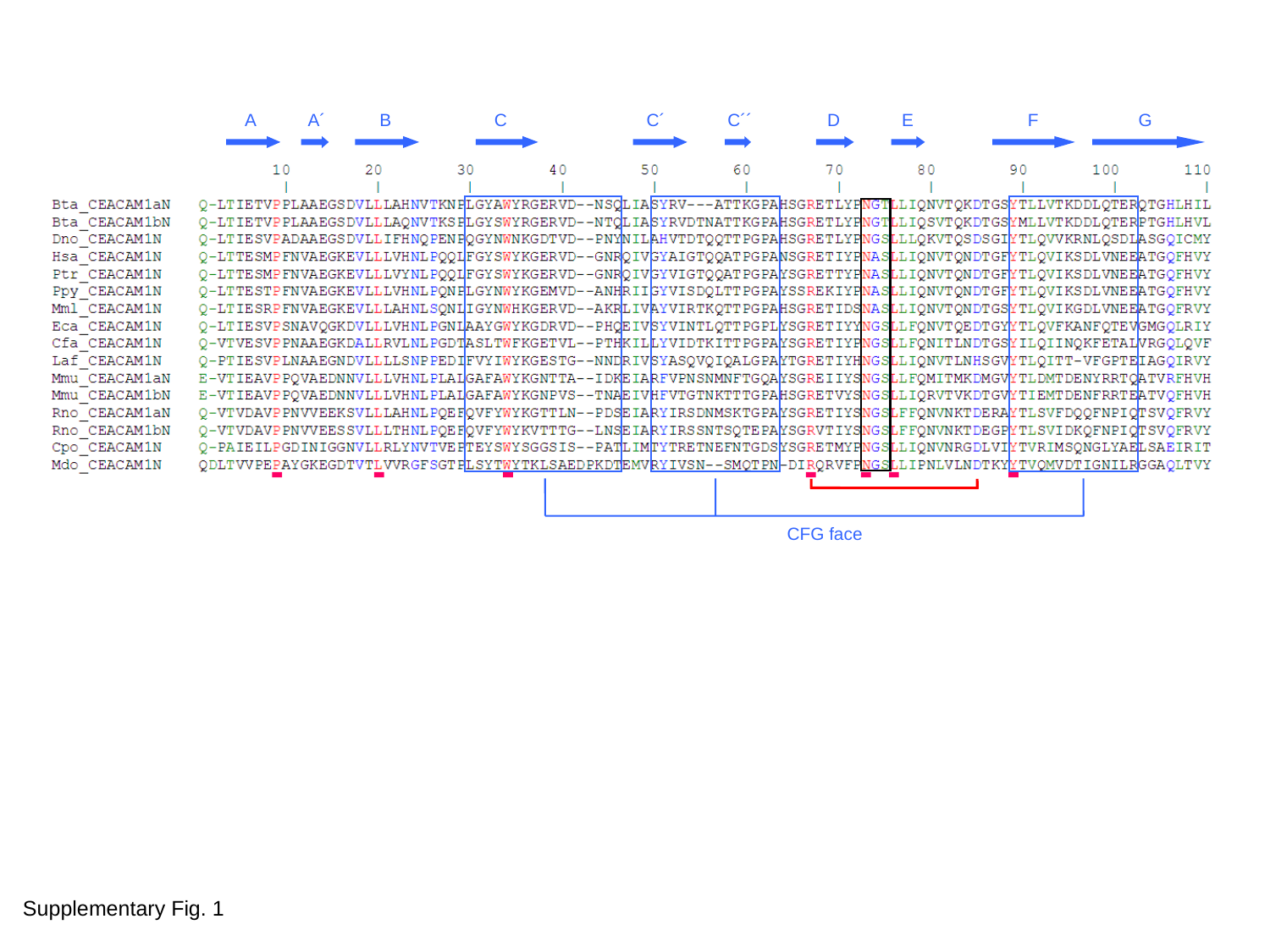

A
A´
B
C
C´
C´´
D
E
F
G
CFG face
Supplementary Fig. 1

Supplement: Additional file 1 — Figure S1 - Identification of functionally critical amino acid positions in N domains from carcinoembryonic antigen related cell adhesion molecule 1 (CEACAM1) by multispecies sequence alignments. Amino acid sequences of mature CEACAM1 N domains without leader peptide were aligned using the program ClustalW. The following colour code was used: red, identical amino acids; green, conserved; blue, less conserved amino acids. Sequence gaps are depicted as dashes. The amino acid positions highly conserved in CEACAM1 which are probably important for the basic β-sheet structure are marked with red bars. Potential N-glycosylation consensus sequences are boxed. The conserved salt bridge characteristic for the CEACAM1 N domain is indicated by red brackets. The location of β-strands are indicated by arrows [30]. Regions involved in the formation of the CFG face are boxed with blue lines. For abbreviations of species names see Table 1. [file 1741-7007-8-12-S1.PPT]
