# Supplementary material for: Coevolution of activating and inhibitory receptors within mammalian carcinoembryonic antigen families
Source: BMC Biol. 2010 Feb 4;8:12. doi: 10.1186/1741-7007-8-12 (PMC2832619; doi:10.1186/1741-7007-8-12)
Supplement: Additional file 2 — Figure S2 - Species-specific mechanisms for the generation of secreted carcinoembryonic antigen related cell adhesion molecules (CEACAMs). (A) Formation of secreted CEACAM proteins by truncation of A domains in the horse by nonsense mutations. Partial nucleotide sequence of A domain exons from putative secreted CEACAMs (composed of a leader, an N domain and an A domain) of the horse have been aligned. The sequences (truncation at the 3'-end is indicated by dots) are closely related except for CEACAM48. Stop codons are indicated in red. Homologous codons of CEACAM44, CEACAM46, CEACAM47 and CEACAM49 which could be changed into stop codons by one mutation are indicated in blue. Note that there is a common stop codon starting at nucleotide position 63 (TAA). Additional in-frame stop codons in CEACAM46 and CEACAM44 lead to a shortening of the A domain of the secreted molecule. (B) Formation of secreted CEACAM proteins by the mutation of the splice donor site of A domain exons in the microbat Myotis lucifugus. The splice donor site (consensus sequence is shown on top) is mutated (marked with the black box) leading to read-through into the intron and the generation of one or two in-frame stop codons (indicated in red and red boxes). Closely related A domain exons from whole genome shot gun sequences were aligned and a selection is depicted with indicated reading frame. The accession numbers are indicated in the left margin. The numbers to the left of the sequences indicate their positions within the shot gun sequence fragments. [file 1741-7007-8-12-S2.PPT]

## Slide 1
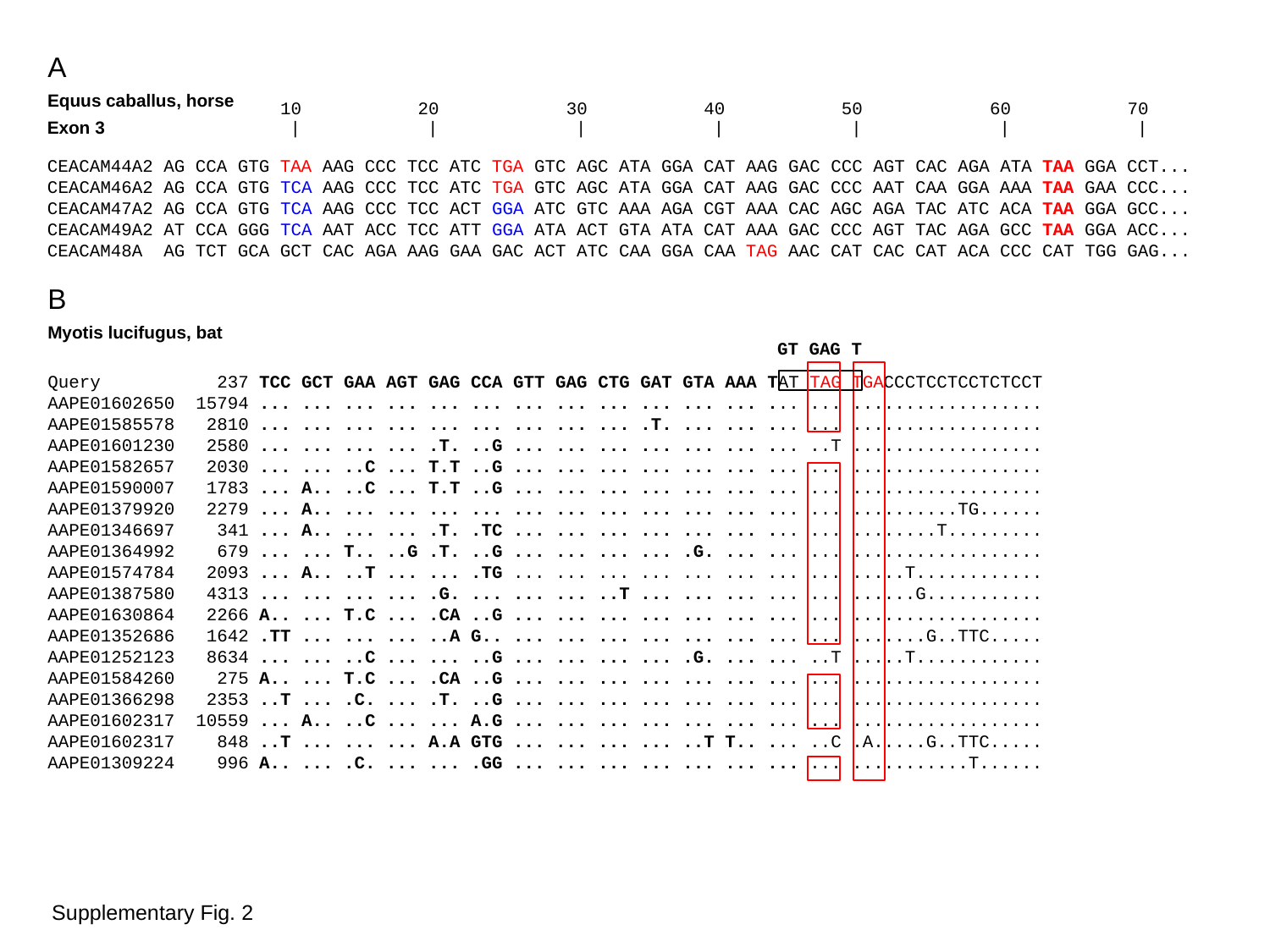

A
Equus caballus, horse
 10 20 30 40 50 60 70
Exon 3 | | | | | | |
CEACAM44A2 AG CCA GTG TAA AAG CCC TCC ATC TGA GTC AGC ATA GGA CAT AAG GAC CCC AGT CAC AGA ATA TAA GGA CCT...
CEACAM46A2 AG CCA GTG TCA AAG CCC TCC ATC TGA GTC AGC ATA GGA CAT AAG GAC CCC AAT CAA GGA AAA TAA GAA CCC...
CEACAM47A2 AG CCA GTG TCA AAG CCC TCC ACT GGA ATC GTC AAA AGA CGT AAA CAC AGC AGA TAC ATC ACA TAA GGA GCC...
CEACAM49A2 AT CCA GGG TCA AAT ACC TCC ATT GGA ATA ACT GTA ATA CAT AAA GAC CCC AGT TAC AGA GCC TAA GGA ACC...
CEACAM48A AG TCT GCA GCT CAC AGA AAG GAA GAC ACT ATC CAA GGA CAA TAG AAC CAT CAC CAT ACA CCC CAT TGG GAG...
B
Myotis lucifugus, bat
GT GAG T
Query 237 TCC GCT GAA AGT GAG CCA GTT GAG CTG GAT GTA AAA TAT TAG TGACCCTCCTCCTCTCCT
AAPE01602650 15794 ... ... ... ... ... ... ... ... ... ... ... ... ... ... ..................
AAPE01585578 2810 ... ... ... ... ... ... ... ... ... .T. ... ... ... ... ..................
AAPE01601230 2580 ... ... ... ... .T. ..G ... ... ... ... ... ... ... ..T ..................
AAPE01582657 2030 ... ... ..C ... T.T ..G ... ... ... ... ... ... ... ... ..................
AAPE01590007 1783 ... A.. ..C ... T.T ..G ... ... ... ... ... ... ... ... ..................
AAPE01379920 2279 ... A.. ... ... ... ... ... ... ... ... ... ... ... ... ..........TG......
AAPE01346697 341 ... A.. ... ... .T. .TC ... ... ... ... ... ... ... ... ........T.........
AAPE01364992 679 ... ... T.. ..G .T. ..G ... ... ... ... .G. ... ... ... ..................
AAPE01574784 2093 ... A.. ..T ... ... .TG ... ... ... ... ... ... ... ... .....T............
AAPE01387580 4313 ... ... ... ... .G. ... ... ... ..T ... ... ... ... ... ......G...........
AAPE01630864 2266 A.. ... T.C ... .CA ..G ... ... ... ... ... ... ... ... ..................
AAPE01352686 1642 .TT ... ... ... ..A G.. ... ... ... ... ... ... ... ... .......G..TTC.....
AAPE01252123 8634 ... ... ..C ... ... ..G ... ... ... ... .G. ... ... ..T .....T............
AAPE01584260 275 A.. ... T.C ... .CA ..G ... ... ... ... ... ... ... ... ..................
AAPE01366298 2353 ..T ... .C. ... .T. ..G ... ... ... ... ... ... ... ... ..................
AAPE01602317 10559 ... A.. ..C ... ... A.G ... ... ... ... ... ... ... ... ..................
AAPE01602317 848 ..T ... ... ... A.A GTG ... ... ... ... ..T T.. ... ..C .A.....G..TTC.....
AAPE01309224 996 A.. ... .C. ... ... .GG ... ... ... ... ... ... ... ... ...........T......
Supplementary Fig. 2
