# Supplementary material for: Coevolution of activating and inhibitory receptors within mammalian carcinoembryonic antigen families
Source: BMC Biol. 2010 Feb 4;8:12. doi: 10.1186/1741-7007-8-12 (PMC2832619; doi:10.1186/1741-7007-8-12)
Supplement: Additional file 3 — Figure S3 - Independent generation of glycosylphophatidyl inositol (GPI)-anchored carcinoembryonic antigen related cell adhesion molecules (CEACAMs). (A) Nucleotide sequences of exons encoding GPI anchor signal peptides from primate CEACAMs and swine CEACAMa as well as the exon sequences encoding the transmembrane domain of human CEACAM1 were aligned. Nucleotides conserved in all species are indicated in red. (B) Partial amino acid sequence of the hydrophobic GPI anchorage signal peptide proceeded by an A domain from swine CEACAMa. The predicted omega-site for signal peptide cleavage is indicated in red and a possible alternative site in yellow. The omega-sites and its probability were calculated with the big-GPI predictor program. Expression of Ssc_CEACAMa is supported by three EST sequences from intestinal cDNA libraries [GenBank: EW073632, EW434309, EV988922]. Ateles geoffroyi (Age) and Callicebus molloch (Cmo) sequences were taken from [32]. For abbreviation of the names of additional species see Table 1. [file 1741-7007-8-12-S3.PPT]

## Slide 1
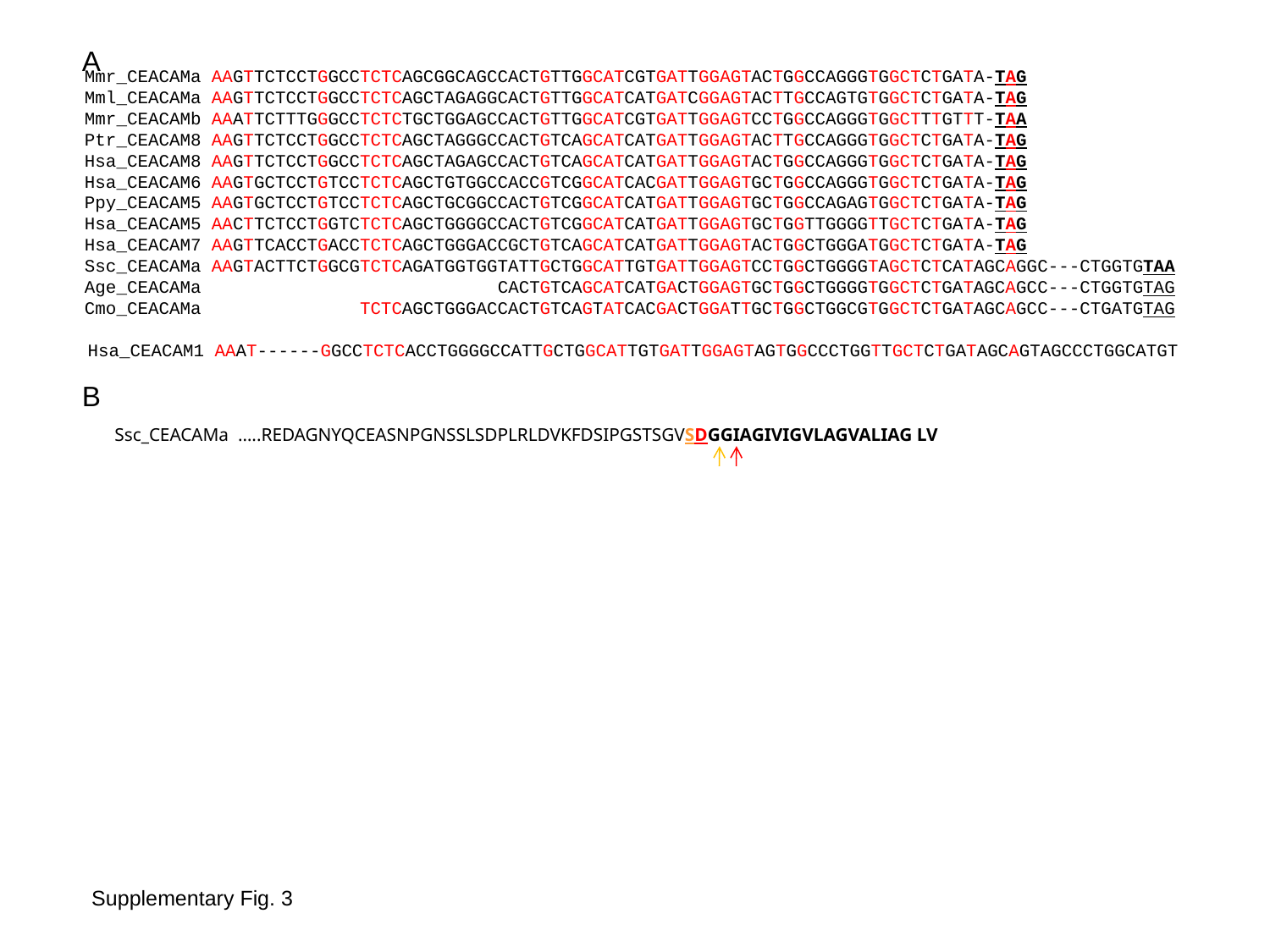

A
Mmr_CEACAMa AAGTTCTCCTGGCCTCTCAGCGGCAGCCACTGTTGGCATCGTGATTGGAGTACTGGCCAGGGTGGCTCTGATA-TAG
Mml_CEACAMa AAGTTCTCCTGGCCTCTCAGCTAGAGGCACTGTTGGCATCATGATCGGAGTACTTGCCAGTGTGGCTCTGATA-TAG
Mmr_CEACAMb AAATTCTTTGGGCCTCTCTGCTGGAGCCACTGTTGGCATCGTGATTGGAGTCCTGGCCAGGGTGGCTTTGTTT-TAA
Ptr_CEACAM8 AAGTTCTCCTGGCCTCTCAGCTAGGGCCACTGTCAGCATCATGATTGGAGTACTTGCCAGGGTGGCTCTGATA-TAG
Hsa_CEACAM8 AAGTTCTCCTGGCCTCTCAGCTAGAGCCACTGTCAGCATCATGATTGGAGTACTGGCCAGGGTGGCTCTGATA-TAG
Hsa_CEACAM6 AAGTGCTCCTGTCCTCTCAGCTGTGGCCACCGTCGGCATCACGATTGGAGTGCTGGCCAGGGTGGCTCTGATA-TAG
Ppy_CEACAM5 AAGTGCTCCTGTCCTCTCAGCTGCGGCCACTGTCGGCATCATGATTGGAGTGCTGGCCAGAGTGGCTCTGATA-TAG
Hsa_CEACAM5 AACTTCTCCTGGTCTCTCAGCTGGGGCCACTGTCGGCATCATGATTGGAGTGCTGGTTGGGGTTGCTCTGATA-TAG
Hsa_CEACAM7 AAGTTCACCTGACCTCTCAGCTGGGACCGCTGTCAGCATCATGATTGGAGTACTGGCTGGGATGGCTCTGATA-TAG
Ssc_CEACAMa AAGTACTTCTGGCGTCTCAGATGGTGGTATTGCTGGCATTGTGATTGGAGTCCTGGCTGGGGTAGCTCTCATAGCAGGC---CTGGTGTAA
Age_CEACAMa CACTGTCAGCATCATGACTGGAGTGCTGGCTGGGGTGGCTCTGATAGCAGCC---CTGGTGTAG
Cmo_CEACAMa TCTCAGCTGGGACCACTGTCAGTATCACGACTGGATTGCTGGCTGGCGTGGCTCTGATAGCAGCC---CTGATGTAG
 Hsa_CEACAM1 AAAT------GGCCTCTCACCTGGGGCCATTGCTGGCATTGTGATTGGAGTAGTGGCCCTGGTTGCTCTGATAGCAGTAGCCCTGGCATGT
B
Ssc_CEACAMa …..REDAGNYQCEASNPGNSSLSDPLRLDVKFDSIPGSTSGVSDGGIAGIVIGVLAGVALIAG LV
Supplementary Fig. 3
