# Supplementary material for: Coevolution of activating and inhibitory receptors within mammalian carcinoembryonic antigen families
Source: BMC Biol. 2010 Feb 4;8:12. doi: 10.1186/1741-7007-8-12 (PMC2832619; doi:10.1186/1741-7007-8-12)
Supplement: Additional file 4 — Sequence data. Nucleotide sequences from N domain exons as gene identifier for carcinoembryonic antigen related cell adhesion molecule (CEACAM) genes. [file 1741-7007-8-12-S4.DOC]

**N domain exon nucleotide sequences from mammalian CEACAM genes**

*Bos taurus* (cattle)

>Bta_CEACAM1^a^N

TCTCACTCTTAACTTTCTGGACCCCGCCCACCACTGCCCAGCTCACTATTGAAACGGTGCCCCCCCTTGCTGCAGAAGGTTCAGATGTTCTTCTGCTTGCCCACAACGTGACAAAGAATCCTCTAGGCTATGCCTGGTACAGAGGAGAAAGGGTAGACAACAGCCAGCTAATCGCATCATATAGGGTAGCCACTACCAAAGGGCCTGCACACAGTGGACGGGAGACATTATACCCCAACGGAACCCTGCTGATCCAGAACGTCACCCAGAAAGACACAGGATCCTACATGCTGCTCGTTACAAAGGATGATTTACAGACAGAAAGACAAACTGGACACCTCCACATACTCC

>Bta_CEACAM1^b^N

TCTCACTCTTAACTTTCTGGACCCCGCCCACCACTGCCCAGCTCACTATTGAAACGGTGCCCCCCCTTGCTGCAGAAGGGTCGGATGTTCTTCTACTTGCCCAAAACGTGACAAAGAGTCCTCTAGGCTATTCCTGGTACAGAGGAGAAAGGGTAGACAACACCCAGCTAATCGCATCATATAGGGTAGACACTAACGCAACTACCAAAGGGCCTGCACACAGCGGACGGGAGACACTTTACCCCAACGGAACCCTGCTGATCCAGAGCGTCACCCAGAAAGACACAGGATCCTACATGCTGCTCGTTACAAAGGATGATTTACAGACAGAAAGACCAACTGGACACCTCCACGTACTCC

>Bta_CEACAM16N1

CCGCGATCCTGAGTGCGGGGGCCGAGATCATTATCACCCCCGAGCCTGCCCAGCCAGCCGAGGGCGACAACGTCACTCTGGCTGTCCAAGGGCTTTCGGGGGAACTGCTGGCCTACAACTGGTATGCGGGGCCCACGCTCAGCCTGTCTTACCTGGTAGCCAGCTACATTGTAAGCACAGGCGATGAGACCCCTGGCCCAGCCCACACGGGGCGGGAGGCTGTGCGCCCCGACGGTGGCCTGGACATCCAGGGTGCCCTGCCTGGGCACTCGGGCACCTACATTCTGCAGACTCTCAACAGGCAATTTCAGACGGAGGTGGGCTACGGACACATGCAGGTCTATG

>Bta_CEACAM16N2

CGGCCACGGTCATCATGACCATCGTTCCGGTGCCCACCCGGCCAATGGAGGGCCAGGACGTGACACTGACCGTCCAGGGCTACCCCAAGGATCTGCTGGTCTATGCCTGGTACCGTGGGCCTGCCTCCGAGCCCAACCGGCTTCTCAGCCAACTTCCTTCCGGGAACTGGATCGCAGGCCCTGCGCACACAGGCCGGGAGGTGGGCTTCCCCAACTGCTCACTGCTGGTGCAGAAGCTGAACCTCACAGATGCCGGCCGCTACACCCTCAAGACCGTCACGCTGCAGGGCAAGACAGAGACACTGGAAGTGGAGCTGCAGGTGGCCC

>Bta_CEACAM18N

CCAGTCTGCTGGCCTGTGGGATCCGCCAGGCCTCCAGCCAAATCTACATCACCCCGGATTCACTCATCGGAGTGGAAAGATATTCGAGCTCACTGGCCATCGAGAACGCCCCTGAAGATGTTCAGGAATACAGCTGGCACCGAGGTGCAAATGACACTGAGGAAAATCTGATTATCAGCTACAACGCCACATCTCATTCCAGGCGGGATGGGCCCATGTACAGCGGCCGGGAAAGTGTGTCCATTAGAGGTACCCTGAGGATCTGGAGGTCACAGTTAAATGACACGGGGAACTACACAGTGAGGGTGGACACCATCAATGACACCCAGAGAGCAACTGGCTGGCTCGAGATTCTAG

>Bta_CEACAM19N

CCTCCATCCTGGCCCTCTGGGTCCCCCAAGGCTCCTGGGCAGCCCTGCGCATCCAGAAGATTCCGGAGCAGCCTCAAATGAACCAGGACCTTCTCCTGTCTGTCCAGGGCATCCCAAACACCTTCCAGGACTTCAGCTGGTACCTGGGGGAGGAGGCCAATGGCGGTACAATGTTATTCACCTACATCCCCAAGCTACTACGCCCCCAGAGGGATGGCAGTGCCATGCATCAGCGAGACATTGTTGGCTTCTCCAATGGCTCCATGCTGCTACGTCACGCCCAGCCCAGCGACAGCGGCACCTATCAGGTAGCTGTCACCATCAACCCTTCCTGGACCATGCGGGCCAAGACTGAGGTCCAGGTGGTCG

>Bta_CEACAM20N

CCTCACTTTTGACCGTGTGGAGCTTGACAGCTGCAGCCCAGATCTCCCGTGATGCTGTCATCCAAAGTGAGGAGGATATTGTTCCGTCTACATTTGGGGCCCCTTGGTTACCCCAGACTCATG

>Bta_CEACAM32N

TCTCGCTCTTATCCTTCTGGACCCTGCCCACCACTGCCCGCCTCACTGTTGACACGGTGCCCCCACTTGCTGCAGAAGGGTCGGTCGCTGTTTTTAACATCCTCGAAAAGGAAGGGTTGATCATCGGCTATGGCTGGTTCAGAGGGAACAGGATAGATCAAAGAGCTGCGATTGAAGCCTACCAAATAATTAACAATTCACACACACCTGGGCCTTCACACACCGGTCGAGAGACAATAAAACCCAATGGCTCCCTGGTAATCCAGTCCGTCAAGAAGCAGGACGCAGGAACCTACACCGTGATCACTGTCAAGGCTGATTTAACGAACGTTTCGGCTTCTGGACAGCTCCAAGTATACA

>Bta_CEACAM33N

TCTCACTCTTAACTTTCTGGACCCCGCCCACCACTGCCCAGCCCACGATTGAAACGATGCCCCCTCTTGCTGCAGAAGGGTCGGATGTTCTTCTACTTGCCCACAACGTGGCAAAGAATCCTCTAGGCTATGCCTGGTACAGAGGAGAAAGGGTAGACAACAGCCAGCTAATCGCATCATATAGGGTAGACACTAACGCAACTACCAAAGGGCCTGCACACAGCGGACGGGAGACACTTTACCCCAACGGAACCCTGCTGATCCAGAGCGTCACCCAGGAAGACACAGGCTCCTACACGCTGCTCGTTACAAACGATGATTTACGGACAGAAAGACAAACTGGACACCTCCACGTACACC

>Bta_CEACAM34N

TCTCCCTCTTAACTTTCTGGACCCCGCCCACCACTGCCCAGCCCACTATTGAAACAGTGCCCCTCCTTGCTGCAGAAGGGTCGGATGTTCTTCTGCTTGCCCACAACGTGACAAAGAATCCTCTAGGCTATGCCTGGTACAGAGGAGAAAGGGTAGACAACACCCAGCTAATTGGATCATGTAGAGTAGCCACTAACCTAACTACCAAAGGGCCTGCACACAGCGGATCGGAGACACTTTACCCCAACGGAACCCTGCTGATCCAGAGTGTCACCCAGGAAGACACAGGCTCCTACACGCTGCTCGTTACAAAGGATGATTTACAGACAGAAAGACAAACTGGACACCTCCACGTACACC

>Bta_CEACAM35N

TCTCCCTCTTAACTTTCTGGACCCCGCCCACCACTGCCCAGCTCACTATTGAAACGGTGCCCCCCCTTGCTGCAGAAGGGTCGGATGTTCTTCTACTTGCCCACAACGTGACAGAGAATCCTCTAGGCTATGCCTGGCACAGAGGAGACAGGATAGACAACAGCCAGCTAATTGCATCATATAGAATAGACACTAATGTAACTACCAAAGGGCCTGCACACAGCGGACAGGAGACACTGTACCCCAACGGAACCCTGCTGATCCAGAGCGTCACCCAGAAAGACACAGGATCCTACACGCTGCTCCTTACAAAGGATGATTTACAGACAGAAGGACAAACTGGACATCTCTGCATATATA

*Canis familiaris* (dog)

>Cfa_CEACAM1N

TCTCACTCTTAGCCTTCTGGAACCCGCCCACCACTGCCCAAGTCACTGTGGAGTCCGTGCCTCCCAATGCTGCTGAAGGGAAGGACGCTCTTCTGCGGGTCCTCAATCTGCCTGGGGATACAGCAAGCCTTACCTGGTTCAAAGGGGAAACTGTATTGCCGACCCATAAAATTCTATTATATGTAATAGACACCAAAATCACTACCCCGGGGCCTGCATACAGCGGCAGAGAGACAATATACCCCAACGGATCCCTGCTATTCCAGAACATCACCCTGAACGACACTGGATCCTACATCCTACAAATCATAAACCAAAAATTTGAAACTGCACTAGTACGTGGACAGCTCCAAGTATTCC

>Cfa_CEACAM16N1

CCACTTTTCTGAGCGTGGGGGCCGAGATCTCCATCACCCCCGAGCCCGCCCAGCCAGCTGAGGGGGACAACGTGACGCTGGCCGTCCACGGGCTTTTGGGGGAGCTGCTTGCCTACAACTGGTACGCGGGACCCACTCTCAGCCTGACTTACCTGGTGGCCAGCTACATTGTGAGCACAGGCGACGAGACCCCTGGCCCGGCCCACACAGGGCGGGAGGCTGTGCGCCCCGATGGCAGCCTGGACATCCAGGGCGTCCTGCCCCGCCACTCGGGCACCTACATCCTGCAGACTCTCAACAGGCAGCTGCAGACGGAGGTGGGCTACGGACACTTGCAGGTCTATG

>Cfa_CEACAM16N2

CGGCAGCAGTTGCCATGACAATTGTGCCTGTGCCGACCAAGCCGATGGAGGGCCAGGACGTGACACTGACCGTACAGGGCTACCCCAAGGACCTGCTGGTCTATGCCTGGTACCGCGGGCCTGCCTCCGAGCCCAACCGGCTGCTCAGCCAACTGCCGTCAGGGAACTGGATCGCAGGCCCCGCACACACAGGCCGGGAAGTGGGCTTCGCCAACTGCTCACTGCTGGTGCAGAAGCTGAACCTCACGGATGCTGGCCGCTACATGCTCAAGACCGTCACATTGCAGGGCAAGACAGAGACGCTGGAAGTGGAGCTGCAGGTGGCCC

>Cfa_CEACAM18N

CCAGTCTGCTGGCCTGTGGGATCCACCAAGCCTTTGGCCAAATGTTCATCAGCCCAGACTCACTTATAGGAGTCAAGGGATTTCGGACTGTCCTGGTCCTCGAGAATGCCACCCAAGATGCTCAGGAATACAGTTGGCACCGTGGTGCAGAGGACACTGTGGAAAATATGATTGTCAGCTACAAACCTCCCTTCAATTCCTGGCTATCTGGGCCTATGTTCAGCGGCCGGGAGAATGTGACCAGGTTGGGTGACCTGGTGATCAGGAGATCTGCATTTAGTGACACAGGGAACTACACTGTAAGGGTGGACACAGGCAATGAGACCCAGAGAGCAACTGGCTGGCTTGAAATTCAAG

>Cfa_CEACAM19N

CCTCAATCCTGGCCCTCTGGATCCCACAAGCTCTTGGGCTGCCCTACGCATCCAGAAGATTCCAGAGTATCCTCAAAAAGACCAGGACCTTCTCCTGTCTGTCCAGGGCATCCCAGGCAACTTTCAGGACTTCAACTGGTACCTGGGGGAGGAGACCAATGGTGGCACGATGTTATTCACCTACTTCCCCGATCTCCAGTGGCCCCAGAGGGACGGCAGTGCCATGGGACAGCATGACATCGTTGGCTTCCCCAATGGCTCCATGATGCTGCATCGTGTCCAGCCCACCGACAGTGGCACCTACCAGGTAGCTGTCAACATCAATCCTGCCTGGATCATGAGGGCCAAGACTGAGGTCCAGGTGGCCG

>Cfa_CEACAM20N

CCTCACTTTTGACCATGTGGAGTCTACCAGCTGCAGCCCAACTCACCCTTGATACCAACCCATTTACCACCACCCAAGGTGAGAAGGATGCTGTTCCATCTATGTCTGGGACCCCCTGGGCAACTCAGACTCATGGCAGATTCATAGATGTGGACACAGAAAGCAAAGCTATCCTTCTGATCCCTGCCTTCATACCTAAATCACCATTAGGATTCCATACTCGAA

>Cfa_CEACAM23N

TCTCACTCTTAATCTCCTGGAACCCGCTCACCACTGCCCAAGTCACTGTGGAATCCGTGCCTCCCAATGCTGCTGAAGGGAAGGACGCTCTTCTGCGGGTCCTCAATTTGCCTGGGGATACAGCAGGCCTTACCTGGTTCAGAGGGGAAATTGTAGCACCTGTCCATCAAATTGTATTATATGTAGTAGACACACGAGTAATTACCCCGGGGCCTGCACACAGTGGCAGAGAGATAATATACCCCAATGGATCCCTGCTGTTCCAGAACATCACCCTGAGCGACACTGGATCCTACATCCTACAAATCATAAACAGAAAATTTGAAACTGCACTAGTAACTGGAGAGCTCCGAGTATTCC

>Cfa_CEACAM24N

TCTCACTCTTAACCTCCTGGAACCCGCTCACCACTGCCCAAGTCACTGTGGAATCCGTGCCTCCCAATGCTGCTGAAGGGAAGGACGCTCTTCTGCGGGTCCTCAATCTGCCTGGGGATACAGCAGGCCTTACCTGGTTCAGAGGGGAAATTGTAGCACCTGTCCATCAAATTGTATTATATGTAGTAGACACACGAGTAATTGTCCCGGGGCCTGCATACAGTGGCAGAGAGATAATATACCCCAATGGATCCCTGCTGTTCCAGAATATCACCCTGAAAGACACTGGATCCTACATCCTACAAATCATAAACCAAAAATTTGAAACTGCACTAGTACCTGGACAGCTCCGAGTATTCC

>Cfa_CEACAM25N

TCTCACTCTTAACCTTCTGGAACCCGCCCACCACTGCCCAAGTCACTGTGGAGTCGGTGCCTCCCAATGCTGCTGAAGGGAAGGACGTTCTTCTGCGAGTCCTCAATCTGCCTGGGGATCTTCTAGGATATGCCTGGTTCAGAGGGAAAAGTGTAGAGACCAATAATCGTATTGTGTCATATGTGGTAAACACACAAGTGATTACCCCCGGGCATGCACACAGCGGCAGAGAGACAGCATACCCCAATGGATCCTTGCTGTTTCAGAACATCACCCTGAAGGACACGGGGTACTACACCCTACAAATCATTACAAATGATGTTCAAGTTGAACAAGTACGTGGACAGCTCCGAGTATTCC

>Cfa_CEACAM26N

TCTCTTTTAACTTTCTGGAACCCGCTCACCACTGCCCAAGTCACTGTGGAATCCGTGCCTCCCAATGCTGCTGAAGGGAAGGACGTTCTTCTGCGGGTCCACAATCTGCCTGGGGATACAGCAAGCCTTGCCTGGTTCAGAGGGGAAACTGTAGCACCTGTCCATCAAATTGTATTATATGTAGTAGACACACGAGTAATTACCCCGGGGCCTGCATACAGTGGCAGAGAGATAATATACCCCAGTGGATCCCTGCTGTTCCAGAACATCACCCTGAACGACACTGGATCCTACATCCTACAAATCATAAACAGAAAATTTGAAACTGCACTAGTACGTGGACAGCTCCGAGTATTCC

>Cfa_CEACAM27N

TCTCACTCTTAACCTTCCAGAACTCGCCCACCACTGCCCAAGTCCTGTGGAATCTGTGCCTATTGATGCTGCCGAAGGGAAGGATGTGCTTCTGCGTGTCCACAATCTGCCCGGGAATCTTCTAGGCTATGACTGGTTCAGAGAGGAAATTCTATCGTATCATATGTAGGAAACACACAAGTGATTACCCTGGGGCCTGCACATGGGGCAGAGAGACAGCCTACCCCAATAGATCCCTGCTGTTCCAGAACATCACCCTGAACGACATGGGGTATTACACCCTACAAATCATTAAGAAAAAATTTCAAATTGAACAAGTACTTGGACAGCTCCGTGTATTTC

>Cfa_CEACAM28N

TCTCACTCTTAGCCTTCTGGACCCCGCCCACCACTGCCCAAGTCACTGTGGAGTCCGTGCCTCCCAATGCTGCTGAAGGGAAGGATGCTCTTCTGCGGGTCCTCAATCTGCCTGGGGATACAGCAAGCCTTACCTGGTTCAAAGGGGAAACTGTATTGCCGACCCATAAAATTCTATTATATGTAATAGACACCAAAATCACTAACCCGGGGCCTGCATACAGCGGCAGAGAGACAATATACCCCAACGGATCCCTGCTGTTCCAGAACATCACCCTGAACGACACTGGATCCTACATCCTACAAATCATAAACCAAAAATTTGAAACTGCACTAATACGGGGACAGCTCCAAGTATTCC

>Cfa_CEACAM29N

TCTCACTCTTAATCTCCTGGAATCCGCCCACCACTGCCCAAGTCACTGTGGAGTCCGTGCCTCCCAATGCTGCTGAAGGGAAGGATGCTCTTCTGCGGGTCCTCAATCTGCCTGGGGATACAGCAGGCCTTACCTGGTTCAGAGGGGAAACTGTATTGCCGACCCATAAAATTCTATTATATGTAATAGACACCAAAGTCACTACCCCGGGGCCTGCATACAGCCACAGAGAGACAATATACCCCAATGGATCCCTGCTGTTCCAGAACATCACCGTGAATAATGCCGGATATTACATCCTACAAATCATAAACAGAAAATTTGAAACTGCACTAGTACCTGGACAGCTCCGAGTATTCC

>Cfa_CEACAM30N

TCTCACTCTTAGCCTTCTGGAACCCGCCCACCACTGCCCAAGTCACTGTGGAGTCTGTGCCTCCCAATGCTGCTGAAGGGAAGGATGCTCTTCTGCGGGTCCTCAATCTGCCTGGGGATACAGCAAGCCTTACCTGGTTCAGAGGGGAAACTGCATTGCCGACCCATAAAATTCTATTATATGTAATAGACACCAAAGTCACTACCCCGGGGCCTGCATACAGCGGCAGAGAGACAATATACCCCAACGGATCCCTGCTGTTCCAGAACATCACCCTGAATGATGCCGGATCCCACATCCTACAAATCATAAACCAAAAATTTGAAACTGCACTAATACGGGGACAGCTCCGAGTATTCC

Cavia porcellus (Guinea pig)

>Cpo_CEACAM1-like_1N

CATCACTGTTGACCTTCTGGAGCCCACCCACCACTGCTCAACTGACTATTGAATCGGTGCCTTTCGATGCTGTTGAAGGGGCAGATGTTCTTCTAGTTGCCCACAATTTGCCAGAGAACGTTTTAGGCTATTCATGGTTCAAGGGGAATACGACTGCTTCTGACAATATGATTATACGGTATGTGACAAACAATAATATCAATGTAACAGGGAAGGCATACAGCAATCGAGAGACAATATACCGCAATGGATCCCTGCTGATCCAGAACGTCACTCTACAGGACTCAGGATTCTACACCCTACGAATCACAGATGAAACTTTAGACACTAAAGACACATATGGACAGTTCCACGTACACC

>Cpo_CEACAM11-like_2N ITIM/ITSM

CATCACTTTTGGCCTTCTGGAGCCCACCCACCACTGCCCAACTGACTATTGAATCGGTGCCTTTCGATGCTGTTGAAGGGGCAGATGTTCTTCTACTTGCCCACAATTTGCCAGAGAACACTGTAGGCTATTCATGGTTCAAGGGGAATACGACTGCTCCTAACAGTATGATTATACGATATGTGACAACCAATAATGTCACTGTACCAGGGAAAGCATACAGCGGTCGAGAGACAATATACCGCAATGGATCCCTGCTGATCCAGAACGTCACTCTACAGGACTCAGGATTCTACACCCTACGAATCACAGATGAAACTTTAGACAATAAAGACACATATGGACAGTTCCACGTACACC

>Cpo_CEACAM1-like_3N ITIM/ITIM

CCTCACTTTTGACCTTCTGGAGCCCACCTACCACCGCCCAACCAGTTGTTCAAGCAATTCCTCCCAATGTTGTTGAAGGAGAAAATGTTCTTCTACAACTCATCCATGCGGCAACAAATCATTGGGGCTACAAGTGGTACAGAGGGACAATAGTTGCTTCCGACATGATGATTGCAACATTTTCCAAAGTCAGTGAACAGAATACTACAGGAAATGCATACACCGGTCAAGAAATAATATACTCCAATGGATCCCTGCTGATCCGGAACATCACCCGAGAGAACGCAACAAACTACATCGTGGAGGTTATACTAGATGATTTGATAGTTCTTCAGGCATCTGGAGAATTTCGTGTATATT

>Cpo_CEACAM1-like_4N

CCTCACTTTTGACCTTCTGGAGCCCATCTACCACCGCCCAACCAGCTATTGAATCAGTGCCTTACAATGTTCTTGAAGGGGGAAATGTTCTTCTACGACTCTACAATGTGACAGTGCAACCTTCTGGCTATAAGTGGTACAGTGGGGTAACAAGTACTCTGAACAAGATGATTGTAGCATATTCCAGAGCCACTAATGAGTCTACTACTGGGGATGCATACAGCGGTCGAGAGACAATATACCCCAATGGATCCCTGCTGATCCAGAATGTCACCCAAGATGACCCAACATTCTACACCATACAACTCATATTGGATAATGGTGTAACTCAAGAGTTATCCGGAGAGTTCCGTGTATACT

>Cpo_CEACAM1-like_5N pseudogene

CATCACTTTTGACCTTCTGGAGCCCCACTACCACTGCCCAACCAGCTATTGAAATACTGCCTGGTGATATTAATATAGGGGGAAATGTTCTTCTACGACTCTACAATGTGACAGTGGAACCTACAGAGTATAGGTGGTACAGTGGGGGATCGATTTCACCTGCCACATTGATTATAACATATTCCAGAGTCACTAATGAGTCTACTACAGGGGACTCATACAGTGGTCGAGAGACAATATACCCCAATGGATCCCTGCTGATCCAGAATGTCAACCGAGGCGACCAAGTAATCTACACCATACAAATCATGTCGCTAAATGGTGTGTATGCTGAGCTGTCTGCAGAGATCCGTATAACCT

>Cpo_CEACAM1-like_6N pseudogene

CCTCACTTTTGACCTTCTGGAGCCCACTCACCACTGCCCAACTAACTATTGAATTGGTGCCTTTTGATGCTGTTGAAGGGGGAGATGTTCTTCTACTCCTCCATAATTTGCCAGAGAACATTTTAGGCTATAAGTGGTACAAAGGGGAAAGGATTGACTCCAAAAGTGTGATTGTAATGTATGAGACAGCCAATAATGCCACTACATTAGGGCCTGCACACAGCAATCGAGACAGTATACCCCAATGGATCCCTTCTGATCCAGAGTCTCACCCACCGGGACTCAGGCTTCTACACCCTAGAAGCCATAAAGTCAACTTTGCTGAGTGAAGATGCATCTGGAGAGTTCT

>Cpo_CEACAM1-like_7N

TCTCTCTTTTGACCTTCTGGAGCCCACTCCACAATGCCCAAATGACTATTAAATCGGTGCCTTTTGATGCTGTTGAAGGGGCAGATGTTCTTCTACTTGTCCACAATTTGCCAGAGAACATTATAGGTTATAGGTGGTACAAAAGGGAAAAGGTTGATTCCAACAGTCACATTGCAGCATATTTGGTATCCACTAATGTCACTAGACCAGGGCCTGCATACAGTCATCGAGAGACTATATATCCCAATGGCTCCCTGCTGATCCAGAAGGTCACCCAACAGGACACAGGATTCTACACCCTACAAGCCATAAAGTCTACTTTGCAGAATGAAGAAGCATCTGGACAGTTCCATGTACACC

>Cpo_CEACAM1-like_8N pseudogene

TCTCTCTGTGCCCTTCTGGAGCCCACCCACCACTGCTCAACTGACTATTGAATTGGTGCCTTTCGATGCTGTTGAAGGGGCAGGTGTTCTAGTTGTCCACAATTTTTCAGGGAAGGTTTACATCCTTGTGTGGCTCAAAGGGAATACAAATGCTTACAAAAGTATGATTGTAGGATATTTCACATTCCTTAGTCTCATTCAAAAAGGGAATTCATACAGTGGTCAAGAGTCAACATACGCCAATGGTCCTTGCTGGTGCAGAACGTCACTCAACAGGGCACAGGATCCTACATCCTAAAGTTCTCAACAACAATTTGGAATGCTGAATACACATATGGACAGTCCTGTGTAC

>Cpo_CEACAM1-like_9N

CGTCAATTTTGACCTTGTGGAGCCCACCCACCACTGCCCAAATGACTATTGAATCGGTGCCTTTTGATGCTGTTGAAGGGGCAGATGTTCTTCTACTTGTCCACAATTTGCCAAAGTACATTTCAACCTATAAGTGGTACAAAGGGGAAACGACTGCAACCAAAAGTATGATTATACAATATTCGACAATCATGAAGAGACATACTACAGGGGATGCACACAGCAATCGAGAGACGATATACCCCAATGGATCCCTGCTGATCAAGAATGTCACCCGAGAGGACACAGGATTCTACACCTTCAGCATAAATCGAGTTTTGAAACCTAAAACTGCATCTGGACAGTTCCATGTACACA

>Cpo_CEACAM1-like_10N pseudogene

CCTCACTTTTGACCTTCTGGAGCCCACCTACCACTGCCCAATCAGGTATTGAAATACTGCCTGGGACTGTTAATATAGGGGGAAATGTTCTTCTACGACTCTACAATTTGACAGTGAAAACTTCAGAGTATAAGTGTTACAGTGGGGGAACGACTTCACCCACCACATTGATTATAACATATTCCAGAGCCACGAATGAGTTTACTACAGGGGAAGCATACAGAGTCGAGAGACAATATACCCCAATGGATCCCTGGTGATCCTGAATGTCACCGGAGGTGACCAATAATCTACAGCATACAACTCATATTTTAAAATGATGTATCTCATGAGGTGACCGAAGAGTTCCGCATAAACT

>Cpo_CEACAM1-like_11N pseudogene

TCTCTTTTTTGATCTTCTGGAGCCCACCCCCAGCTGACCAACAGACTATTGAATCAGTGCCTTTTGATGCTGTTGAAGGGGCAGATGTTCTTCTACTTGTCCACAATTTGCCAGAGAACAGTATAGATGGTACTCCTGGCAGTGTGATTGTAGCACATGGTATGCATATAGCAGTCGAAAGAAAATATGCCCAAATGGATCTGTGCTGATTCAGAACCTCACCCAACAGGACACAGGATTCTCCACCCACAAGTCACAGAGGTAAACATGGAGACTGAAGATGCACCTGGACAGTTCCGTGTACATG

>Cpo_CEACAM1-like_12N pseudogene

CTATCTGTCCACCATGTTATCCCAGGAGTCCAGGGCCAACTGACTATTGAATTGGTGCCTTTCGATGCTGTTGAAGGGGCAGATGTTCTTCTACTTGTCCACAATTTGCCAGAGAACATTTTAGGCTATAGGTGGTACAAAGGGAAAATAACTACTCCAGCAGTGTGATTGTAGCATATGGGGTAGCTGGTAGTGTCACCACATAGGGGATGCATACAGCAGTCAAAATACAGTTTACCCAAATGGATCTGTGCTGAGCCAGAGCCTCACTCAACAGGACACAGGATTCTCTACCCATAAGCCACAGATAAAAATCTGGAGACTGAAGACACATGTGACAGTTCATGTACA

>Cpo_CEACAM1-like_13N pseudogene

CTCTCTTTTAACTTTGTGGATCCCACCCACCACTGCCCAACTGACTATTGAATTGGTGCCTTTCGATGCTGTTGAAGCGGCAGATGTTCTTCTTCTCATCCACAATTTGCCAGGGAAATTTTACAGCTTTACTGAAAGGGAATACAAATGCTGACAACAGTATGATTGTGGCATATTTCACATTCCTTAGTCTCACTCAAAAAGGGAATTTGTACACCAGTCAACAGTCAACATATGCCAGTGGATCCTTGCTGGTCCAGAATGTCACCCAAGAAGACACAGGATTCTACACGCTATAAAGATCTCATGGACAATTTGGAACTCGGAATACACATCTGAACAGTTCCGTGTACAC

>Cpo_CEACAM1-like_14N pseudogene

TCTCTCTTTTGATCTTCTGGAGCCCACCCCCAGCTGACCAACAGACTATTGAATCAGTGCCTTTTGATGCTGTTGAAGGGGCAGATGTTCTTCTACTTGTCCACAATTTGCCAGAGAACATTATAGATGGTACAAAGGGAAAATAACTACTCCTGGCAGTGTGATTGTAGCACATGGTATGCATATAGCAGTCGAAAGAAAATATGCCCAAATGGATCTGTGCTGATCCAGAACCTCACCCAACAGGACACAGGATTCTCCACCCACAAGTCACAGAGGTAAACATGGAGACTGAAGATGCACCTGGACAGTTCCGTGTACACG

>Cpo_CEACAM16N1

CTGCATTCCTGAGTGCAGCGGCCGAGATCACCATCACCCCCGAGCCGGCCCAGCTGGCCGAGGGGGACAACGTCACGCTGGTGGTCCGCGGGCTCTCGGGGGAAGAGCTGGCCTACAGCTGGTACGCGGGGCCCACACTCAGCCTGTCCTACCTGGTGGCCAGCTACATCGTGAGCACGGGCGACGAGACCCCTGGCCCGGCCCACACGGGGCGGGAGGCCGTGCGCCCCGACGGCGGCCTGGACATCCAGGGCCTCCTGCCCGGGCATTCGGGCACCTACATCCTGCAGACCCTCAACAGGCAGCTGCAGACCGAGGTGGGCTACGGACACGTGCAGGTCTATG

*Dasypus novemcinctus* (armadillo)

>Dno_CEACAM1N

TCTCACTTTTGATCTTCTGGAGCCGGCCCATCACAGCCCAGCTCACGATTGAATCTGTGCCAGCAGATGCTGCTGAAGGCAGCGATGTTCTTCTGATTTTCCACAATCAGCCAGAGAATCCTCAAGGCTACAACTGGAACAAAGGGGACACAGTGGACCCCAACTATAACATTTTAGCACATGTAACAGACACTCAACAAACAACCCCAGGACCTGCCCACAGTGGTCGAGAGACCTTATACCCCAATGGATCCCTGCTGCTCCAGAAGGTCACCCAGAGTGACTCAGGAATCTACACCCTACAAGTCGTAAAGAGAAACCTACAGAGTGACTTAGCCTCTGGACAGATCTGCATGTACC

>Dno_CEACAM16N1

CCACGTTCTTGGGGGCCGCGGCGGAAATTTCCATCACCCCCGAGCCCGCCCAGCCAGCCGAGGGAGACAACCTCACCCTGGTGGCGCGCGGGCTCTCGGGGGAGCTGCTCGCCTACAACTGGTATGCGGGGCCCGCCCTCAGCCTGGCCTACCTGGTGGCCAGCTATATTGTGAGCACAGGCGACGAGACCCCCGGGCCGGCCCACACGGGGCGGGAGGCCGTGGGCCCCGACGGCAGCCTGGAGATCCAGGGCGTCCTGCCCAGCCACTCGGGCACCTACATCCTGCAGACCCTCAACAGGCAATTTCAGACCGAGGTGGGCTACGGACACGTGCAGGTCTATG

>Dno_CEACAM16N2

CTGCGGCAGTTGCCATGACGATTGTGCCTGTGCCGACCAAGCCAATGGAGGGCCAGGACGTGACACTGACCGTGCTGGGCTACCCCAAAGACCTGCTGGTCTACGCCTGGTACCGCGGGCCTGCCTCCGAGCCCAACCGGCTGCTCAGCCAGCTGCCGTCCGGGAACTGGATCGCCGGCCCCGCGCACACAGGCCGGGAGGTGGGCTTCCCCAACTGCTCACTGCTCGTGCAGAAGCTGAACCTCACCGACGCGGGCCGCTACACGCTCAAGACCGTCACGCTGCAGGGCAAGACGGAGACGCTGGAGGTGGAGATGCAAGTGGCCC

>Dno_CEACAM18N

CCGGCCTGCTGGCTTGTGGGAGCAGCCAGGCCTCCAGCCAACTCTCCATCAGCCCAGGCTCGCTCTTAGGACTCCTGGGAGAGGAGACCAGCCTGATGCTCGAGAATGCCCCCGAGGATGCTCTGGAATACAGCTGGCACCGGGGCCCAGACGACAAAGCAGAAAACATGATTATCAGCTACAGCCCTGCCTCCGCTTCCTGGAAGACCGGGCCTAAGTACACTGATCGGGAAAATGTGACCCGCATAGGCAGCCTGGTCATCCGCAAGACTGCACTGAATGACACGGGGGACTATACTGTGAGTGTCAAGGCCAGCAACGACACCCAGAAGGCAACCGGCTGGCTCGAGGTTCGAG

>Dno_CEACAM1-like_1N

TCTCACTTTTGATCTTCTGGAGCCGGCCCATCACAGCCCAGCTCACGATTGAATCTGTGCCAGCAGATGCTGCTGAAGGCAGCGATGTTCTTCTGCGTGTCCACAATCAGCCAGAGAATCCTTATGGCTACAACTGGTACAAAGGGGACAAGTTGGACCCCAAGTCTAACATTTTAGCATATGTAATAGACACTCAACTAACAACCCCAGGGTCTGCCCACAGTGGTCGAGAGACCATATACCCCAGTGGATCCCTGCAGCTCCAGAACGTCACCCAGAATGACTCAGGAATCTACACCCTACAAGTCATAAAGAGAAACCTTCAGGGTGATGCAGCTTCTGACAGAGCCGCGTGTACC

>Dno_CEACAM1-like_2N

TCTCACTTTTGATCTTCTGGAGCCGGCCCTTCACAGCCCAGCTCACGATTGAATCTGTGCCAGCAGATGCTGCTGAAGGCAGCGATGTTCTTCTGCTTGTCCACATTCAGCCAGAGAATCCTTATGGCTACAACTGGCACAAAGGGGACAAGGTGGACCCCAACTATAACATTTTAGCATATGTAACAGACACTCAACAAACAACCCCAGGACCTGCCCACAGTGGTCGAGAGACCATATACCCCAGTGGATCCCTGCAGCTCCAGTAGGTCACCCAGAGTGACTCAGGAATCTACATCCTACAAGTCATAAAGAGAAACCTACAGAGTGACTTAGCCTCTGGTCAGATCCGCGTATACT

>Dno_CEACAM1-like_3N

TCTCACTTTTGATCTTCTGGAGCCCGCCCATCACAGCCCAGCTCACAATTGAATCTGTGCCAGCAGATGCTGCTGAAGGCAGCGATGTTCTTCTGCGTGTCCACAATCAGCCAGAGAATTCTTATGGCTACAACTGGTGCAAAGGGGACAAGGTGAACCCAGTCATAATATTTTAGCATATATAATAGACACTCAACAAACAAGCGCAGGACCTGCCCACAGTGGTCGAGAGACCATATACCCCAATGGATCCCTGCAGCTCCAGAAGGTCACCCAGAGTGACTCAGGAATCTACACCCTACAAGTCACAGACAGAAACTTGAAGGTTCATAGAGCCTCTGGACAGATCCGCGTGTACC

>Dno_CEACAM1-like_4N

TCTCACTTTTGATCTTCTGGAGCCGGCCCATCACAGCCCAGCTCACGATTGAATCTGTGCCAGCAGGTGCTGCTGAAGGCAGCGATGTTCTTCTGCGTGTCCACAATCAGCCAGAGAATCCTCAAGGCTACAGCTGGAACAAAGGGGACAAGGTGGACCCCAACCATAACATTTTATCGTATGTAATAGACACTGAACAAACAACCCCAGGAACTGCCCACAGTGGTCGAGAGACCATATACCCCAATGGATCCCTGCTGCTCCAGAAGGTCACCCAGAGTGACTCAGGAATCTACACCTTAATAGTCATAAAAAGAAACTTTCAGGGTGATTTAGCCTCTGGACAGATCCGCGTGTACT

>Dno_CEACAM1-like_5N

TCTCACTTTTGATCTTCTGGAGCCGGCCCATCACAGCCCAGCTCACGATTGAATCTGTGCCAGCAGAGGCTGCTGAAGGCAGCGATGTTCGTCTGTGTGTCCACAATCAGCCAGAGGATCCTCGAGCCTACAACTGGCACAAAGGGGACACGGTGGAAAAGATCCATAAAATTTTAACATATGACACAGACACTCAACAAGCAATCCCAGGACCTGCCCACAGTGGTCGAGAGACCATGTACCCCAATGGATCCCTGCAGCTCCAGAAGGTCACCCAGAGGGACTCAGGAATCTACACCCTACAAGTCACAGACAGAAACTTTAAGGTTCATAGAGCCTCTGGACAGATCCGCGTGTACC

>Dno_CEACAM1-like_6N

CCTCACTTTTGATCTTCTCGAACCGGCCCATCACAGCCCAGCTCACGATTGAACCTGTGCCAGCAGATGCTGCTGAAGGCAGCGATGTTCTTCTGCTTGTCCACAGTCAGCTAGAGAATCCTCGAGCCTACAACTGGCACAAAGGGGAGAAGGAGGGCCCCGACCATATCATTTTATCACTTGTAACAGACACTCAACAAGCAACCCCAGGACCTGCCCACAGTGGTCGAGAGACCTTATACCCCAATGGATCCCTGCAGCTCCAGAATGTCAACCAGAACAACTCAGGAATCTACATGCTACAAGCCATAGATGGAAACTTTAAGGTTCATAGAGCCTCTGGACAGATCCGCGTGTACC

>Dno_CEACAM1-like_7N

TCTCACTTTTGATCTTCTGGAGCCCGCCCCTCACAGCCCAGCTCACGATTGAATCTGTGCCAGCAGATGCTGCTGAAGGCAGCGATGTTCTTCTGATTGTCCACAATCAGCCAAAGGATCGTCAAGCCTACCACTGGTTCAAAGGGGAGAGAGTGGACGCCAGCCAAAAAATCTTATCACTTAAAACAAACCCTCAACAAACAACCCTAGGACCTGCTCACAGCGGTCGAGAGACCATAGACCCTAATGGATCCCTGCTGATCAAGAATGTCACCCAGAATGACTCAGGAATCTATCATCTACAAATCATAGATACAAACTTGGATAGTCATAGCGCCTCTGGACAGATCTGTGTGTACT

>Dno_CEACAM1-like_8N incomplete

TCTCACTTTTGATCTTCTGGAGCCGGCCCATCACAGCCCAGCTCACGATTGAATCTGTGCCAGCAGATGCTGCTGAAGGCAGCGATGTTCTTCTGTGTGTCCACAATCAGCCAGAGGATCCTCGAGCCTACCACTGGTACAAAGGGGAGAGAGAGAACACCAGCCAAAAAATTTTATCACTTATAACAAACACTCAACAAATAACCCAAGGACCTGCCCACAGTGGTCGAGAGACTATATACCCTAATGGATCCCTGCTGATCAAGAATGTCACCCAGAATGACTCAGGAATCTATCATCTACGAATCATAGATACAAACTTTGATAGTCATAGTGCCTC

>Dno_CEACAM1-like_9N incomplete

CCAGCTCACGATTGAATCTGCGTACGAGACGAGGAGGTGCTGCTGAAGGCAGCGATGTTCTTCTGCATGTCCACAATCAGCCAGAGAATCCTTATGGCTACAGCTGGAACAAAGGGGACAAGGTGGACCCCAACTATAACATTTTAGCGTATGTAATAGACACTCAACAAACAACCCCAGGACCTGCCCACAGTGGTCGAGAGACCATATACCCCAATGGATCCCTGCAGCTCCAGAAGGTCACCCAGAGTGACTCAGGAATCTACATCCTACTAGTCATAAAGAGAAACCTTCAGGGTGATGCAGCCTCTGGACAGATCCGTGTGTACC

*Equus caballus* (horse)

>Eca_CEACAM1N

TCTTACTCTCAACCTTCTGGAACCCACCCACCACTGCCCAACTCACTATTGAATCGATGCCAACCAATGTTGCTGAAGGGAAGGATGTTATTTTCCTTGTCCGTAATCTGCCTGGGAATCTTGCAGCCTATGGCTGGTACAAAGGGGACAAAGTAGATCACCATCAACAAATTGCATCATATAAGATAGGCCCAGTAGAAATTACCCCAGGGCCTGTATACAGTGGTCGAGAGACAATATACTCTAATGGATCCCTGCTGTTCCGGAATGTCACCCAGGAGGACACAGGATACTACACCCTACAAGTCATAAATACAAGTTATCAAGGTGAAGTAGGAACTGGACAGCTCCGCGTATACC

>Eca_CEACAM16N1

CCACGTTCCTGAGCGCAGGGGCCGAGATCTCCATCACCCCCCAGCCCGCCAAGCCCGCCGAGGGCGACAACGTCACCCTGGCTGTCCAGGGGCTCGCGGGGGAGCTGCTTGCTTACAACTGGTACGCGGGGCCCACGCTCAGCCTGACTTACCTGGTGGTCAGCTACATCGTCAGCACGGGTGACGAGACCCCCGGCCCGGCCCACTCGGGACGGGAGGCTGTGCGCCCCGACGGCAGCCTGGACATCTGGGGCGTCGTGCCCGGGCACTCGGGCACCTACATCCTGCAGACTCTCAACAGGCAGTTCCAGACGGAGGTGGGCTACGGACACCTGCAGGTCTATG

>Eca_CEACAM19N

CCTCCATCTTGGTCCTCTGGGCCCCCCAAGGCTCCTGGGCTGCCCTCCACATCCAGAAGATTCCAGAGCACCCTCAAAAGAACCAGGATCTTCTCCTGTCTGTCCAGGGCGTCCCAGAAACCTTCCAGGACTTCAACTGGTACCTGGGGGAGGACGCCTACGGCGGCACGAGGCTCTTCACCTACATCCCGGGGCTGCAGCGGCCCCAGAGGGACGGCAGCGCCATGGGGCAACGAGACATCGTCGGCTTCCCCAACGGCTCCATGCTGCTGCGTCGCACCCAGCCCGGTGACAGCGGCACCTACCAGGTCGCTGTCACCGTCAACCCCGCCTGGACCATGCGGGCCAAGACTGAGGTTCAGGTGGCCG

>Eca_CEACAM41N

TCTCACTCTCAACCTTTTGGAACACACTCACCACTGCCCAACTCACTATTGAATCAGTGCCGACCAATGCTGCTGAAGGAAAGGATGTTCTTCTGCTTGTCCACAATCTGCCTGGGAATCTTGCCGCCTATGGCTGGTACAAAGGGGACAGAGTAGATCCCGATCAACAAATTGCATTGCATGTGATAGAGCGACTAGAAATTATCCCAGGGCCTCTATACAGTGGTCGGGAGAGAATGTACCCCAGTGGATCCCTGCTGTTACAGAAGGTCACCCAGGAGGACACAGGATACTATACCCTACAAGTCATAAATAAAAATTTTCACAGTGAAGTAGGAACTGGACAACTCTGCATATACA

>Eca_CEACAM42N

TCTCACTATTAACCTTCTGGAACCCACCCACCACTGCCCAACTCACTATTGAATCGGTGCCGACAGATGCTCTTGAAGGAACGGATGTTCTTCTACTTGTCCACAATCTGCCTGAGAATCTTTTAGGCTATGTCTGGCTGAAAGGGGAAGGAATAGATCCCGATCAACAAATTGTAATATATGTGATAGAAACACAAGAAAATACCCCATGGCTTCTATATAGTGGTCGAGAGACAACATACCACAATGGATCTCTGCTGTTCCAGAACGTCACCCGGAATGACACAGGATACTACACCCTACAAGCCATAGACCAAGATTTTAACAATGCAGTGGGAACTGTACAGCTCCGCGTATACC

>Eca_CEACAM43N

TCTCACTCTTAACCTGGATCCTGCCCACCACTGCCCAACTCACTATTGAATCGGTGCCGTCCAATGCTGTCCAAGGGAAGGATGTTCTTTTGCTTGTCCACAATCTGCCTGGGAATCTTGCAGCCTATGGCTGGTACAAAGGGGACAGAGTAGATCCCCATCAAGAAATTGTGTCCTATGTAATAAACACACTACAAACTACCCCAGGGCCTCTATACAGTGGTCGAGAGACAATATACTACAATGGATCCTTGCTGTTCCAGAATGTCACCCAGGAGGACACAGGATACTACACCCTACAAGTCTTCAAGGCAAATTTCCAAACTGAAGTAGGAATGGGACAGCTCCGCTTATACC

>Eca_CEACAM44N

TCTTACTCTCAACCATCTGGAACCCACCCACCACTGCTGGATTCAACATTGAATCAGTGCCATCCAAAGCTGTTGTAGGGACCGATGTTATTCTCCTTGTCACCAATCTGCCTCAAAATCTTTCTGCCTATATGTGGTATAAAGGTGACAGAGTAGAACCCAAACTTCACATTCTATCATATGCAATACATACATCAAAAATTACTTCAGGGCCTGGATACAATGGTCGACAGAAAATATACGCTGATGGATCCTTGCTGTTCCAGAATGCCACCCAGGAGGACACAGGATATTACACCCTAGAAGTTGTAAAAAGAAATTTCCTAATTGATATGGGACTTGGACAGCTCCACATATACA

>Eca_CEACAM45N

TCTCTCTCACAACTTTCTGGAACCAGCCCACTGCCGCTCAACTCACTATTGAATCGGTGCCGACCAATGCTGTCCAAGGGCAGGATGTTCTTCTGCTTGTCCACCATCCGCCTGGGGATCTGGCAGCCTATGTCTGGTACAAAGGGGCCAGAGTAGATCCCAACCAACACATTGCATTGTATGCGATAGGGCAACAAGAAATTATCCCAGGGCCTCTATACAGTGGTCGAGAGACAATATACTCCAATGGATCCTTGCTGTTCCAGAATGTCACCCAGGAGAACACAGGATACTACACTCTACAAGTCATAAAGGCAAATTTCCAAACTGAAGTAGGACTTGGACACCTGCAAGTATACC

>Eca_CEACAM46N

TCCTGCTCCTAACCTTCTGGAACCTGCACATCGCTGCTCAACTCACCGTTGAATCAGTCCCACCCAATGGTATCCAAGGCAAGGATATTCTTCTCCTTGTTTCCAATCTGCCTGCCAATCGTTTAGGCTATGTTTGGTACAAAGGGGACAGAGTAGAGCCCAAACTTCAAATAGTATCATATATAATAAACACATCAGAAATTACCCCAGGGACTGTGTACAGTGGTCGAGAGAAAATCTATGCTGATGGATCTCTGCTGTTCCAGAACGCCACCCCGGGGGACACAGGATATTACACCCTTCAAGTCATAAAGAGAACTTTGCTAACTGAAGTAGGAACTGGACAGCTCCACGTATATA

>Eca_CEACAM47N

TCTCACTCTTAAGCTTCTGGAACCTGCCAACCACTGCCAAAATCACTGTTGAATCGTTGCCGCCCAATGCTGCTGAAGGACAGAATGTTATTCTAGTTGTCCAAAATCTGCCCAGGAATCTTCTAGGCTATAACTGGTTTAAAGGGAACAGACCAAGTCCCAAAACCGAAATTGCACATTATGATATAGACAGAAAAACATTGATCCCAGGGCTTGCATTCACTGGCCGAGAGGCAATATGCCCCTATGGATCCCTGCTGTTAGCGAATGTCACCATGGAGTACGCAGGAAACTACACGGTGTTTGTCATAAAGAGACGTCTGCAATATGAAGTAGCAATTGCACAGCTCCACGTACACA

>Eca_CEACAM48N

TCTCAATCTTAACCTTCTGGAACTCGCTCACCACTGCCAAAGTCACTATTGTATCGGTGCCGCCCAAAGTTACTCCAGGAGAGAATGTTACTCTAGTTGTCCAAAATCTACCTAGGAATGTTATAGGCTATACCTGGTACAAAGGCTATGGACCAGCTCGCAAACAAAAAATTGCATATTATGATACAAACAGAAAAGTAATGACCCCAGGGCCTGCATTCACTGGTCGAGAGACAGTATACCCCAATGGATCCATGCTGTTTCAGAAAGTCACCGTGGAGTACACAGGAAACTACACGGTGTTTGTCATAAAGAGACGTCTGCGATATGAAGTAGCAATTGGACAGCTCCACGTATACTGGA

>Eca_CEACAM49N

TCTCAATCTTAGGCTTCTGGAACCTGCCCGCCACTGTCCAGTTCACTATTGAGTCGGTGCCGAACAATGTTACTGAAGGAAAGGATGTTCTTCTACTTGTCCACAATCTGACTGGGAATATTCTAGGCTATATGTGGTTCAAAGGGAATGGAGCACGTCCACATAAACAAATTAAGTTTTATGATGTAGACACAAAAGCATTTTCCACAGGGCCTCTAGCCACAGGTCGAGAGACAATGTACCCCAATGGATCCCTGCTGTTCCAGAATGTCACGACGGAGTACGCAGGAAACTACACACTACTTGTCCTAAAAAGATCCTTGATATATGAAGTAGGAACTGGACAAGTCCATGTATACA

>Eca_CEACAM50N

TCTCACTATTAACCTTCTGGAACCCACCCACCACTGCCCAACTCACTATTGAATCGGTGCCGACAGATGCTCTTGAAGGAACGGATGTTCTTCTACTTGTCCACAATCTGCCTGAGAATCTTTTAGGCTATGTCTGGCTGAAAGGGGAAGGAATAGATCCCGATCAACAAATTGTAATATATGTGATAGAAACACAAGAAAATACCCCATGGCTTCTATATAGTGGTCGAGAGACAACATACCACAATGGATCTCTGCTGTTCCAGAACGTCACCCGGAGTGACACAGGATACTACACCCTACAAGCCATAGACCAAGATTTTAACAATGCAGTGGGAACTGTACAGCTCCGCGTATACC

>Eca_CEACAM51N

CCTCACTCATAAGCTTCTGGAACCCGCCCACCACTGCCCAACTCACTATTGAATCGGTGCCGACCGATGCTGCTGAAGGGAAGGAAGTTCTTCTAGTTGTACACAATCTGCCTGGGAATCTCCTAGGCTATGGCTGGTTCAAAGGGGAAATAGATTCCAATCAACAAATTGCATCATATGTGATAGACACACAAGAAATGACACCAGGGCCTGTAAACAATGGTCAAGAGATAATATTCCCCAATGGATCACTGCTGTTCCAGAATGTCACCCAGGAGGACACAGGATACTACACCCTACCAGTCATAAAGATAAATTTTCAAAGTGAACTAGGAACTGGACAGCTCCACGTATACC

*Erinaceus europaeus* (Western European hedgehog)

>Eeu_CEACAM1-like_1N

TTTCTCTCATAGCCTTCCGGATACTGCCCACCACCACCCAGCTGACTGTGGAATCAGTGCCGGCCAATGCTGCTGAATGGGAAGATGTCTTCCTCCTTGTCTACAATATGTCAGGGAATCCTACAGGTTTCGGCTGGTACAAAGGGGAACACGTGGATCCAGACCAGCAAATTATAGGATATGAAACAGGTACACTTACAATGACCAATGGGCCTGCACATAGCGGTCGAGAGACATTATACACCAATGCGTCCCTGCTGTTCTGGAATGTTACCCAGGGTGACACAGGATACTACACGCTACTCATCATAAAAAGAGGTTTTCAAACTGAAAAAGTCACTGGGCAGCTACGTGTACACA

>Eeu_CEACAM1-like_2N

CTGACTGTGGAGTCTGTGCCGGCCAACACGACCGCCCAGCTGACTGTGGAGTCAGCGCCGGCCAATGCTGCTGAAGGAGAAGATGTTCTCCTCCTTGTCCACAATTGGCCTGAGAACACTATAGGCTTCAGCTGGTTCAAAGGGGAAAGTGTGGAGTCAGCCCATCAGATTGTAGGATATGAAATAGCCACACAACAAACGACCACTGGGCCTGCACACAGCGGCCGAGAGACACTATACACCAATGGATCCCTGCTGTTCCAGAATGTCAGAAAGAATGACACAGGCCTCTACACGCTACATGTCATTAAGAGTAGTTTGCAAATTGAAACAGTCATTGGGCAGCTTCGTGTTTGTG

>Eeu_CEACAM1-like_3N pseudogene

TCTCACTCTTAACCATCTGGATGTTGTCCACTACTGCCGAGATGACTATTGAAATAAAGTACCACCCAATCCTGTGGAAGGCAAAGATGTTGTTCTACTTGTCCACAATGTGCCTAGTGATGTTTATATCTATTACTGGTACACGGGATTAAGTTTGTACTCAAGCCGCTTAATTGCAACATATGAAACTGAGAGATAAAAGTTACCCAGGGCCTGCATACAATGGCCGACAGACAGTGTATCAAAATGGATCCCTGCTGATCCAGAGGGTCACCCAGAAGGACTCAGGACTCTACACCTTTCGACTTGTGAAGAAATTTTTTCAAGCAGAAGATGTAACAGTACAGCTCCAAGTAAACT

>Eeu_CEACAM1-like_4N

TCTCACTGTTGATCATTTGGATGTCATCCACTACTGCTAATATGACTTCTGAAGCAAAGCCACACAGAGTTGTCGAAGGGGATGATGTTTTACTACTTGCCCACAACCTGCCTAGTAATGTTCTGTTCCAATTTTGGTACACTGGAATACAGGCACTCAACTGCCATCTAATTGGAGTATATATAGAATACATGAGAAGAATTTACCAAGGGCCTGCATACACTGGTAAGGAGAAAATATATCCCAATGGATCCATGCTGATCCAGAGTGCCACACAGTTATCATGCAGGATACTACACGTTCAAAGTGATGACAAGACCGTTTCATTCAGAAATAGTAAGAATATGGATCCAAGTATACT

>Eeu_CEACAM1-like_5N

CTCACTCTTAACCATCTGGATGTCATCTGCTGCTGCTGAGATGACCATTGAAGTGACACCACCCAATCCTGTTGAGGGAAATGATGTGAATATAATTGTCCGCAATCTGCCTATTGACACAACTGTCCACTTTTGGTACATCGGAAGATCTTCACTCTTCAGACAGCTGATCGTGGCATATGAAAAAGATACAAAAAAAAACTTCCCAGGGCCTGCATTTACTGGCCGGGAGACAATCAGCCATAGTAGTTCTCTGCTGATCAGAAACATCACCCGGAAGCACACAGGATACTACATCTTCCAGGTCATGAAGCCATTTTATCAAACAGAAGAGGTACTCACATGGCTGCGAGTACACTGT

>Eeu_CEACAM1-like_6N

CTTCACTCTTAGCCATCTGGATGTCATTCTCTACTGCCCAGATCACTATGGAAGTAGTACCACCCAATCCTATCGAAGGAATGGATGTTCTTCTACTTGTCCACAACCTGACATGGGGTGATTCAGTCAAATTTTGGTATGTCAGTGAAAGTTTGAAATCAGTTCCTCAACTTGTTGTATTTGTAAACCTCAAGAATAAAAGCTACCCAGGGCCTGCATATAATGATCGACAGACAATCTATCACAATGGCTCCATGCTGATCAGGAATATCAACCGAAAGGACACAGGATTTTACAGCTTCCAAGTCTTAAGAAATTTTTCAGAAGAACAACAAATAACTATGCTGGTCCCAGTGTACT

>Eeu_CEACAM1-like_7N

CTGACAGTGAAGTCAGTGCCGGCCAACAGGACCGCCCCGCTGACTGTGGAGTCAGCGCCGGCCAATGCTGCTGAAGGAGAAGATGTTCTCCTCCTCGTCCACAATCGGCCTGAGAACACTATAGGCTTCAGCTGGTTCAAAGGGGAAAGTGTGGAGTCAGCCCATCAGATTGCAGGATATGAAATAGCCACACAACAAACGACCACTGGGCCTGCACACAGCGGCCGAGAGACACTATACACCAATGGATCCCTGCTGTTCCAGAATGTCAGAAAGAATGACACAGGCTTCTACACGCTACATGTCATTAAGAGTGGTCTTCAAATTGAAACAGTCATTGGGCAATTTCATGTTTTTG

>Eeu_CEACAM16N2

CGGCCACAGTGGCCATGACGATCGTGCCGGTGCCCGGGAAGCCCACGGAGGGCCAGGACGTGACGCTGACGGTGCAGGGCTACCCCAAGGACCTGCTGGTGTACGCCTGGTACCGCGGGCCCGCCGCCGAGCCCAACCGGCTGCTCAGCCAGCTGCCGTCGGGGAACTGGATCGCGGGCCCGGCGCACAGCGGCCGCGAGGTGGGCTTCGCCAACTGCTCGCTGCAGGTGCAGAAGCTCAACCTGACCGACGCCGGGCGCTACACGCTCAAGACCGTCACCCTGCAGGGCAAGACTGAGGCCCTGGAGGTGGAGCTGCAGGTGGCCC

*Echinops telfairi* (tenrec)

>Ete_CEACAM19N CCTCAATACTCTGGACGCTCCAGAGCTCCCAGGCGGACCTCTACATCCAGAAGGTTCCGGAAAAGCCTCTACAGAACGAGCACCTCCTCCTGTCCGTCCAGGGCATACCAGATGCCTTCCAGGATTTCAACTGGTACTATGGAGAGCGGACTGACGGTAGCACCATGCTGTTCTCCTACTTCCCAGGGCTGCAGCAGCCCCAGAGGAACGGCGACGCCAAGGGACAGCGTGACATAGTTGGCTTTGCCAATGGCTCCATGCTGCTGCGTCGCGCCCAGCCCAGCGACAGTGGCACCTACCAGGTGTTGGTCACCATCAAACCCTCCTGGACCATGCATGCCAACACCAGTGTCCAGGTGGTGG

*Felis catus* (cat)

>Fca_CEACAM1NtcTCACTCCTAACCTTCTGGAACCCTCCCACCACTGCCCAAGTCACTGTTGAATCGGTGCCGCCCAATGCTGCCGAAGGGAAGGACGTTCTTCTGCGTGTCCACAATCCGCCTGCCAATCTTATAGGCTTTGGCTGGTTCAAAGGGACAACTATAGATCCCCGTAGGGAAATTGTATCATATGCAGCAGACTCACAAGAAATTACCCCAGGATTTGCACACAGCGGCAGAGAGACATTATATCACAACGGATCCCTGCTGTTCCAGAACATCAGCCTGGAGGACACAGGATACTACACCCTACAAATCATAACAAGGAATCTTCAAGTTGAAAGAGTAACTGGACAGCTCCGTGTATATC

>Fca_CEACAM1-like_2N

TCTCACTCCTAACCTTCTGGAACCCTCCCACCACTGCCCAAGTCACTGTTGAATCGGTGCCGCCCAATGCTGCCGAAGGGAAGGACGTTCTTCTGCGTGTCCACAATCTGCCTGCCAATCTTATAGGCTTTGGCTGGTTCAAAGGGACAACTATAGATCCCCGTAGGGAAATTGTATCATATGCAGCAGACTCACAAGAAATTACCCTAGGATTTGCACACAGCGGCAGAGAGACATTATATCACAACGGATCCCTGCTGTTCCAGAACATCAGCCTGGAGGACACAGGATACTACACCCTACAAATCATAAAGAGGAATGTTCAAGTTGAAAGAGTAACTGGACAGCTCCGTGTATATC

>Fca_CEACAM1-like_1N

TCTCACTCCTAACCTTCTGGAACCCTCCCACCACTGCCCAAGTCACTGTTGAATCGGTGCCGCCCAATGCTGCCGAAGGGAAGGACGTTCTTCTGCGTGTCCACAATCTGCCTGCCAATCTTATAGGCTTTGGCTGGTTCAAAGGGGAAACTGTTGAGCCCCGTAGTGAAATTGTATCATATGCAGCAGACTCACAAGAAATTACCCCAGGATTTGCACACAGCGGCAGAGAGACATTATATCTCAACGGATCCCTGCTGTTCCAGAACATCAACCTGGAGGACACGGGATACTACACTGTACGAATCTTAAAGAGAAATTTTCAAATTGAAATAGTAACTAGACGGCTCCATGTATATC

>Fca_CEACAM16N1

CCGCCTTCCTGAGCGCGGGGGCCGAAATCTCTATCACCCCCGAGCCTGCCCAGCCAGCCGAGGGGGACAACGTCACACTGACCGTCCACGGGCTCTCGGGGGAAGTGCTTGCCTACAACTGGTACGCGGGGCCCACGCTCAGCCTGACGTACCTGGTGGCCAGCTACATCGTGAGCACGGGCGACGAGACCCCTGGGCCGGCCCACACGGGACGGGAGGCTGTGCGCCCCGACGGCGGCCTGGACATCCGGGGCGCCCTGCCCGGGCACTCGGGCACCTACATCCTGCAGACCCTCAACAGGCAGCTCCAGACGGACGTGGGCTACGGACACGTGCAGGTCTATG

>Fca_CEACAM16N2

CGGCAGCGGTCGCCATGACGATTGTGCCCGTGCCCACCAGGCCGATGGAGGGCCAGGATGTGACGCTGACCGTCCAGGGCTACCCCAAGGACCTGCTGGTCTACGCCTGGTACCGCGGGCCTGCCTCGGAGCCCGGCCGGCTGCTCAGCCAACTGCCGTCAGGCAACTGGATCGCAGGCCCCGCGCACACAGGCCGGGAGGTGGGCTTCGCCAACTGCTCACTGCTGGTGCAGAAGCTGAACCTCACGGATGCCGGCCGCTACACCCTCAAGACCGTCACGTTGCAGGGCAAGACCGAGACCCTGGAAGTGGAGCTGCAGGTGGCCC

>Fca_CEACAM18N partial

CCAGTCTACTGGCCTGTGGGACCCGCCAAGCCTACAGTCAAATCTCCATAGACCCAGAATCACTCTTAGGAATCAAGGGATTTCGGACCGTCCTGGTCCTCCAGAATGTCACCCAGGATGTTCAAGAATACAGCTGGCACCGAGGCGCAAATGATACTGCGGAAAATATGATTGTCAGCTACAAACCTCCCTCCGATACCTGGCAGTCTGGGCCCATGTTCAGTGGACGGGAGAATGTGACCAGGACAGGTAGCCTGGTGATCAGGAGGTCTGCATTAAATGACACAGG

>Fca_CEACAM19N

CCTCAATCCTGGCCCTCTGGGTCCCCCAAGGCTCCCGGGCTGCCCTCCGCATCCAGAAGATTCCAGAGCATCCTCAAAAGAACCAGGACCTGCTCCTGTCTGTCCGGGGCATCCCAGACACCTTTCAGGACTTCAACTGGTACCTGGGGGAGGAGGCCCACGGTGGCACGATGCTCTTCACCTACATCCCCGAGCTTCAGCGGCCCCAGAGGGACGGCAGTGCCATGGGGCAGCGTGACATCGTTGGCTTCCCCAATGGCTCCATGCTGCTGCATCGGGCCCAGCCCACCGACAGCGGCACCTACCAGGTCGCTGTCACCATCAATCCTGCCTGGACCATGAGGGCCAAGACCAAGGTCCAGGTGGAGG

*Loxodonta africana* (elephant)

>Laf_CEACAM1N

TCTCACTTTTAATCTTCTGGAGCCCATCCATCCCAGCCCAACCCACCATTGAATCGGTGCCACTCAATGCTGCTGAAGGAAATGATGTTCTTCTGCTTCTCAGCAATCCACCAGAAGACATTTTCGTCTACATCTGGTACAAAGGGGAAAGTACAGGAAACAATGATAGAATTGTATCATATGCATCACAAGTTCAAATACAAGCTTTGGGGCCTGCCTACACTGGTCGAGAGACAATATACCACAACGGGTCGCTGCTGATCCAGAATGTCACCCTGAACCACTCAGGAGTCTACACACTACAAATAACAACCGTGTTTGGTCCCACTGAGATAGCTGGACAGATCCGCGTATACC

>Laf_CEACAM16N1

CGGCCTTCCTGAGTGCCAGGGCAGAGATTTCCATCACCCCTGAGCCTGCCCAGCCGGTCGAGGGGGACAACCTCACGCTGGTCGTGCATGGACTCTCAGGGGAGCTGCTCGCCTACAACTGGTACGCGGGGCCTATGCTCAATGTGGCCTACCTGGTGGCCAGCTACATCGTGAGCACAGGCGATGAGACCCCTGGCCCAGTCCACACGGGGCGAGAGGCTGTGCGCCCCGACGGTAGCCTGAACATCCAGGGCGCCTTGCCTGGGCATTCAGGCACCTACATCCTGCAGACCCTCAACAGGCAGTTTCAGACCGAGGTGGCCTACGGACACGTACAGGTCTATG

>Laf_CEACAM16N2

CGGTGGCAGTTGCCACAATGATCGTGCCCGTGCCAACCAAGCCGACGGAGGGCCAGGACGTGACCTTGACTGTGCAGGGCTACCCCAAGGACCTGCTGGTCTATGCCTGGTACCGAGGGCCTGTCTCTGAGCCCAACCGGGTGCTCAGCCAACTGCCGTCGGGGAACTGGATTGCAGGCCCCGCACACACGGGCCGCGAGGTGGGCTTTGCCAACTGCTCGCTGCTGGTGCAGAAGCTGAACCTCACGGACGCCGGCCGCTACACACTCAAGACCATCACCCTTCAGGGCAAGACCGAGACGCTGGAGGTGGAGCTGCAGGTGGCCC

>Laf_CEACAM19N

CCTCAATCCTGGCCCTCTGGACCCTCCAAAGCTCCCAGGCGGCCCTCCACATCCAGAAGATTCCAGAGCAGCCTCTAAAGGACCAGGACCTCCTCCTGTCAGTCCAGGGCATCCCAGGCACCTTCCAGGACTTCAACTGGTACCAGGGGGAGGAGACCTCCGGCAGCACAATGCTGTTCTCCTACATTCCAGGGCTACAGCGGCCCCAGAGGGATGGCAGTGCCATGCGGCAGCGCGACATCGTTGGTTTCGCCAACGGCTCCATGCTGCTGCGCCGTGCCCAGCCCAGCGACAGTGGCATCTACCAGGTGGCCGTCACCATCAACCCCTCCTGGACCATGAAGGCCAAGACCGAGGTCCGGGTGTCTG

>Laf_CEACAM300N

TCTCACTTTTAATCTTCTGGAGCCCATCCATCCCAGCCCAACCCACCATTGAATCGGTGCCACTCAATGCTGCCGAAGGAAATGATGTTCTTCTACTTCTTCAAAACCCACCAGAAAACATTTTCATCTACATCTGGTACAAAGGGAAAAGTGCAGGAAACAACAATAGAATTGTATCATATACACCACAAGCTCAAGTAAATGATTCAGGGCCTGCCTACACTGGTCGAGAGACAATATACCACAACGGGTCGCTGCTGTTCCAGAATGTCACCCTGAACCACACAGGAGTCTACACACTGCAAATATCAACCATACTTGGTGTCACTCAGATAGCTGGGCAGATCCGCGTATACC

>Laf_CEACAM301N

TCTCACTTTTAATCTTCTGGAGCCCATCCATCCCAGCCCAACCCACCATTGAATCGGTGCCACTCAATGCTGCTGAAGGAAATGATGTTCTTCTGCTTCTCAGCAATCCACCAGAAGACATTTTCGTCTACATCTGGTACAAAGGGGAAAGTACAGGAAACAATGATAGAATTGTATCATATGCATCACAAGTTCAAATACAAGCTTTGGGGCCTGCCTACACTGGTCGAGAGACAATATACCACAACGGGTCGCTGCTGATCCAGAATGTCACCCTGAACCACTCAGGAGTCTACACACTACAAATAACAACCGTGTTTGGTCCCACTGAGATAGCTGGACAGATCCGCGTATACC

>Laf_CEACAM302N pseudogene

tctcacttttaatcttctggagcccatccatcccagcccaacccaccattgaatcggtgccactcaatgctgccgaaggaaatgatgttcttacttccccataatccgtcagcagATCCTTTATTCTGCGTCTGGTACGAAGGGGAAAGTATAGGAAACGATAAAATTGCATCATATATACCACAAGCTCAATCAAATGATTCAGGGCCTGCCTACACTGGTCGAGAGACAATATACCACAACGGGTCGCTGCTGTTCCAGAATGTCACCCTGAACCACTCAGGAGTCTACACACTGCAAATAACAACCATACTTGGTGACAGTGAGATATTAGCTGGACAGATCTGTGCATACT

>Laf_CEACAM303N pseudogene

TCTCACTTTTAATCCTCTGGAGCACATCCATCACAGCCCAACTCACTAACAAATCCATGCCGTTCAACACTGTTGAACGAAATTATGTTATTCTACATCTTCACAACCTACCAGCAGGTCCTTTAATCTATATCTGGCACAAAGGAAAACATTTAGACAACAACAATAAAATTGTATCATTATCTATCACTAAGTCAATCAAATATCCCAGGGCCTATCCACAGCAGTCGGAAGACAATATACCCTAATGGGTCCCTGCTGTTCCAGAATGTCACCCTGGAGAACACAGAAAACTACATACTACAAATAACAAAGACAAATGGTGACAAAGAAATATTAGCTGGACAGATCCGTGTCTACC

>Laf_CEACAM304N pseudogene

TCTCACTTTTAATTCTCTGGGGCCCATCCATCACAGCCCAAATCACTACTGAATCCATGTCATTCAATGCTGCCGAAGGAAATGACGTTCTCCTACTTCTCCACTATCTGCCAACAGATTCTTTAATCTACATCTGGTCTAAGGGTAAAGTACAGACAACAATAAAATTGTTCAATATCTACCACAAAATCAATCAAATACTCCACTGCCTAACCACAGAGGTCGAGAGACAGTATACCACAATAGGTCCCTGCTCTTCCAAAATGTCACCCTGAAGGACACAGGAATCTACACACTACAAATAACAAAGACAAATGGTGACAAAGAAATATTAGCTGAACAGATCCGTGTATACC

*Monodelphis domestica* (grey short-tailed opossum)

>Mdo_CEACAM1N

CCTCCATCCTCAGCTGCTGGAGCCAGCCTACGTCTGCTCAGGATCTCACTGTTGTACCAGAACCGGCCTATGGGAAAGAAGGCGACACAGTCACCCTGGTGGTCCGGGGGTTCTCAGGAACACCTTTGAGCTATACTTGGTACACTAAGTTATCAGCAGAGGATCCCAAAGACACTGAGATGGTCAGGTACATTGTTAGTAACAGTATGCAGACACCAAACGATATCCGGCAGAGGGTGTTCCCCAATGGCTCCCTGCTCATCCCCAACCTCGTCCTCAATGACACTAAATACTACACTGTACAGATGGTCGATACCATTGGAAATATACTTAGAGGAGGAGCCCAACTGACTGTGTATG

>Mdo_CEACAM16N1

CTGTCTTTCTAATGGCCTGGATCCCACCAGCCCCAGCCCTGCTGAACATCTTAGTCATCCCGGAGCCACCAGCCCAGGGCGACAACGTCACCCTGTTGGTGCGAGGCCTCCCAGGGGAGCTTCTGGCTTACAACTGGTACCGAGGGACGAACCTCAACCAGGCCCACCTCATCCTGAGCTATATCATCAGTACAGCTGACGAGACCCCTGGGCCAGCCTACACGGGCCGCGAAGCTGTCCGCCCGGACGGGAGTCTGAACCTCCGGGATGTGGTCCCTGAGGACTCCGGCAGCTACATCCTGCAGACCCTGAACAAGCAGTTCCAGACCGACATTGCCTATGGCCACCTCCTAGTCCATG

>Mdo_CEACAM16N2

TCGCCACTTCGGCCATGACGATAGTGCCCGTTCCTTCCAAGCCCACCGAGGGGAAGGACGTCATCCTGTCTGTCCAAGGCTATCCCAAGGATCTTCTGGTCTACGCCTGGTACCGAGGGACCGCCTCCGAGCCCAACCGCCTCCTCAGCCAGCTGCCCTCGGGAAACTGGATCGCAGGGCCAGCCCACACCGGGAGGGAGATGGGTTTTGCCAATTGCTCCTTGCTCATCCAGAAGTTAAACCTCACAGACACTGGCCGCTACACTCTGAAGACCGTCACCCTGCAGGGCAAGACGGAGACACTGGATGTCCAATTGCAGGTCTCAA

>Mdo_CEACAM18aN

TGGTCCTGCTGGCCTCCACTGTAGCTGCTGAATTGACTCTCAACCCAGAACACCTTGATGAGGAGAAGGACGGTTCACTCATCTGGAGGATCCAAGGAGCCCCCACCGGTGATGTCAACTATACCTGGTACCGGGGGAATGGATCCCTCGAAGAAAACATGCTTATCTCCTACAACTCCTCCTCTCAAAGCAGGATCTGTGGCCCTGAAAACACAGGCCGGGAGAATGTGACTGACAAAGGCTTCCTGACGATCACAAAATTACAATTCAATGACTCTGGGATCTATACACTGGGGGTGACCAGTCCTGGGGACTATCAAGTGGCCACTGGACAGATACAGATCTGGG

>Mdo_CEACAM18bN

CTATCCTGCTGAGCTTCTGGACTCACCCAGGATTTGCTGAGATCTTCATCAACACAGTCCCAACCCAACCTATGGAAGGAGGCAACATGCTTTTGATTGCCTTGAACACTCCAGATGATATCCTCAATGCCACCTGGTATTCTTGGTTCCGGCCCTGGTATCCTGCCATGATCGTCAGTTGCATCCCCACTAACAGGACAGGCCCCTGTGCCCAAGTCTTCAGTCCTGCCCACACCACGAGAAAGATTCTGACACACTGGAACATCTTGGAAATCAGAAACCTCACTCTCTCTGACACTGGTTTCTACTCCTTGCTACTGGACACTGAGTCAGGACCAAGGAATGCATCTACCTACATCAAAGTGAAGCAAGCAGATG

>Mdo_CEACAM19N

CTTCCCTCTTGGCCTGGTGGACTGTCCAGCAGGCCTGGGCTGGTCTCCTGATTGAGAAGATTCCCGAGACACCCCAGGAGGGTCAAGATGTCCTTCTAACAGTTCATGGTGTCCCAGCGGCCATCAAGGATTTCAACTGGTACCAAGGAGAGGAGGTTGATGGCAGCACCATGATCTTCTCCTACTTCCCAGGCCTCCCGAGGCCCCAGAGGAATGGGAATGCCCTACAGGGAAGGAACATCATTGGGTTTCCTAATGGTTCCTTCCTCCTCCGCCATGTCCAGCTCACAGACAGTGGCATCTACCAAGTGGGCATCACCTTCAACCCATCCTGGATCATGAGGGCCAAGACTGAGCTCAAAGTCATAG

>Mdo_CEACAM20N

CCTCTCTCTTTGCCTCGTGGAGCTTTCAAGGGGCCGCGCAGCTCTCCATCGTCTCCCAGCCGTCCATTGCTGTTGTGAGTGAGACCGTCCTTCTCTCCCTCCATGGGCTCCAGGAGGGAAGGTATTCGACCCTGACCTGGTATCGAGGGCTGGATTTTATACACGCCATCATGTCCTACAATGCCGACACGGGGGTCCACAACGAGGGCTCCGCGTTTACGGGCCGGGAGAGCCTGCCAGCCAACGGCTCCCTCATCATCAATGAAGTATACTTGAACGACTCTGGGCACTACAGCATCCTCGTGAAGAGCAGGGAGGGCCCGCTGATGTCCGCGAACGGAAGTTTCCAAGTGTTTG

>Mdo_CEACAM22N

CCTTCCTTCTGTGCTTCCAACTCCAACTGACACCTGCTGAACTAACCATCACTCCAATCCCATCCAGACCAGTAGAAGGGGAAAACATCACCCTCTTCGTCCAGGGCTTTATTGGAGAAGATCTCATGTCCTTCAACTGGTTCCGAGGGAGTACCACAGATCTGGCCCAACAGATCCTCACCTTTGTATCCATGTCTGGCACCCAGTCAATGGGGAAGGCCTACACTGGGAGAGAGAGAGCAGAGGCTGATGGCTCCCTGCATATTTCCAGCTCCCACCTCAATGACTCAGGCATCTATACTATTCGCCAGATCTTCTTTCCAACAGACTCAGGGTCAAATATGGTGTCTACAAATGTCTACTTGCCTGTCTATG

>Mdo_CEACAM100N

CCTCCATCCTCAGCTGCTGGAGCCAGCCAACGTCTGCTCAAGGTTTCACTATTGACCCAAACCCGGCCAATGGGAAAGTAGGGGACACCTTCATCCTGAATATCCTGGGGTACTCGGGGCCAATAGTAAAAACGTATTCTTGGCAATATAAGAAATCAGAACATGATCCCAACGTGATTGCAATGTATGAATTGGAGGTTGCTACTAACCAACACAACCCACCGAATAGCCGAATTAGCGTGTTCTCTAATGGCTCCCTGAAAATCCCCAACCTCCAACTCAGTGACTCTGGTTACTACCTTGCATACATCACCCAGCAAACTTCCAATCAATTGAGAGCAGGAGTCCCACTGACGGTGCATG

>Mdo_CEACAM101N

CCTCCATCCTCAGCTGCTGGAGCCAGCCAACGTCTGCTCAAGGTTTCTCTGTTGTCCAAACCACCCACCCCCATGTGAGAATAGGCAGTACAGTCATCCTGATTGTCCAGGGGTTCACAGGGACAACTTTTGCCTATAATTGGTACACTAAGTCATCAGAACAGGATCACAACAAGGATAATATCTTCCAATACAATCCTGGTTCTGGAAAGCAGGATCCAGAAAATACCCCATTCCCAATGCTCTCTAATGGCTCTCTGCACATCCCCAAGCTCATCCTTGATGACTGGTCCTTACTACTACCTTTTAGAAATTCTCAATAACTCTTTCAGGATACTTAGAGCAGGAGTCCCACTGATGGTTCATG

>Mdo_CEACAM102N

CCTCCATCCTCAGCTGCTGGAGCCAGCCTACGTCTGCTTGGGAACTCACTATTGTACCAGAACCAACCTATGTGGCAGCAGGTGAAACGCAAGTCCTGAATTTCAAGGGGTTCAATGGGACAGCTGTGAAGTACGTTTGGAACTATAAATATGTAGAATCTGATACCAACTATAATTTATTATACGAATACAACGTTCTTACTGGACAGCAGCACCCGAGTCAAGGACGGGTGCATGTGAGCCCTAATGGCTCCCTCGTCATCCCTCGTGCCAACGAGTATGACGTTCGTTACTACATTTTAGAAGTGTTTGACAACGCTTCCCACGTATATAGAGGACAATTCGGCCTTTTTCTGTATG

>Mdo_CEACAM103N

CCTCCATCCTCAGCTGCTGGAGCCAGCCAACGTCTGCTCAAGATATCACAGTTGAAGTAGAGCCACCCCTTGGGACACAAGGCTTAAGCGTCACCTTTCGTATCGTGGGGTACTCGGGGACTCCTGTTATGTATAATTGGTACTATAAGACATCAGCTCAGCAACCCAGCAGGAAACATATCGTGACATACAGTACTTATAATAGAAGACAGTTACAGACCAATATTCGGCAGAGGGTATACCATAATGGCTCCATGTTTTTCCCCAACCTCCTCGTCAATGACTCTGGTTACTATGAAGTACAGATCATCAAAGATAGGTGGACTCTGGACAGACCTGAAGCAGGAGCCCCACTGAAGGTGTATG

>Mdo_CEACAM104N

CCACCATCCTCAGCTGCTGGATCCAGCCAACGTCTGCTCAAGATATCACAGTTGAAGTAGAGCCACCCCTTGGGACACAAGGCTTAAGCGTCACCTTTCGTATCGTGGGGTACTCGGGGACTCCTGTTATGTATAATTGGTACTATAAGACATCAGCTCAGCAACCCAGCAGGAAACATATCGTGACATACAGTACTTATAATAGAAGACAGTTACAGACCAAAATTCGGCAGAGGGTATACCATAATGGCACCATGTTTTTCCCCAACCTCCTCGTCAGTGACTCTGGTTACTATGAAGTACAGATCATCAAAGATAGGTTCACTCTGGACAGACCTGAAGCAGGAGCCCCACTGAAGGTGTATG

>Mdo_CEACAM105N

ATAAGTCCTCCATCCTCAGCTGCTGGATCCAGCCAATGTCTGCTCGAGGTCTCACTGTTGTGCCAAACCCGGCCAATGGGAAAGTAGGTGCCGGCTTCAGCCTGAGGATCCAGGGGTACTCAGGGCCGGCTATTAAGTATAATTGGTACTATAGGTTATCAGCACGGGATCCCAATGAGAAACTGATCGGGTGGTTCAATCCTATTTCTAAAACATACTCAGGAGGCCAGAAGTATGTGGCGTTCCTTGATACCTCCATACGCATCTCCAATCTTGGCTTAAATGATTCTGGTTACTACGAAGTAGAGGTCATCAAAGACATGAAATCTCAGGACAAACCTAGAGCTGGAACCCAACTGACAGTGTATG

>Mdo_CEACAM106N

CCACCATCCTCAGCTGCTGGATCCAGCCAATGTCTGCTCAGGATCTCATGGTTGAACTAAAACCAGCCCAGGGGAAAGTAGGGGACACCGTCACCCTGAATATCCTGGATTACTCTGGGAGTGCTCTTATGTACAGTTGGTACTATAAGCCATCAACACGGAATTCCAAGGAGAAACTCATCTTGAGGTACTCTCCTGGTTCTACATCTCAGACACCAAACAATACCCGACAGAAGGTACACCCTAATGGCTCCTTGATCATCCCCAACCTTGTCCTCAGTGATTCTGGTGACTATAAAGTACAGATCAAAGATTCCTCTTCAAAGATACTTAGAGCAAGAGCCCCACTGAAGGTGTATG

>Mdo_CEACAM107N

CCACCATCCTCAGCTGCTGGATCCAGCCAACGTCTGCTCAAGGTTTCACTGTTGTACAAACCCCAGCCACTGTGAAAGAAGGCGACAACGTCATCCTGAATATCCAGGGGTACACGGGGAGTGCTCGTGAGTATGTTTGGTACCGAAAGAAATCAGCTCAGGATCCCGGCCGTGTAAAGATCGTGACATTCTATAGTAATCATTTATATCAGTCACCAAGCAATATCCGGCAGAAGGTACTCGCTAATGGCTCCTTGATCATCCCCAACCTCATTGTCATTGATGATGGTTACTATGAAGTACAAATCAACGATTCCTCTTCACAGATACTTAAAGGAGGAGCCCCACTGAAGGTGTATG

>Mdo_CEACAM108N

CCTCCATCCTCAGCTGCTGGATCCAGCCAACGTCTGCTCAAGATATCACAGTTGTACCACAACCAACCTATGTGCAAGAAAGTGACAACGTCATCCTGAATATCCAGGGGTACACGGGGAGTGCTCGTGAGTATGTTTGGTACCGAAAGAAATCAGCTCAGGATCCCGGCCGTGTGAAGATCGTGACATACAATCCTGCTACTAAATATCTGTCACCAAGCAATTCCCGACAGAGGGTATTCGCTAATGGCTCCTTGATCATCCCCAACCTCGTTGGCATTGATGATGCTTACTATGAAGTACAAATCAACGATTCCTCTTCACAGATACTTACAGGAGGAGCCCTAGTGACAGTCTATG

>Mdo_CEACAM109N

CCTCCATCCTCAGCTGCTGGAGCCAGCCAACGTCTGCTCAGGATCTCACTGTTGTACCAGACCCAGTTTATGGGAGAGTAGGGGACACCGTCACCCTTAATGTCCTGGAGTACTCAGGGAGGGCTTTGGGCTATAATTGGTACTATAAGTCATCAGCACAGGATCCTGACCTGACTTTGCTCATCCAACACAATGTTCAAGATGGAAAGCTGATACCAGAGGATACCCGACAGAAGGTACTCCCTAATGGCTCCCTGCTCATCCCCAATCTTGTCCTCAATGATGCTGGTTACTACGAAGTATGGATCAATGATTCCTCTTTACAGATACTTACAGGAGGAGCCCAACTGACAGTGTATG

>Mdo_CEACAM110N

CCTCCATCCTCAGCTGCTGGAGCCAGCCAACGTCTGCTCAGGATCTCACTGTTGTACCAATCCCGGCCAATGTGAAAGAAGGCGACAGAGTCATCCTGGTGGTCCAGGGGGTCTCAGGGAAGCCTTTGAGCTACACTTGGTTCAGTAGATCACCAGGAGATTCAAGCGACACTGAGATCGTCAGGTACATTGTTGGTAACAGAACACGGATACCAGTCAATATCCGGCAGAAGGTGCTCCCCAATGGCTCCCTGCTCATCCCCAACCTCCTCATCAGTGACACTAAATACTACACTGTACAGATGGTCGATACCTTTGGAAATATACTTAGAGGAGGAGCCCCACTGACAGTGTATG

>Mdo_CEACAM111N

CCTCCATCCTTAGCTGTTGGATCCAGTCAACATCTGCTCAAGATGATCCTGTTCGTATTGTGCCCATCCCACCCTATGGGACTGTGGGCAGCAACGTCCTCTTGACCATCTTGGGATTCACAGAGAAGGGTAGCACCTATACTTGGTACAGAAAGACAAGTGAGGTTTCCAATGAGATTGCAAGCTATGAATTTGATACTAGAGTACAGACACCAGAAGATAGCCGAGAGAAAATACTCCCTGATGGTGCCCTGCTCATCCCCAACCTCAGCCTCAGTGATGCCAGTATCTACATTCTGCATATTACTATTTACAAAGTTGGAGTTGCAGTATATGAAGTAATAACCCAATTAAGAGTGTATG

>Mdo_CEACAM112N

TCTCCATCCTAAGCTTCTGGATCAAGCCAATATCTGCCAAAGGTGCTTCTGTCAGTGTTGTGCCAAGTCCACCCTATGGGAGAATGGGAAGCAACGTCACCTTGACAATCCATGGATTCTCAGAGCAGGCTCTCAGCTATAATTGGTACAGAAAGACAACAGAGGACTCCAACAAGATCATTAGCTACAGTGTCCCTTCTAGAATGCAGATTCCAGCAGATATCCGGGAGAAGGTATACCCCAATGGCTTTTTGCTCATTCCCAACCTCACCCTCAGTGACACTGAGCTCTACATTGTACAAATTGTTGATTCCAGAGGTGTGATAGCTGTAATAGCACAAGGACAATTGAGAGTGTATG

>Mdo_CEACAM113N

CCTCCATCCTCCATTGCTGGATGCCACCTAGGTCTGCCCAAGGTACTCCAGTTAGTATCGTGCCACGCCCCCCATATGGGACCGTGGGCAGCAACGTCACCTTGACCATTGAGGGCTTCTCGGAGCGGGCCCTCAGCTATAATTGGTACAGAAAGACAACGGAGAACTCTAACAGGATCGTCAGCTACAGCACTCTTACAGGGGTGCAGAAACCTACCGATATCCGGGAGAGGGTGCAGCCCAGGGGCTTCCTATTTATTCCTCACCTCACCCTCGGGGACGATGACCTCTACGTTGTACAAATTGTTGATTCTAGGGGAGTGATAGCTGCAATAGTAAGAGGAAAATTACCAGTATACG

>Mdo_CEACAM114N

CCTCCTTCTTCAGCTGCTGGATCCAGCCCCAGTCTGCTCAAGGCACTCTGGTCAGTGTTGTGCCAAGACCACCCTATGGGACAGTGGGCAGCAATGTCACCTTGATGATCCAAGGGTACTCAGGGAGCATTTTCAGCTATAATTGGTTCAAAAATACAACAGAGGTCTCCAACAGGATCATAACCTACACGGTTCCTGTTAAAGAGCAGATGCCAGCAGATAACAGGGAGAGGGGGTTCCCCAATGGCTCCCTGCTCATCCCTAACCTCACCCTCAATGACACTGAAGTCTACATTGTACAAATTGTTAATTCTGAAGGTATGATAGCCACAGATGCAAAAGTGCAATTCAGAGTGTATG

>Mdo_CEACAM115N

CCTCCATCCTTAGCTGTTGGATCCAGTCAACATCTGCTCAAGGTAATCCTGTTCGTATTGTGCCCATCCCACCCTATGGGACTGTGGGCAGCAACGTCCTCTTGACCATCTTGGGATTCACAGAGAAGGGTAGCACCTATACTTGGTACAGAAAGACAAGAGAGGCTTCCAACCAGATTGCAAGCTACTCTTTACGTACTAGAGTACAGACGCCAGAAGATAGCCGAGAGAAAATACTCCCTGATGGTGCCCTGCTCATCCCCAACCTCAGCCTCAGTGATGCCAGTATCTACATTCTGAAAATTACTATTTACAGTGTTGGAGTTGGAGTATATGAAGTAATAACCCAATTAAGAGTGTATG

>Mdo_CEACAM116N

CCTCCATCCTCAGCTGCTGGAGCCAGCCAACGTCTGCTCAGGATCTCACTGTTGTACCAGAACCGGCCAACGTGAAAGAAGGCGACAACGTCACCCTGGTGCTCCAGGGGGTCTCAGGAACAACTTTGAGCTATGCTTGGTTTAGTAGATCACCAGGAGATTCCAGTGACACTGAGATGGTCATGTACTCTGTTATTTACAGTATGCAGACACCAGCCAATATCCGGCAGAAGGTGCTCCCCAATGGCTCCCTGCTCATCCCCAACCTCGTCCTCAATGACACTAAATACTACACTGTACAGATGGTCGATACCTTTGGAAAGATACTTAGAGGAGGAGCCCCACTGACAGTGTATG

>Mdo_CEACAM117N

CCTCCATCCTCAGCTGCTGGAGCCAGCCAACGTCTGCTCAAGGTCTCACTGTTGTACCAGAACCGGCCAATGTGAAAGAAGGCGACAACGTCACCCTGGTGGTCCAGGGGTTCTCAGGAACAACTTTGAGCTATGCTTGGTTTAGTAGATCACCAGGAGATTCCAGTGACACTCAGATGGTCATGTACTCTGTTATTTACAGTATGCAGACACCAGCCAATATCCGGCAGAAGGTGCTCCCCAATGGCTCCCTGCTCATCCCCAACCTCGTCCTCAATGACACTAAATACTACACTGTACAGATGGTCGATACCTTTGGAAAGATACTTAGAGGAGGAGCCCCACTGACAGTGTATG

>Mdo_CEACAM118N

CCTTCATCCTCAGCTGCTGGATCCAGCCAAAGTCTGCTCAAGGTCTCACTGTTGTAGAGTACCCTCTCCATGTGAAAGAAGGCGACACCGTCCTCCTGAATATCCAGGGGTACTCAGGGACACCTTTGAGGTATATTTGGTACACTAGATCATCAGCAGATTCCAGAGACACTGAGATCGTCAGGTACTCTGTTAGAAGAAGGCAGAGACCAGACAATATCCGGGAGACAGTGCTCCACAATGGCTCCCTGCTCATCCCCAACCTTGTCCTTGATGACACTAAATACTACACTGTAGAGATCCTTGATTCTTATTTACAGATATTTAGAGGAGGAGTCCAACTGATGGTGTATG

>Mdo_CEACAM119N

CCTCCGTGCTCAGCTGCTGGAGCCAGCCGACATCTGTTCTAGCGTCTTCCATCACGGCTGTGCCAAGCCCGCCCTATGGGATGGTGGGCAGCAATGTCACCTTGACCATCCAGGGCTTCCTGAAGCCCCCTTGGAGGTACACCTGGTACAGGAAGTCAACAGACCCCTCCAACGAGATCGCCAGCTACGTCGTTCGTGATGGAGTACAGACCCCGGCCACGAACCGGGAGAAGGTGTTCGCCAACGGCTCTCTGCTCATCCCCAACCTCACCTTCAGGGACAACGACGACTACATCGTCCAAGTCTTCTACTCAGAATCGGAAGTAACAACGGCCAGGGCACACCTCCAGGTGTTCA

>Mdo_CEACAM120N

CCTCCATCCTCAGCTGCTGGATCCAGCCAACGTCTGCTCAAGATATCACAGTTGAAGTAGAGCCACCCCTTGGGACACAAGGCTTAAGCGTCACCTTTCATATCGTGGGGTACTCGGGGACTCCTGTTATGTATAATTGGTACTATAAGACATCAGCTCAGCAACCCAGCAGGAAACATATCGTGACATACAGTACTTATAATAGAAGACAGTTACAGACCAATATTCGGCAGAGGGTATACCATAATGGCTCCATGTTTTTCCCCAACCTCCTCGTCAATGACTCTGGTTACTATGAAGTACAGATCATCAAAGATAGGTGGACTCTGGACAGACCTGAAGCAGGAGCCCCACTGAAGGTGTATG

>Mdo_CEACAM121N

CCACCATCCTCAGCTGCTGGATCCAGCCAACGTCTGCTCAAGATATCACAGTTGAAGTAGAGCCACCCCTTGGGACACAAGGCTTAAGCGTCACCTTTCGTATCGTGGGGTACTCGGGGACTCCTGTTATGTATAATTGGTACTATAAGACATCAGCTCAGCAACCCAGCAGGAAACATATCGTGACATACAGTACTTATAATAGAAGACAGTTACAGACCAAAATTCGGCAGAGGGTATACCATAATGGCACCATGTTTTTCCCCAACCTCCTCGTCAATGACTCTGGTTACTATGAAGTACAGATCATCAAAGATAGGTGGACTCTGGACAGACCTGAAGCAGGAGCCCCACTGACTGTGTATG

>Mdo_CEACAM122N

CCACCATCCTCAGCTGCTGGATCCAGCCAACGTCTGCTCAAGATATCACAGTTGAAGTAGAGCCACCCCTTGGGACACAAGGCTTAAGCGTCACCTTTCGTATCCTGGGGTACTCGGGGACTCCTGTTATGTATAATTGGTACTATAAGACATCAGCTCAGCAACCCAGCAGGAAACATATCGTGACATACAGTACTTATAATAGAAGACAGTTAGAGACCAATATTCGGCAGAGGGTATACCATAATGGCACCATGTTTTTCCCCAACCTCCTCGTCAATGACTCTGGTTACTATGAAGTACAGATCATCAAAGATAGGTGGACTCTGGACAGACCCGAAGCAGGAGCCCCACTGAAGGTGTATG

*Myotis lucifugus* (small brown bat)

>Mlu_CEACAM16N1

CCGCGTTCCTGAGCACCGGGGCGGAGATCTCCATCACCCCGGAGCCCGACCAGCCGGCCGAAGGGGACAACGTCACGCTGGCTGTCCGCGGGCTGTCGGGGGAGCTGCTCGCCTACAGCTGGTATGCGGGGCCCACGCTCAGCCTGGCTTACCTGGTGTCCAGCTACATCGTAAGCACAGGCGACGAGACCCCCGGCCCGGCCCACACGGGGCGGGAAGCTGTGCGCCCGGACGGCAGCCTGGACATTCAGGGCGCCCTGCCTGGGCACTCGGGCACCTACATCCTTCAGACTCTCAACAGGCAGCTCCAGACGGAGGTGGGCTACGGACACTTGCGAGTCTATG

>Mlu_CEACAM16N2

CGGCTGTCGTGGCCATGTCGATCGTGCCCGTGCCGACCAAGCCGACGGAGGGCCAGGACGTGACGCTGACCGTCCAGGGCTACCCCAAGGACCTGCTGGTCTACGCCTGGTACCGCGGGCCTGCCTCCGAGCCCAACCGGCTGCTCAGCCAGCTGCCGTCCGGGAACTGGATCGCAGGCCCCGCGCACACCGGCCGGGAGGTGGGCTTCGCCAACTGCTCGCTGCTGGTGCAGAAAGTGAACCTCACGGACGCCGGGCGCTACACGCTCAAGACCGTCACGCTGCAGGGCAAGACGGAGACGCTGGAGCTGGAGCTGCAGGTGGCCC

>Mlu_CEACAM18N

CAAGTCTGCTGGCATGGGGGATCTGCCAGGCCTCTGGCCAAATCTCCATCATCTCATCCCTAGGGATTGAGGGATATGAGGCCCTCCTGGAACTGCAGAATGTCCCCGAGGGTGTGCAGGAATACAGCTGGCACCGGGGTGCAAGTGACAGTGCAGACTCGATGATTGTCAGCTACCAACCTCCCTCCCAGGCCTGGCAGCCTGGGCCCATGTACAGCAGCCGGGAGAACGTGACCGTGAAAGGCCACCTGGTGATCAAGGACTCCCTGTTAAACGACACGGGGAGCTACACGGTGAGGGTGGACCTCGGCAACGAGACCCAAAGAGCAACCGGCTGGCTGGAGATTCACG

>Mlu_CEACAM19N

CCTCAGTCCTGGCCCTCTGGGTCCCCCAAGGCTCCTGGGCGGCCCTCCGCATCCAGAAGATTCCAGAGCGACCTCAAAAGAACCAGGACGTGCTCCTGTCTGTCCAGGGCATCCCAGACACCTTCCAAGACTTCATCTGGTACCGGGGGAAGGAGACAGATGGTGGCACAAGGCTGTTCACCTACATCCCTGATATACCGCGGCCCCAGAGAGACGGCAGCGCCATGGAGCAGCGCGACATCGTTGGCTTCCCCAACGGCTCCATGCTGCTGCGGGACGCCCAGCCCAGAGACAGCGGCACCTACCACGTGGAGGTCCACATCAACCCTTCGTGGACCATGCGGGCCAAGACTGAGGTCCAGGTGCCCG

>Mlu_CEACAM1-like_1N

TTTCACTCTTAAATTTCTGGAGCCTGCCCACCACTGCCCAACTCTCTATTGTGTCAACCAATGCTGCCGAAGGACAGGATGTTACTCTACGTATCTGCAATATGCCTCACAATGCTCAAGGCTACATGTGGTACAGGGGGGAAGGGGCAAACTTCACACATAAAATTGCAGGTCTTGGATTGTTCCCAGGACATAGAACAGGGCCTGCACACAGCGGTCGAGAGTATATAAACTTTGATGGATCCTTGGTGATAAAGAGGGTCACCCTGGAGGACACAGGAATCTACACCGTAGTAGTCTTCCTTCCAGAGCATAAAAAAGAAATAGGATTTGGACCACTCAATGTATACG

>Mlu_CEACAM1-like_2N

CCACACTCTTCACCTTCTGGAGCCTGCCCACCACTGCCCAGCTCACTATTGAATCAGTGCCACCCAATGCTGCCGAAGGGAAGGATGTGCTTCTCCGTGTCCACAACCTGCCTGGGAATCTTGGAGGCTATGCCTGGTACAAAGGAGAAGTAGTGGACAGCAACCATAAAATTGTATCATATGTAATAGACACTCAAAAAATTACCTACGGACCTGCATACAGTGATCGAGAGAAAATATATCCCAATGGATCCCTGCTGTTTCAGAACGTCACCCGGAAGGACACAGGATACTATGTCCTACTAGCCATAGACAAAAATTTTCAGAGCAGACCAGTAACTGGACAGCTCCATGTATACC

>Mlu_CEACAM1-like_3N

TTTCACTCTTAAATTTCTGGAGCCTGCCCACCACTGCCCAACTTGCTATTGTGTCAACCAATGCTGCCGAAGGGAAGGATGTGACTCTACGTATCCGCAATATGCCTCCTGATTATATAGGCTTCGTGTGGTACAGGGGGGAAGGAGCAAACTACAGACATACCATTGCCAGTCTTTCATTCCGCGTAAGAACTTCTAGAACAGGGCGTGCATACAGCGGTCGAGAGCAAATAAATTTTGATGGATCCCTGCTGATAAAGAGGGTAACCCTGAAGGACACAGGAATCTACACCATAGTAGTCTACCTTCGAGACTATAAGAAAGAAATAGGATTTGGAAGGCTCAATGTATATG

>Mlu_CEACAM1-like_4N

TTTCACTCTTAAATTTCTGGAGCCTGCCCACCACTGCCCAACTCGCTATTGTGTCAACCAATGCTGCCGAAGGGAAGGATGTGATTCTACGTATCCGCAATAAGCCTCCCAATGCTATAGGCTTCACGTGGTACCGGGGGGAAGGGGCAAACTACTATCATCATATTGCAACTCTCACAACACACATAAGAGTTTATAAAACAGGGCATGCATACAGCGGTCGAGAGCAAATAAACTATGATGGATCCTTGCAATTAAAGCAGGTCACCCAGAAGGACACAGGAATCTACACCGTAGTAGTCTACCTTCCAGGCTCAATAAAAGAAATAGGATTTGGACGGCTCAATGTGTACG

>Mlu_CEACAM1-like_5N pseudogene

TCTTCCTCTTAACCTTGTGGATCCCGCCCACCACTGTGCGATTTGCTATTGTCTCGATTAATGCTGCTGAAGGGCAGGATGTGATTCTTCGTACCCGCAATAGGCCTCCCACTTGTGCAGGCTTTATTTGGTACAGGGGAGAAAAGATGGACTACTATCATTTTATTGAGTCTGTTGCATGGGGTGTGAGACGATATAGACAGGGCCCGAATACAGTGGTCGAGAGACAGCAAATCTTGAAGGATCCCTGATCATAAGAAAAGTCACTCTGAAGGACACAGGAACCTACACCGTAATAGCCGTCCTTCAAAATTCACTAAGAGAAATAGGATTTGGACAGCTCAATGTATACCGGGA

>Mlu_CEACAM1-like_6N

TTTCACTCTTAAATTTCTGGAGCCTGCCCACCACTGCCCAACTTGCTATTGTGTCAACCAATGCTGCCGAAGGGAAGGATGTGATTCTACGTATCCACAATATGCCTCCTGATAATATAGGCTTCGTGTGGTACAGGGGGGAAGGAGCAAACTACAGACATACCATTGCCAGTCTTGCATTCCACATAAGAACTTATAGTACAGGGCGTGCATACAGTGGTCGAGAGCAAATAAATTTTGATGGATCCCTGCTGATAAAGAGGGTAACCCTGAAGGACACAGGAATCTACACCGTAGTAGTCTACCTTCGAGACTATAAAAAAGAAATAGGATTTGGACGGCTCAATGTATATG

*Macaca mulatta* (rhesus macaque)

>Mml_CEACAM1N

CCTCACTTCTAACCTTCTGGAACCCACCTACCACTGCCCAGCTCACTATTGAATCCAGGCCGTTCAATGTCGCAGAGGGGAAGGAGGTTCTTCTACTCGCCCACAATCTGTCCCAGAATCTTATTGGCTACAACTGGCACAAAGGGGAAAGAGTGGATGCCAAACGTCTAATTGTAGCATATGTAATAAGAACTAAACAAACTACCCCAGGGCCTGCACACAGCGGTCGAGAGACAATAGACTCCAATGCATCCCTGCTGATCCAGAACGTCACCCAGAATGACACAGGATCCTACACCCTACAAGTCATAAAGGGAGATCTTGTGAATGAAGAAGCAACTGGCCAGTTCCGGGTATATC

>Mml_CEACAM16N1

CCACATTCCTGAATGTGGGGGCTGAGATCTCTATCACCCTGGAGCCTGCCCAGCCGACCGAAGGGGACAACATCACGCTGGTCGTCCACGGGCTTTCGGGGGAACTGCTCGCCTACAACTGGTACGCGGGGCCCACGCTCAGCGTATCGTACCTGGTGGCCAGCTACATTGTGAGCACAGGCGATGAGACTCCTGGCCCGGCCCACACGGGGCGGGAGGCTGTGCGCCCCGATGGCAGCCTGGACATCCAGGGCGTCCTGCCCCGGCACTCAGGCACCTACATCCTGCAGACGTACAACAGGCAGTTTCAGACTGAGGTGGGCTACGGACACATGCAGGTCTATG

>Mml_CEACAM16N2

CTGCAGCAGTTGCCATGATGATCGTGCCCGTGCCCACCAAGCCAATGGAGGGCCAGGACGTGACGCTGACCGTGCAGGGCTACCCCAAGGACCTGCTGGTCTACGCCTGGTACCGCGGGCCTGCCTCCGAGCCCAACCGGCTGCTCAGCCAGCTGCCGTCAGGAACCTGGATTGCAGGCCCCGCACACACAGGCCGGGAGGTGGGCTTCCCCAACTGCTCACTGTTGGTGCAAAAGCTGAACCTCACAGACACTGGCCGCTACACACTCAAGACCGTCACACTGCAGGGCAAGACCGAGACACTGGAAGTGGAGCTGCAGGTGGCCC

>Mml_CEACAM18N

CCAGTCTGCTGGCCTGTGGGATCTGCCAGGCCTCTGGCCAAATCTTCATCACCCAAACCCTGGGGATCAAGGGATATCGGACTCTCCTGGCCCTGGATAACATCCCTGAGGATGTTCAGGAATACAACTGGTACCGGGGTGCAAACGACAGCGTGGGAAACATGATAATCAGCTGCAAACCGCCCAATGCTGAGCAGCCTGGGCCCATGTACACTGGCAGGGAAAGAGTGAACAGAGAAGGCAGCCTGTTGATCAGGCCCACTGCATTAAATGACACAGGAAACTACACTGTTCGGGTGGTTGCAGGCAATGAGACCCAAAGAGCAACTGGCTGGCTGGAGGTTCTAG

>Mml_CEACAM19N

CCTCAATCCTGGTTCTCTGGATGCTCCAAGGCTCCCAGGCAGCCCTCCACATCCAGAAGATTCCAGAACAGCCTCAAAAGAATCAGGACCTTCTCCTGTCAGTCCAGGGTGTCCCAGACACCTTCCAGGACTTCAACTGGTACCTGGGGGAGGAGATGTATGGAGGCACGAGGCTATTTACCTACATCCCTGGGATACAACGGCCCCAGAGGGATGGCAGTGCCATGGGACAGCGAGACATCGTGGGCTTTCCCAATGGTTCTATGCTGCTGCGCCGCGCCCAGCCTACAGACAGTGGCACCTACCAAGTAGCCGTTACCATCAACTCTGAATGGACTATGAAGGCCAAGACTGAGGTCCAGGTAGCTG

*Microcebus murinus* (gray mouse lemur)

>Mmr_CEACAM18N

CCAGTCTGCTGGCCTGTGGCATCTGCCAGGCCTCGGGCCAAATCTTCATCCCCCACGTCCAAGGGGTGCAGGGATATTCGAGTGTCCTGGGCCTAGAGAACGCCCCCGAGGATGCCCAGGAATACCGCTGGTACCGGGGCACACAGGACAGCGCAGAAAGCATGATTCTCAGCTACAAACCTCCCAGTCCCCTGGAGCCTGGGCCCGTGTACAGCGGCCGGGAGAGAGTGACCAGAACCGGCGACTTGGTGGTCAGGAACTGCATGCTAAACGACACAGGAAACTACACGGTCCGGGTGGACACAGGCAACGACACCCACACGGCAACTGGCTGGCTCGAGATCCGAG

>Mmr_CEACAM19N

CCTTGATCCTGGCACTCTGGGTGCCCCAAGGCTCCCAGGCAGCCCTGCGCATCCAGAAGATTCCAGAGCAGCCTCAGAAGAACCAGGACCTTCTCCTGTCTGTCCAGGGTGTCCCAGACACCTTCCAGGACATCAACTGGTACCTGGGGGAGGAGGCCTATGGAGGCACAAGGCTCTTCACCTACATACCGGAGCTCCAGCGGCCACAGAGGGACGGCAGTGCCATGAAGCAGCGGGACATCGTGGGCTTCCCCAATGGCTCCATGCTGCTGCGGCGTGCCCAGCCCACAGACAGCGGCACCTACCAAGTAGCCATCACCATCAACCCTGCCTGGACCATGAAGGCCAAGACCGAGGTCCAGGTGGCCG

*Ornithorhynchus anatinus* (platypus)

>Oan_CEACAM16N1

cctccgttctaactgCTTGGCTACCTCCTGCGCCCGCACAGCTGACCGTCACTCCCATCCCTCCCAACCCCCTCGAGGGCTGGGACGTGACGCTGTCTGTCAGCGGGGCCCCCGGCGGCCTGCTGCTCTACAACTGGTACAGGGGGGCGTCTCTCAGCCTGACTCAGATGATCCTCAGCTACATCAACGCCACTGAGATCCAGACTCCCGGGGCGGCCCACAGTGGCCGGGAGGCCGTTCACCCCAACGGCTCCCTCCTGATCCAGAGAGTAACCTTAAACGACTCCGGCAGCTACCTTCTACAATCCATCAACCCGCAGTTCCAGACAGAAATAGCGTTTGGCTTTTTGAGGGTTTATG

>Oan_CEACAM16N2

TCATGGCCTCAAGCTTCATGATTGTGCCCATTCCCGCACGCCAGGTGGAGGGGCAGGATGTGACCCTCTCCGTCCAGGGCTGCCCCAGGGACCTCCTGGTGTACGCCTGGTACCGAGGGACGCCGGATGAGCCCAATCGACTAGTCAGCCAGTTGCCGCTGGGGAACTGGATCGCAGGGCCTGCCCACAGCGGCCGGGAGACCGGCTTCCCCAACTGCTCCTTGCACATCCAGCACTTGAACGCCAGCGACTCAGGCCGCTACACCCTCAAGACCGTCACCCTGCAGGGCAAGACCGAGATGCTGGAGATCGAGCTGCGGGTCCTGG

>Oan_CEACAM16LN1

CTTTGGTCCTGAGCACCTGGCTACCCACAGCGTCCCTGCAATTGACCCTGGTGTCCATCCCACCGCAGCCTACTGAGGACCAGGTTGTCACTCTCTCTGTCCGGGGAGTTCCCAGGGACAGCAAGGAAGTTCTGCTGGTACCGGGnnnn

>Oan_CEACAM16LN2

nnnnCCCGGGCCCGCTCACACTGGCCGGGAGGTGGGGCTGGCCGATGGTTCCCTCCAGATCCAGGAAGTCAGGGAAGATGACGCTGGCCAGTACACCCTACAGGTCATCAGCCTTCTTGCCGGCAGCCCACCACTCACCGGGACTGTTAATTTAAAACTTTCTG

>Oan_CEACAM16LN3

GGCTCCGGATCGTGCCAGTTCCCCAGCATCCCAGGGTCGGGGAGGACATCACCCTGTCTATCTGGGGCCTGCCGGGGCTGCCCAAGACCTACACGTGGTACCGCGGGGCGGCGGGAAAACCCCATCTCATCCTCCGCTATAACACCGGTGTTGGTGTCTCCAACTGGCTTCTGGGCCCGGCCCACAGCGGGCGGGAGGCGGGCACCAAGAATGGCTCGTTGCTCCTCCGAGACGTCAGCCCCCAGGACTCCGGGCGCTACATCGTGCACGTGGTCACCTTGCAGGGGAAAGAGGAGGCCTCCATCACCATCGATGTCTCTCTGGGGCCCACGACCCCCCCAGCGGAGAAGGTGCCA

>Oan_CEACAM16LN4

GACTCCAGATTGTGCCAGTTCCCCAGCATCCCAGGGTCGGGGGGAACATCACCCTGTCTATCTGGGGCCTGCTGGAGCTGCCCAAGACCTACACGTGGTACCGCGGGGCGGCGGGAGGCCCCCATCGCATCCTCCGCTGTAACACCGGTGGTAGTGCCCCCAACTGCCTTCCGGGCCCGGCCCACAGCGGGCGGGAGACGTGCACCAAGAATGGCTCGTTGCTCCTCCGAGACGTCAGCCCCCAGGACTCCGGGCGCTACATCGTGCACGTGGTCACCTTGTGGGGGAAAGAGGAGGCCTCCATCACCATCGACGTCTCTCCGGAGCCAACGACCACCCCAGCGGAGAAGGCCCCAG

>Oan_CEACAM200N

CCTCCGTCCTCACGGCCGGGGTCCGGCCCGCCGTGTCTCTCACCGTCCGACCAGCCGTGGCTGTAGCCGGGGACAACGTCACTCTGACCGCCCAGGGATATCCAGACCAAGTTGGACTCTATAATAGATGGTACCGAGGGACAAGTCTGGAGGAAAACCTCATCTCTGAGCACCGATCGGCCTCAGACATTCAGACATCTGGAAACGCCTACACGGGCAGCGAGTCCGTGCGTCCAAATGGTTCTCTGCTCATCACCGAAGTTACCGTCAACGATACCGGACTCTACTTAATAAACGTCACATTCAATAATGTGACTTCTATTTCAGGGCGAGAGTTACGGGTTTATG

>Oan_CEACAM201N

CCTCCATCCTCACCGTCGGGGTCCGGCCCGCCGTGTCTCTCACCGTCCGACCAGCCGTGGCTGTAGCCGGTGACAACGTCACTCTGACCGCCCGGGGAAACCCAGACCAAGTTGGAATCTATAGATGGTACCGAGGGACAAGTGTGAAGGAAAACCTCATCTTTGAATACCAAACAGCCTCAGGCAATCAGATGTTTGGAGCCGCCTACACGGGCCGCGAGTCCACACGCTCCAATGGCTCCCTGCTCATCACCGACGTTACGGTCAATCATACCGGTTCCTACTATGTAGACATGGTACTCAAGAATTTTACTTCTTCTTCAGACGAAGGAGAGTTACAGGTTTACG

>Oan_CEACAM202N

CCTCCATCCTCACTGCCGGGGTCCGGCCCGCGGAGCCTCTCACCATCCGACCAGACGTGGCTGTAGCCGGGGACAACGTCACTCTGACCGCCCAGGGAAACCTAGCCCTCGTTGGACTCTGTAGATGGTACCGAGGGACAAGTCTGGAAGAAAGCCTCCTGTTTGCATACCGAACAACTTCAGGTCAGATACTTGGAAACGCCTACACAGACCGTGAGTTTTTGCTCCCAGATGGCTCCTTGCTCATCACCAACGTTACAGTCAAAGATGCCGGTCCCTACTTTGTAGACATCGTATTCAAGAATTTTACTTATTTGTCAAGCCACGGAGAGTTACAGGTTTATG

>Oan_CEACAM203N

CCTCCGTCCTGACCAACGGGGTCCGGCCCGCCGAGCCTCTCACCGTCCGACCAGCCGTGGCTGTAGCCGGGGACAACGTCACTCTGACCGCCCGGGGAGACCTAGACCAAGTTGGAATCTATAGATGGTACCGAGGGACAAATGTGAAGGAAAACCTCATCTGTGAATACCAAACAGTCTCAGGCAATCAGATGTTTGGAGCCGCCTACACGGGCCGCGAGTCCGCACGCTCCAATGGCTCCCTGCTCATCACCGACGTTACGGTCAATCATACCGGTTCCTACTATGTAGACATGGTACTCAAGGATTTTACTTCTTCTTCAGACGAAGGAGAGTTACAGGTTTACG

>Oan_CEACAM204N

nnnacccttGGAAAAGCACATACGGGCAGAGAGTCTGTGCAACCCAGTGGCTCCCTGCTCATCACCAGAGTGACAGTCAGCGATACCGGTCCCTACTCCGTAGACATCATATTTAAGAATTTTACTTTTACTTCAGACCGGGTGAAGTTACAAGTTTATG

*Oryctolagus cuniculus* (rabbit)

>Ocu_CEACAM16N2

CGGCAGCTGTCACCATGATGATCGTGCCTGTGCCCGCCAAGCCGTCGGAGGGCCAGGACGTGACGCTCACCGTGCAGGGCTACCCCAGGGACCTGCTGGTCTATGCCTGGTACCGCGGGCCTGCCTCCGAGCCCAACCGGCTGCTGAGCCAGCTGCCATCGGGGAACTGGATCGCGGGCCCCGCACACACGGGCCGCGAGGTGGGCTTCGCCAACTGCTCGCTGCTGGTGCAGAAGCTGAACGTCACGGACGCCGGCCGCTACACGCTCAAGACGGTGACGCTGCAGGGCAAGACCGACACCCTGGAGGTGGAGCTGCAGGTGGCCC

*Otolemur garnettii* (lemur)

>Oga_CEACAM16N2

CTGCAGCAGTTGCCATGATGATCGTGCCCATTCCCGCCAAACCGATGGAGGGCCAGGACGTGACACTAACTGTTCAGGGCTACCCCAAGGACCTGCTGGTCTACGCCTGGTACCGCGGGCCTGCCTCCGAGCCCAGTCGGCTGCTCAGCCAACTGCCATCGGGGAACTGGATCGCAGGCCCTGCGCACACAGGCCGGGAGGTGGGTTTCGCCAACTGCTCGCTGCTGGTGCAGAAACTGAATCTCACTGATGCCGGCCGATACACGCTCAAGACCGTCACACTGCAGGGCAAGACGGAGACTCTGGAAGTGGAGCTACAAGTGGCCC

>Oga_CEACAM18N

CCAGCCTGCTGGCCAGTGGGATCTGCCAGGCCGCTGGCCAAATCTTCATCACCCAACTCCTAGGGGTTGAAGGATATCGAAGTATCCTGGCCCTAGAGAACATCCCCGAGAACGTCCGGGAATACACCTGGTACCGAGGTACACAGGACAGTGCAGGAAATATGATTCTCAGCTACAAACTTCCCAACTCCTGGCAGCCTGGGCCCATGCACAGTGGCCGGGAGAATGTGACCAGAACAGGTGACTTGGTGATCAAGAAGTCTATGCTAAATGACACAGGAAACTACACTGTTTGGGTGGACACTGGCAATGAAACCCAAACAGCAACTGGCTGGCTTGAGATTGTTG

>Oga_CEACAM19N

CTTCAATCCTGGCACTCTGGATGCCCCAAGACTCCCAGGCAGCCCTCCATATCCAGAAGATTCCAGAGCAGCCTCAAAAGAACCAGGACCTTCTTTTGTCTGTTGACGGTGTCCCAGACACCTTCCAGGACGTTAACTGGTACCTGGGGGAGGAGACCTCTGGAGGTACAAGGCTATTCACCTATATACCTGGACTACAGCGGCCCCAGAGGGATGGCAGTGCCATGAAACACCGAGACATTGTGGGCTTCTCCAATGGCTCCATGCTGCTGTGACGTGCCCAGCCCACAGACAGTGGCACCTATCAAGTAGCCATCACCATCAACCCTGCCTGGACCATGAGGACTAAGACTGAGGTCCGGGTGGCAG

*Ochonto princeps* (American pika)

>Opr_CEACAM1N

TCTCACTTCTAACTTCTAACTGGATCCTGCCCACCATGGCTGAACTCAGAGTGGAACCAGTGCCATCCATTGCTGCCGAAGGAATGGATGTGTTTTTTCTTGTCTACGATCTACCAGATAATTATCCAGCCTTCATATGGTACAAGGGGGACCCAGAAGAGGAGAACTTTTATATTGCAACATATTTCAGAAGTGAAGGAGACATTACTGAAAAGGGACCTGCATATAGCAACAGACACACAATATTCCACAATGGGTCTCTGCTGATCCGTGGAGTCATCCCGGAGGACACTGGAAACTATACCCTAGATGCCATGGATTCACGGTTCAAGCATTACTATGCATATGGACATCTCAGCGTTTACT

>Opr_CEACAM1-like_1N CCTGCCTTCTAACTGTTGGGATTTCGCCCACCATGGCCAAATACACAGTGGAACCTGCACCGCCCATCGCTGCTGAAGGCACGGACACGCTTCTCCTCCTCCACAGCCCACCACAGAATGCTCAATTCTATAGCTGGTTCAAGGGAGAAATATCTAATGCAAGCCTTCGTATTGTAACATACACAGTCTTGGACAGCCAAGTTGATCCAGGGCCTGAACATACAGATAGGCACACAGTCTTCCCCAGTGGGTCCCTGCTGATCCAGGACACCATCCAGGAAGACACAGGGAGAATCCAAGATGCCTGAAGTTCCCCCACAGTCTTCTCACTTTCCCTCCCAGGGCTTGGACTCTTCCACTAGGCCAACCCCTCCA

*Procavia capensis* (hyrax)

>Pca_CEACAM16N1

TGGCCTTCCTGAGTGCGGAGGCAGAGATTTCCATCACCCCGGAGCCTGCCCAGCCGGCAGAAGGGGACAATTTCACACTGGTCGTCCAAGGTCTCTCCGGGGAGCTGCTGGCCTACAACTGGTACGCGGGGCCTACGCTCAGCCTGGCCCACCTGGTGGCTAGCTACATTGTGAGCACAGGCGACGAGACCCCCGGCCCAGCCCATACGGGGCGGGAGGCCGTGCGCCCTGACGCCAGCCTGGACATCCAAGGCGCCCTCCCCAGACACTCGGGGACCTACATCCTGCAGACTCTCAACAGGCAGTTTCAGACCGAGGTGGGCTACGGACACGTACAGGTCTATG

>Pca_CEACAM16N2

CGGCAGCGGTTGCCATGATGATCGTGCCAGTTCCAACCAGGCCATCAGAGGGCCAGGATGTGACCTTGACCGTGCAGGGCTACCCCAAGGACCTGCTGGTCTACGCCTGGTACCGAGGGCCTGCCTCCGAGCCCAACCGGCTACTCAGCCAGCTGCCGTCGGGGAACTGGATCGCGGGTCCCGCGCACACGGGCCGCGAGGTGGGCTTTCCCAACTGCTCACTGCTGGTGCAGAAGCTGAATCTCACCGACGCTGGCCGTTACACGCTCAAGACCGTCACCGTGCAGGGCAAGACCGAGACGCTGGAGGTGGAGCTGCAGGTGGCCC

*Pongo pygmaeus* (orangutan)

>Ppy_CEACAM1N

CCTCACTTCTAACCTTCTGGAACCCACCCACCACTGCCCAGCTCACTACTGAATCCACGCCATTCAATGTCGCAGAGGGGAAGGAGGTTCTTCTACTCGTCCACAATCTGCCCCAGAATCCTCTTGGCTACAACTGGTACAAAGGGGAAATGGTGGACGCCAACCATCGAATTATAGGATATGTAATATCAGATCAACTAACTACCCCAGGGCCTGCATACAGCAGTCGAGAGAAAATATACCCCAATGCATCCCTGCTGATCCAGAACGTCACCCAGAATGACACAGGATTCTACACCCTACAAGTCATAAAGTCAGATCTTGTGAATGAAGAAGCAACTGGACAGTTCCATGTATACC

>Ppy_CEACAM3N

CCTCACTTCTAAACTTCTGGAACCCGTCCACCACTGCCCAACTCACTATTGAATCCACGCCGTTCAATGTCGCAGAGGGGAAGGAGGTTCTTCTACTCACCCACAATCTGCCCCAGAATCATATTGGCTACACCTGGTACAAAGGGGAAAGAGTGGATAGCAACCGTCTAATTCTAGCATATAAAATAGAAACTCAAAAAGCTACCCCAGGGCCCGCATACAGTGGTCGAGAGACAATATGCCCCAATGCATCCCTGCTGATCCAGAACATCACCCAGAATGACACAGGATCCTACACCCTACAAGTCATAAGGTCAGATCTTGTGAATGAAGAAGCAACTGGACAGTTCCATGTATACC

>Ppy_CEACAM4N

CCTCACTTTTAACCTTCTGGGACCCGCCCAGCACTGTCCAGTTCACTATTGAAGCCCTGCCATCCAGTGCTGCAGAGGGAAAGGATGTTCTTCTACTGGCCTGCAATATTTTAGAGACTATTCAAGCCTATTATTGGCACAAGGGGAAAACGGCAGAAGGGAGCCCTCTCATTGCTGGTTATATAACAGACATTCAAGCAAATATCCCAGGGGCCGCATACAGTGGTCGAGAGACAGTATACCCCAATGGATCCCTGCTGTTCCAAAACATCACCCTGGAGGACGCAGGATCCTACACCCTACGAACCATAAATGCCAGTTACGACTCTGACCAAGCAACTGGCCAGCTCCACGTACACC

>Ppy_CEACAM5N

CCTCACTTCTAACCTTCTGGAACCCGCCCACCACTGCCCAGCTCACTACTGAATCCATGCCGTTCAGTGTTGCAGAGGGGAAGGAGGTTCTTCTACTCATCCACAATCTGCCCCAGAGTAGTATTGGCTACAACTGGTACAAAGGGGAAATGGTGGACGCCAACCATCGAATTATAGGATATGTAATATCAAATCAACTAACTACCCCAGGGCCTGCATACAGCAGTCGAGAGACAATATACCCCAATGCATCCCTGCTGATCCAGAACGTCATCCAGAATGACACAGGATTCTACACCCTACAAGTCATAAAGTCAGATCTTGTGAATGAAGAAGCAACTGGCCAGGTCCGGGTATACC

>Ppy_CEACAM6N

CCTCACTTCTAACCTTCTGGAACCCGCCCACCACTGCCCAACTCACTATTGAATCCACGCCGTTCAATGTCGCAGAGGGGAAGGAGGTACTTCTACTCGCCCACAATCTGTCCCAGAATCGTATTGGCTACATCTGGTACAAAGGGGAAAGAGTGGATGCCAACCGTCTAATTGTAGCATATAAAATAGAAACTCAACAAACTACCCCAGGGCCTGCATACAGTGGTCGAGAGACAATATACCCCAATGCATCCCTGCTGATCCAGAACGTCACCCAGAATGACACAGGATTCTACACCCTACAAGTCATAAAGTCAGATCTTGTGAATGAAGAAGCAACTGGACAGTTCCATGTATACC

>Ppy_CEACAM7N

CCTCACTTTTAACCTTCAGGAACCTGCCAACCAGTGCCCAGACCACTATTGAAGTCGTGCCGTTCAATGTCGCAGAAGGGAAGGAGGTCCTTCTACTAGTCCATAATGAGTCCCAGAATCTTTATGGCTACAACTGGTACAAAGGGGAAACGGTGCATGCCAACTATCGAATTATAGGATATGTACAAAATATGAGTCAAGAAAATGCCCCAGGGCCTGCACACAACGGTCGAGAGACAATATACCCCAATGGATCCCTCCTGATCCAGAACGTCACCCACAATGACGCAGGAATCTATACCCTACACGTTATAAAAGAAAATCTTAAGAGTGAAGAAGTAACCAGCCAATTCTACGTATTCT

>Ppy_CEACAM8N

CCTCACTTTTCACCTTCTGGAACCCGCCCACCACTGCCCAGCTCACTATTGAAGCTGTGCCATCCAATGCTGCAGAGGGGAAGGAGGTTCTTCTACTTGTCCACAATCTGCCCCAGGACCCTCTTGGCTACAACTGGTACAAAGGGGAAATGGTGGATGCCAACCGTCGAATTATAGGATATGTAACATCAAATCAACTAACTACCCCAGGGCCTGCATACAGCAGTCGAGAGACAATATACCCCAATGCATCCCTGCTGATGCGGAACGTCACCAGAAATGACACAGGATCCTACACCCTGCAAGTCATAAAGCTAAATCTTGTGAGTGAAGAAGTAACTGGCCATTTCAGCGTACATC

>Ppy_CEACAM16N1

CCACATTCCTGAATGTGGGGGCCGAGATCTCTATCACCCTGGAGCCTGCCCAGCCAACCCAAGGGGACAACGTTACGCTGGTCGTCCACGGGCTTTCAGGGGAACTGCTTGCCTACAACTGGTATGCGGGGCCCACACTCAGCGTGTCGTACCTGGTGGCCAGCTACATCGTGAGCACAGGCGATGAGACTCCTGGTCCGGCCTACACGGGGCGGGAGGCTGTGCGCCCCGATGGCAGCCTGGACATCCAGGGCATCCTGCCCCGGCACTCAGGCACCTACATCCTGCAGACCTTCAACAGGCAGTTGCAGACTGAGGTGGGCTACGGACACGTGCAGGTCCATG

>Ppy_CEACAM16N2

CTGCAGCAGTTGCCATGATGATCGTGCCCGTGCCCACCAAGCCAATGGAGGGCCAGGACGTGACACTGACCGTGCAGGGCTACCCCAAGGACCTGCTGGTCTACGCCTGGTACCGCGGGCCTGCCTCCGAGCCCAACCGGCTGCTCAGCCAGCTGCCGTCAGGAACCTGGATTGCAGGCCCCGCGCACACAGGCCGGGAGGTGGGCTTCCCCAACTGCTCGCTGTTGGTGCAGAAGCTGAACCTCACAGACACTGGCCGCTACACACTCAAGACCGTCACAGTGCAGGGCAAGACTGAGACACTGGAAGTGGAGCTGCAGGTGGCCC

>Ppy_CEACAM18N pseudogene

CCAGTCTGCTGGCCTGTGGGATCTGCCAGGCCTCTGGCCAAATCTTCATCACCCAAACCCTGGGGATCAAGGGATATCGGACTGTCCTGGCCCTGGATAACATCCCTGAGATGTTCAGGAATACAGCTGGTACCGGGGTGCAAACGACAGCGTGGGAAACATGATTATCAGCTACAAACCGCCCAATGCCCAGCAGCCTGGGCCCATGTACACTGGCAGGGAGAGAGTGAACAGAGAAGGCAGCCTGTTGATCAGGCCGACTGCATTAAATGACACGGGAAACTACACTGTTCGGGTGGTTGCAGGCAATGAGACCCAAAGAGCAACCGGCTGGCTGGAGGTTCTAG

>Ppy_CEACAM19N

CCTCAATCCTGGTCCTCTGGATGCTCCAAGGCTCCCAGGCAGCTCTCTATATCCAGAAGATTCCAGAGCAGCCTCAAAAGAACCAGGACCTTCTCCTGTCAGTCCAGGGTGTCCCAGACACCTTCCAGGACTTCAACTGGTACCTGGGGGAGGAGACGTATGGAGGCACGAGGCTATTTACCTACATCCCTGGGATACAACGGCCTCAGAGGGATGGCAGTGCCATGGGACAGCGAGACATCGTGGGCTTCCCCAATGGTTCCATGCTGCTGCGCCGCGCCCAGCCTGCAGACAGTGGCACCTACCAAGTAGCCATTACCATCAACTCTGAATGGACTATGAAGGCCAAGACTGAGGTCCAGGTAGCTG

>Ppy_CEACAM21N

CCTCACTTTTAACCTTCTGGAACGCGCCCACCACTGCCAGGCTCTTTATTGTATCAGTGCCCTTTGAAGTTGCTGAAGGGGAGAATGTTCATCTCTCTGTGGTTTATCTGCCCGAGAATCTTTACAGCTATGGCTGGTACAAAGGGAAAACGGTGGAGCCCAACCAGCTAATCGCAGCATATGTAATAGACACTCACGTTAGGACTCCAGGGCCTGCATACAGCGGTCGAGAGACAATATCACCCAGTGGAGATCTGCATTTCCAGAACGTCACCCTGGAGGACACGGGATACTACACCCTACAAGTCACATACAGAAATTCTCAGATTGACCAGGCATCTCACCATCTCCGTGTATACG

>Ppy_CEACAM22N

CCTCTGCAAGAAGCAGGTGTCTGCTCAACTGACGATCACATCCATCCCTCCCTGGGCCATCGAGGGGGGCAACGTCACCCTGTCTGTCCAGGCGATCCCTCAGAATTTCATCTCCTACAATTGGCTCCGAGGAGCAACCACCAATCAGGTTACCTGGAACCTCAATTTTAACTTTTTCAGCGGTGGCTACACCCCAGGACCAGCCCACACTGGCAGGGAAACAGGCAGTGCTGATGGTTCCCTGAACATTATTGATGTGTGTGCATCTGACCTGCGTCTCATCTCTTCTGGTGAAAACAGTTGCCATCATGCTATGCTACTTGTCTCTG

>Ppy_PSG1N Provisional assignment for PSGs

CATCACTTTTAAACTTCTGGAACACGCCTACCACTGCTCAAGTCACGATTGAAGCCCAGCCACCCAAAGTTTCTGAGGGGAAGGATGTTCTTCTACTTGTCCACAATTTGCCCAAGAATCTTACTGGCTACATCTGGTACAAAGGGCAAATGAGGGACCTCTACCATTACATTACATCATACGTAGTAGACGGTCAAACAATTATATATGGGCCTGCATACAGTGGACGAGAAACAGTATATTCCAATGCATCCCTGTTGATCCAGAATGTCACCCGGGACGACGCAGGATCCTACACCTTACACATCATAAAGCGAGGTGATGGGACTAGAGGAATAACTGGACATTTCACCTTCACCTTATACC

>Ppy_PSG2N

CATCACTTTTAAACTGCTGGAACCCGCCCACCACTGCCCAAGTCACGATTGAAGCCCAGCCACCCAAAGTTTCTGCGGGGAAGGATGTTCTTCTACTTGTCCACAATTTGCCCCAGAATCTTGCTGGCTACATCTGGTACAAAGGGCAAATAATGGACCTCTACCATTACATTACATCATACGTAGTAGACGGTCAAACAATTATATATGGGCCTGCATACAGTGGACGAGAAACAGTATATTCCAATGCATCCCTGCTGATCCAGAATGTCACCCGGGAGGACGCAGGATCCTACACCTTACACATCATAAAGCGAGGTGATAGGACTAGAGGAGTAACTGGATATTTCACCTTCACCTTATACC

>Ppy_PSG3N

CATCACTTTTAAACTTCTGGAACCCACCCACCACTGCCCAAGTCACGATTGAAGCCCAGCCACCCAAAGTTTCCGAGGGGAAGGATGTTCTTCTACTTGTCCACAATTTGCCCCAGAATCTTGCTGGCTACATCTGGTACAAAGGGCAAACGAGGGACCTCAACCATTACATTACATCATATGTTGCAGACAGTAAAATAATTATACATGGGCCTGCACACAGTGGACGAGAAACAGTATATTCCAATGCATCCCTGCTGATCCAGAATGTCACCCGGGAGGACGCAGGATCCTACACCTTACACATCATAAAGCGAGGTGATGGGATTAGAGGAATAACTGGACATTTCACCTTCACCTTATACC

>Ppy_PSG4N

CATCACTTTTAAACTTCTGGAACCCGCTTACCACTGCCCAAGTCATGATTGAAGCCCAGCCACCCAAAGTTTCTGAGGGGAAGGATGTTCTTCTACTTGTCCACAGTTTGCCCCAGAATCTTACTGGCTACATCTGGTACAAAGGGCAAATGAAGGACCTCTACCATTACATTACATCATACGAAGTAGACGGTCAAATAATTATACATGGGCCTGCATACAGTGGACGAGAAACAGTATATTCCAATGCATCCCTGCTGATCCAGAATGTCACCCGGGAGGACGCAGGATCCTACACCTTACACATCATAAAGCGAGGTGATGGGACTAGAAGAGTAACTGGAAATTTCACCTTCACCTTCTGTG

>Ppy_PSG5N

CATCACTTTTAAACTTCTGGAACCCGCCTGCCACTGCCCAAGTCATGATTGAAGCCCAGCCACCCAAAGTTTCTGAGGTGAAGGATGTTCTTCTACTTGTCCACAATTTGCCCCAGAATCTTGCTGGCTACATCTGGTACAAAAGGCAAATGATGGACCTCTACCATTACATTACATCATATGTAGTAGACGGTCAAATAATTATATATGGGCCTGCATACAGTGGACGAGAAACAGTATATTCCAATGCATCCCTGCTGATCCACAATGTCACCGGGGAGGATGCAGTATCCTACACCTTACACATCATAAAGCGAGGTGATGGGACTAGAAGAGTAACTGGAAATTTCACCTTCACCTTATACC

>Ppy_PSG6N

CATCACTTTTAAACTTCTGGAACCTGCCTACCACTGCCCAAGTCACGATTGAAGCCCAGCCACCCAAAGTTTTCGAGGTGAAGGATGTTCTTCTACTTGTCCACAATTTGCCCCAGAATCTTGCTGGCTACATCTGGTACAAAGGGCAAATGACGGACCTCTACCATTACATTACATCATATGTAGTAGATGGTGAAAGAATTATATATGGGCCTGCATACAGTGGACGAGAAACAGTATATTCCAATGCATCCCTGTTGATCCAGAATGTCACCCGGGAAGATGCAGGATCCTACACCTTACACATCATAAAGCGAGGTGATGGGACAAGAGGAGAAACTGGACATTTCAGCGTCAACTTATACC

>Ppy_PSG7N

CATCACTTTTAAACTTCTGGAACCTGCCAATCACTGCCTAAGTGACCATTGAAGCCCAGCCACCCAAAGTTTCTGAGGGGAAGGATGTTCTTCTACTTGTCCACAATTTGCCCCAGAATCTTGCTGGCTACATCTGGTACAAAGGGCAAATGACGGACCTCTACCATTACATAACATCATATGCAGTAGACAGTCAAATAAATATATATGGGCCTGCATACAGTGGACAAGAAACAGTGTATTCCAATGCATCCCTGCTGATCCAGAATGTCACCAGGGAGGACACAGGATACCATACCTTACACATGATAAAGCGAGGTGATAGGACTAGAGGAGTAACTGGACATTTCACCTTCACCTTATACc

>Ppy_PSG8N

CATCACTTTTAAACTTCTGGAATCCACCTACCACTGCCCAAGTCACGATTGAAGCCCAGCCACCCAAAGTTTCTGAGGGGAAGGATGTTCTTCTACTTGTCCACAATTTGCCCCAGAATCTTACTGGCTACATCTGGTACAAACGAAAAATGACGGACCTCTACCATTACATTACATCATATGTAGTAGATGGTCAAATAATTATATATGGGCCTGCACACAGTGGACGAGAAACAGTATATTCCAATGCATCCCTGCTGATCCAGAATGTCA

>Ppy_PSG9N

TTCTTCTACTTGGTCCACAATTTGCCCCAGAATCTGCTGGCTACGTTTGGTACAAAGGGCAAATGACATACCTCTACCATTACATTACATCATATGTAGTAGACGGTCAAAGAATTATATATGGGCCTGCATACAGTGGACGAGAAACAGTATATTCCAATGCATCCCTGCTGATCCAGAATGTCACCTGGGAGGATGCAGGATCCTACACCTTACACATCATAAAGCGAGGTGATGGGACTGCAGGAGTAACTGGACATTTCACCTTCACCTTATACC

*Pan troglodytes* (chimpanzee)

>Ptr_CEACAM1N

CCTCACTTCTAACCTTCTGGAACCCACCCACCACTGCCCAGCTCACTACTGAATCCATGCCATTCAATGTTGCAGAGGGGAAGGAGGTTCTTCTACTTGTCTACAATCTGCCCCAGCAACTTTTTGGCTACAGCTGGTACAAAGGGGAAAGAGTGGATGGCAACCGTCAAATTGTAGGATATGTAATAGGAACTCAACAAGCTACCCCAGGGCCCGCATACAGCGGTCGAGAGACAACATACCCCAATGCATCCCTGCTGATCCAGAACGTCACCCAGAATGACACAGGATTCTACACCCTACAAGTCATAAAGTCAGATCTTGTGAATGAAGAAGCAACTGGACAGTTCCATGTATACC

>Ptr_PSG1N

CATCACTTTTAAACTTCTGGAACCCGCCCACCACAGCCCAAGTCACGATTGAAGCCCAGCCACCCAAAGTTTCCAAGGGGAAGGATGTTCTTCTACTTGTCCACAATTTGCCCCAGAATCTTACTGGCTACATCTGGTACAAAGGGCAAATGAGGGACCTCTACCATTACATTACATCATATGTAGTAGACGGTCAAATAATTATATATGGGCCTGCATATAGTGGACGAGAAACAGTATATTCCAATGCATCCCTGCTGATCCAGAATGTCACCTGGGAGGACGCAGGATCCTACACCTTACACATCATAAAGGGAGGTGATGAGACTAGAGGAGTAACTGGACGTTTCACCTTCACCTTATACC

>Ptr_PSG2N

CATCACTTTTAAACTTCTGGAACTCGCCCACCACTGCCCAAGTTACGATTGAAGCCCAGCCACCAAAAGTTTCCGAGGGGAAGGATGTTCTTCTACTTGTCCACAATTTGCCCCAGAATCTTACTGGCTACATCTGGTACAAAGGGCAAATAAGGGACCTCTACCATTACATTACATCATATGTAGTAGACGGTCAAATAATTATATATGGGCCTGCATATAGTGGACGAGAAACAGTATATTCCAATGCATCCCTGCTGATCCAGAATGTCACCCGGGAGGACGCAGGATCCTACACCTTACACATCACAAAGCGAGGTGATGGGACTAGAGGAATAACTGGAAATTTCACCTTCACCTTATACC

>Ptr_PSG3N

CATCACTTTTAAACTTCTGGAACCCGCCTACCACTGCCCAAGTCACGATTGAAGCCCAGCCAACCAAAGTTTCCAAGGGGAAGGACGTTCTTCTACTTGTCCACAATTTGCCCCAGAATCTTGCTGGCTACATCTGGTACAAAGGGCAAATGACGGACCTCTACCATTACATTACATCATACGTAGTAGATGGTCAAATAATTATATATGGGCCTGCATACAGTGGACGAGAAACAGTATATTCCAATGCATCCCTGCTGATCCAGAATGTCACCCGGGAGGACGCAGGATCCTACACCTTACACATCGTAAAGCGAGGTGATGGGACTAGAGGAATAACTGGACATTTCACCTTCACCTTATACC

>Ptr_PSG4aN

CATCACTTTTAAACTTCTGGAACCTGCCCACAACTGCCCAAGTCACGATTGAAGCCCTGCCACCCAAAGTTTCTGAGGGGAAGGATGTTCTTCTACTTGTCCACAATTTGCCCCAGAATCTTGCTGGCTACATTTGGTACAAAGGGCAAATGACATACCTCTACCATTACATTACATCATATGTAGTAGACAGTCAAAGAATTATATATGGGCCTGCATACAGTGGAAGAGAAACAGTATATTCCAACGCATCCCTGCTGATCCAGAATGTCACCTGGGAGGACGCAGGATCCTACACCTTACACATCATAAAACGAGGTGATGGGACTGGAGGAGTAACTGGACATTTCACCTTCACCTTATACC

>Ptr_PSG4bN

CATCACTTTTAAACTTCTGGAACCTGCCCACAACTGCCCAAGTCACGATTGAAGCCCTGCCACCCAAAGTTTCTGAGGGGAAGGATGTTCTTCTACTTGTCCACAATTTGCCCCAGAATCTTGCTGGCTACATTTGGTACAAGGGGCAAATGACATACCTCTACCATTACATTACATCATATGTAGTAGACAGTCAAAGAATTATATATGGGCCTGCATACAGTGGAAGAGAAACAGTATATTCCAACGCATCCCTGCTGATCCAGAATGTCACCTGGGAGGACGCAGGATCCTACACCTTACACATCATAAAACGAGGTGATGGGACTGGAGGAGTAACTGGACATTTCACCTTCACCTTATACC

>Ptr_PSG5N

CATCACTTTTAAACTTCTGGAACCTGCCTACCACTGCCCAAGTCACGATTGAAGCCCTGCCACCCAAAGTGTCTGAGGGGAAGGATGTTCTTCTACTTGTCCACAATTTGCCCCAGAATCTAGCTGGCTACATCTGGTACAAAGGACAACTGATGGACCTCTACCATTACATTACATCATATGTAGTAGACGGTCAAATAAATATATATGGGCCTGCATACACTGGACGAGAAACAGTATATTCCAATGCATCCCTGCTGATCCAGAATGTCACCCGGGAGGATGCAGGATCCTACACCTTACACATCATAAAGCGAGGTGATAGGACTAGAGGAGTAACTGGATATTTCACCTTCAACTTATACC

>Pt_PSG6N partial

ATTGAAGCCCAGCCACCCAAAGTTTCCGAGGGTAAGGATGTTCTTCTACTTGTCCACAATTTGCCCCAGAATCTTACTGGCTACATCTGGTACAAAGGGCAAATGAGGGACCTCTACCATTACATTACATCATATGTAGTAGACGGTCAAATTATATATGGGCCTGCCTACAGTGGACGAGAAACAGTATATTCCAATGCATCCCTGCTGATCCAGAATGTCACCCAGGAGGATGCAGGATCCTACACCTTACACATCATAAAGCGAGGCGATGGGACTGGAGGAGTAACTGGATATTTCACCGTCACCTTATACT

>Ptr_PSG7N not found

>Ptr_PSG8aN

CATCACTTTTAAACTTCTGGAACCTGCCCACCACTGCCCAAGTCACGATTGAAGCCCAGCCAACCAAAGTTTCCGAGGGGAAGGATGTTCTTCTACTTATCCACAATTTGCCCCAGAATCTTACCGGCTACATCTGGTACAAAGGGCAAATGAGGGACCTCTACCATTACATTACATCATATGTAGTAGACAGTCAAAGAATTATATATGGGCCTGCATACAGTGGACGAGAAACAATATATTCCAATGCATCCCTGCTGATCCAGAATGTCACCCGGGAAGACGCAGGATCCTACACCTTACACATCATAAAGGGAGGTGATGAGACTAGAGGAGTAACTGGACATTTCACCTTCACCTTATACC

>Ptr_PSG8bN

CATCACTTTTAAACTTCTGGAACCTGCCCACCACTGCCCAAGTCACGATTGAAGCCCAGCCAACCAAAGTTTCCGAGGGGAAGGATGTTCTTCTACTTATCCACAATTTGCCCCAGAATCTTACTGGCTACATCTGGTACAAAGGGCAAATGAGGGACCTCTACCACTACATTACATCATATGTAGTAGACAGTCAAAGAATTATATATGGGCCTGCATACAGTGGACGAGAAACAATATATTCCAATGCATCCCTGCTGATCCAGAATGTCACCCGGGAAGACGCAGGATCCTACACCTTACACATCATAAAGGGAGGTGATGAGACTAGAGGAGTAACTGGACATTTCACCTTCACCTTATAC

>Ptr_PSG9N

CATCACTTTTAAACTTCTGGAACCCGCCCACCACTGCCGAAGTCACGATTGAAGCCCAGCCACCCAAAGTTTCTGAGGGGAAGGATGTTCTTCTACTTGTCCACAATTTGCCCCAGAATCTTCCTGGCTACTTCTGGTACAAAGGGGAAATTACGGACCTCTACCATTACATTATATCATATATAGTAGATGGTAAAATAATTATATATGGGCCTGCATACAGTGGAAGAGAAACAGTATATTCCAACGCATCCCTGCTGATCCAGAATGTCACCCGGGAGGATGCAGGAACCTACACCTTACACATCATAAAGCGAAGTGATGAGACTAGAGAAGAAATTCGATATTTCTCCTTCACCTTATACT

>Ptr_PSG11N

CATTACTTTTAAACTTCTGGAACCTGCCTACCACTGCCCAAGTCATGATTGAAGCCCAGCCACCCAAAGTGTCTGAGGGGAAGGATGTTCTTCTACTTGTCCACAATTTGCCCCAGAATCTTACTGGCTACATCTGGTACAAAGGGCAAATCAGGGACCTCTACCATTACATTACATCATATGTAGTAGACGGTCAAATAATTATATATGGACCGGCATACAGTGGACGAGAAACAGTATATTCCAATGCATCCCTGCGGATCCAGAATGTCACCCGGGAGGACGCAGGATCCTACACCTTACACATCATAAAGCGAGGTGATGGGACTAGAGGAATAACTGGAAATTTCACCTTCACCTTATACC

>Ptr_CEACAM16N1

CCACATTCCTGAATGTGGGGGCCGAGATCTCTATCACCCTGGAGCCTGCCCAGCCGAGCGAAGGGGACAACGTCACGCTGGTCGTCCATGGGCTTTCGGGGGAACTGCTCGCCTACAGCTGGTATGCGGGGCCCACACTCAGCGTGTCATACCTGGTGGCCAGCTACATCGTGAGCACAGGCGATGAGACTCCTGGCCCGGCCCACACGGGGCGGGAGGCTGTGCGCCCCGATGGCAGCCTGGACATCCAGGGCATCCTGCCCCGGCACTCAGGCACCTACATCCTGCAGACCTTCAACAGGCAGTTGCAGACCGAGGTGGGCTACGGACACGTGCAGGTCCATT

>Ptr_CEACAM18N GGGATCTGCCAGGCCTCTGGCCAAATCTTCATCACCCAAACCCTGGGGATCAAGGGATATCGGACTGTCCTGGCCCTGGATAACGTCCCTGGGGATGTTCAGGAATACAGCTGGTACTGGGGTGCAAACGACAGCGCAGGAAACATGATTATCAGCCACAAACCGCCCAGTGCCCAGCAGCCTGGGCCCATGTACACTGGCAGGGAGAGAGTGAACAGAGAAGGCAGCCTGTTGATCAGGCCGACTGCATTAAATGACACGGGAAACTACACTGTTCGGGTGGTTGCAGGCAATGAGACCCAAAGAGCAACCGGCTGGCTGGAGGTTCTAG

>Ptr_CEACAM19N

CCTCAATCCTGGTCCTCTGGATGCTCCAAGGCTCCCAGGCAGCTCTCTACATCCAGAAGATTCCAGAGCAGCCTCAAAAGAACCAGGACCTTCTCCTGTCAGTCCAGGGTGTCCCAGACACCTTCCAGGACTTCAACTGGTACCTGGGGGAGGAGACGTATGGAGGCACGAGGCTATTTACCTACATCCCTGGGATACAACGGCCTCAGAGGGATGGCAGTGCCATGGGACAGCGAGACATCGTGGGCTTCCCCAATGGTTCCATGCTGCTGCGCCGCGCCCAGCCTACAGACAGTGGCACCTACCAAGTAGCCATTACCATCAACTCTGAATGGACTATGAAGGCCAAGACTGAGGTCCAGGTAGCTG

*Sorex araneus* (European shrew)

>Sar_CEACAM1-like_1N

CCACACTCTTACCTTTCTGGAGCCTGCCCGCCAATGCTGCATCTGTGGAAATAATACCAGCCAATGCTGTGGTAGGGGACAATGTCACCATGTTTGTCCACAAGGTGCCAGAAAATATTGTACGCTTAGACTGGTGCAAAGGGGTAATTGAATCAAAGAACATCATTATAACATTGAAAAAAAACCCAGAAAATATTACTAATGGGCCTTTATTCAGCCACCGAGAGACCCTGCTCAACAACGGAACCCTGCACATCCAGAACCTCACCGAGAGGGACAAGAACACCTACCACGTATACGTCTTTACAATGGGAGGTGACTCTATAGCAGCAAAAGGATACCTCGGCGTGTACG

>Sar_CEACAM1-like_2N

CCGCACTCTTACCTTTCTGGAGCCTGCCCGCCAATGCTGTATCTGTGGAAATAATACCAGCCAATGCTGCGGTAGGGGACAATGTCACCATGTTTGTCCACGAGGTGCCAGAAAATATTGTACGCTTAGACTGGTGCAAAGGGGTAATTGAATCAAAGAACATCATTATAACATTGAAAAAAAACCCAGAAAATATTACTAATGGGCCTTTATTCAGCCACCGAGAGACCCTGCTCAACAACGGAACCCTGCACATCCAGAACCTCACCGAGAGGGACAAGAACACCTACCACGTATACGTCTTTACAATGGGAGGTGACTCTATAGCAGCAAAAGGATACCTCGGCGTGTACG

>Sar_CEACAM11-like_3N

CCACACTCTTACCTTCCTGGAGCCTGCCCGCCAATGCTTTTTCTGTGAAAATAATACCAGCCAATGCTGTGGTAGGCGACAATGTCACCATGCTTGTCCACAAGGTGCCAGAAAATGTTTTTCGCTTCGACTGGTACAAAAAGTCAATTAAACCGAGGAATATCATTGTTACATTGAAAACAAACCCAGAAAATATTACTAATGGGCCTTTATTCAGCAACCGAGAGACCCTGTTCAGCAACGGAACCCTGCACATCCGGAACCTCACCCAGAGGGACAAGGCCACCTACATCGTACAAGCCATTACAATGGGAAGTGACGTTTTAACAGCAAAAGGACAGCTCGGCGTGTATG

>Sar_CEACAM16N1

CCGCCTTCCTGGGTGCGGGGGCGGAGATTCTCATCACGCCTGAGCCGGCCCAGCCCGCGGAGGGCGACAACGTGACGCTGGCGGTGCAGGGCCTGGCCGGGGAGCTGCTGGCCTACAGCTGGTACGCGGGGCCCTCCCTCAGCCTGGCCTACCTGGTGGCCAGCTACATCGTCAGCACCGGCGACGAGACGCCCGGGCCCGCCCACACGGGCCGAGAGGTGGTCCGTGCCGACGGCAGCCTGGACGTCCACGGGGTCCTGCCCGGGCACTCGGGCACCTACATCCTGCAGACGCTCAACAGGCAGCTCCAGACGGAGGTGGGCTACGGACACTTGCAGGTCTATG

*Sus scrofa* (pig)

>Ssc_CEACAM1N

GCTCACTCCTAACCTTCTGGAACCTGCCCACCACTGCCCAGATCACTATTGAATCAGTGCCCTTCAATGCTGTGGAAGGGACAAGTGTTCTTCTACTTGTCCACAATGTGACAGAGAATATCCTAGACTACTGCTGGTACAAAGGAGAAAGGGTAGAGACCAACCAGTTAATCGCATCATGTAGAGTGGACGCTCAAGCAAATACCCCAGGGCCTGCACAGAGCAGTCAAGAGACAATCTACCCCAATGGATCCCTGCTGTTCCAGAAGGTCGCCCAGAGTGACACAGGAAACTACTACACCCTACTTGCCACAAAGAGAGATAATCAGACTGAAAGTGTGACTGGACAACTCCGTGTGTACC

>Ssc_CEACAM1-like_1N

tctcactcctaagCTTCTGGAACTTGCCCGCCACTGCCCAGATCACTATTGAATCAGTGCCCTTCAATGTCGCAGAAGGAAGCGATGTTCTTCTACTTGCCCACAATGCGACAGAGGATACTCTAGGCTACAGCTGGTACAGAGGAGAAAGAGTAGAGAACAACCAACTAATTGTAACATACAGAATAGACACTCAAGCATATACCCAAGGACCTACATTCAGTGGTCGAGAGAGAATCTACCCCAATGGATCCCTGCTGTTCCAGAATGCCACCCAGAATGACACAGGATACTACACTCTGATGGTTACAAAGAATGATTTACAGAGAGAAATTGTAACTGGACTACTCCGTGTATACC

>Ssc_CEACAM1-like_2N

CTCACTCCTAAGCTTCTGGAACCTGCCCGCCACTGCCCAGATCACTATTGAATCAGTGCCCTTCAAAACTGCAGAAGGAAGGGATGTTCTTCTACTTGCCCACAATGCGACAGAGGATACTCTAGGCTACAGCTGGTACAGAGGAGAAAGAGTAGAGAACAACCAACTAATTATATTATATAGAGTAGACATTCAAGCAAATACCACAGGGCCTGCATACAGTGGTCGAGAGATAATCTACCCCAATGGATCCCTGCTGTTCCAGAATGCCACCCAGAATGACACAGGATACTACACTCTGATGGTTACAAAGAATGATTTACAGAGAGAAATTGTAACTGGACTACTCCGTGTATACC

>Ssc_CEACAM16N2

CGGCAGCTGTCGCCGTAACAATTGTGCCCGTGCCAACCAGGCCGATGGAGGGCCAGGACGTGACGCTGACCGTACAGGGCTACCCCAAGGACCTGCTGGTCTACGCCTGGTACCGGGGGCCTGCTGCGGAGCCCAACCGACTGCTTAGCCAGCTGCCTTCGGGGAACTGGATCGCAGGCCCTGCGCACACAGGCCGGGAGGTGGGCTTCCCCAACTGCTCGCTGCTGGTACAGAAGCTGAACCTCACGGACGCAGGCCGCTACACGCTCAAGACCGTCACGCTGCAGGGCAAGACTGAGACGCTGGAAGTGGAGCTGCAGGTGGCCC

*Spermophilus tridecemlineatus* (ground squirrel)

>Str_CEACAM1N

TCTCACTGTTAACCTTCTGGAACCCGCACCCCACTGCCCAGCTCACTATTGAACCAGTGCCTTCCGACGTTGCTGAAGGGAGAGACGTTCTTCTACTTGTCCACAATGTGTCGGGGAACGCTGTAGGCTACTCCTGGTACAGAGGGCAAATAACAGACCGCAGCCGTCTAATTGTATCCTACTCGAATCTCACACACTCAGCTACCCCAGGCCTTGCCTTCAGTGGTCGAGAGACAGTATACCCCAATGGGTCCCTGCTGTTTGTGAATCTGAAAAAGGAGGACACAGGATTCTACACCCTTCAAACCACAACTGACAATTTTGATGCTGAAGTTGCAACTGGAGAGTTCCGTGTACATG

>Str_CEACAM1-like_1N

TCTCACTGTTAATCTTCTGGAACCCGCACCCCACTGCCCAGCTCACTATTGAACCAGTGCCTTTTGATGCTGCTGAAGGGACAGATGTTCTTCTACTTGTCCATGATGTGTCGGAGAATGCTGTAGGCTACACCTGGTACAGAGGGCAAATAACAGGCCGCAGCCATCTGATTGtatcCTACTCGAATATCACACACTCAGctATCCCAGGGCCTGCCTTCAGTGGTCGAGAGACAATATACCCCAATGGATCACTGCTGTTCATGAATGTGACAAAGGAGGACACAGGATTCTACATCCTACGAACCACAGCTGACAATTTTGATACTGAAGCAGCAATTGGAGAGTTCCGTGTACACA

>Str_CEACAM1-like_2N pseudogene

TCTCACTGTTAACCTTCTGGAACCCGCACCCCACTGCCCAGCTCACTATTGAACCAGTGCCTTTCGATGCTGCTGAAGGGAGAGACGTTCTTCTCCTTGTCCACAATGTGTCGGGGAATCTTGTAGGCTACTCCTGGTACAGAGGGCAAATAACAAACCAAAACCTTCTTATTGTATCCTACTCAAATTTCACACACTCAACTACCCAGGGCCTGCATTCAGTGGGCGAGAGACAATACACCGCAATGGGTCCCTGCTGTTCGTGAATGTGACAAAGGAGGACACAGGATTCTACACCCTTCAAACCATAGCTACTAATTTTCGTACTGAAGCAGCAACTGGAGAGTTCTGTGTACATG

>Str_CEACAM1-like_3N

TCTCACCTTTAACCTTCTGGAACCTGCCCACCACTGCCCAACTTGCTATTGAATCTGTGCCCTTCACTGCTGCCGAAGGGACCGATGTTCTTCTACTTGCCCACAACGTGTCAGAGAACGTCAGAGGCTACACCTGGTACAGAGGGGAAAGGGGCATTGACAGCCATCAAATCGCCATATACCTAACAGCCACTCGAGAAACCATCCCAGGGCCTGCCTACAGTGGTCGAGAGACGGTATACCCCAATGGATCCCTGCTGTTCCAGAAGGTCACCTTGGAGGACACGGGATTCTACACTCTGCAAACCCTAAGTAGCGATCTTCAGGCTGAAAAAGCATCTGGACACATCTGTGTATAT

*Tupaia belangeri* (tree shrew)

>Tbe_CEACAM1-like_1N

TCTCACTTTTAACCTTCTGGCACCTGCCCACCACTGCCCAACTCACTATCAAATCAGAGCCGTTCAATGCCACCCAAGGGAAGGATGTTCTACTAAGTGTTTACAATGTGCCAGTGGATGTTAGCGGGTACCAATGGTACAGAGGGGAAAGGGTGGGCGGGAGCCAACTAATTTTACAATATGAGACGAATTCTCAAAGAGTCACTGAAGGACCTCAATATAGCGGTCGAAAGGAAATATTCTCCAATGGATCCCTGCTCCTCCGTAATGTCAGCCAAGAAGACGCGGGATTCTACACCGTAGTTACCATAACTGGCATTGAAACTGACCAAGCAACTGGTCAATTCCACGTATTCT

>Tbe_CEACAM1-like_2N

TCTCACTTTTAACCTTCTGGCACCTGCCCACCACTGCCCAACTCACTATCAAATCAGAGCCGTTCAATGCCACCCAAGGGAAGGATGTTCTACTAAGTGTTTACAATGTGCCAGTGGATGTTAGCGGGTACCAATGGTACAGAGGGGAAAGGGTGGGCGGGAGCCAACTAATTTTACAATATGAGACGAATTCTCAAAGAGTCACTGAAGGACCTCAATATAGCGGTCGAAAGGAAATATTCTCCAATGGATCCCTGCTCCTCCGTAATGTCAGCCAAGAAGACGCGGGATTCTACACCGTAGTTACCATAACTGGCATTGAAACTGACCAAGCAACTGGTCAATTCCACGTATTCT

>Tbe_CEACAM1-like_3N pseudogene

TGTTAGCCTTCCAGAACCTGCCAGTCACTGACCCGCTCACTACTGAATCAGTGCCGTTCACTATTGAAGGAAAGGTTGTTCTGCTTGCCCGTAACGTGCCAAAGGATATTCAAGTCTCCATCTGGTACAAAGGACAAAGGGTGGATGCCAACCGTGAAATTGCAGCATACATAtaaGGTACTACCCCAAGACCTGCACACAGTGGCtgaGAAACAATATTCCCCAGTGGATCTCTGCTGTTCCAAAATGTCACCCAGGAGAACTCAGGGTACTACACCTTACAAACCATAAATGAACATTTTCAGACTGACCAAGGATCTAGACAGTCTCACGTTTTC

>Tbe_CEACAM1-like_4N

CCTCCTTCCTGAGCTTCTGCAGCAAGCCCATGTCTGCCCAGCTGACCATCACGTCCATCCCTCCCCGAGCTATCGAGGGGGACAACGTCACCCTGTCTGTCCAGGGGATCCCTCAGAATCTCCTCTCCTACAACTGGTTCCGAGGAGCAACCATTGATCAGGTTAACCGGATTGTGAATTTCAAATTCACCGACCATGCCCACACTCCAGGACCAGCTCACACTGGCAGGGAAACGGGCAGTGCCAGTGGCTCCCTGAGCATCACCGATGTGCGGAGGTCGGATGACGGCATCTACACCCTGCAGCTCAT

>Tbe_CEACAM1-like_5N pseudogene

CAGGTCGTTCACAATTCCAGGAGAGGTGGATGTCTGAAAGTGGGGACCATGAACTTCCTGGACCTTTGTGACCTTCGTGGACAAAGTCCACAAGATCCATTGGAGAATATTGTCTCTCGACCGCTCTATGGAGGTCCTTCAGTGATTCTTTGAGAATCCGCCTCATACAGTAAAATTCGTTGGCTCCTGTCCACTTTCCCCCCTCTGCACC

>Tbe_CEACAM16N2

CGGCGGCAGTCGCTATGACCATCGTGCCGGTGCCCAGCAAGCCCACGGAGGGCCAGGACGTGACGCTGACCGTACAGGGCTACCCCAAGGACCTGCTGGTCTACGCCTGGTACCGCGGGCCCGCCTCCGAGCCCAGCCGGCTGCTCAGCCAGCTGCCGTCCGGGAACTGGATCGCAGGCCCGGCGCACACGGGCCGCGAGGTGGGCTTCGCCAACTGCTCGCTGCTGGTGCAGAAGCTGAACCTCACGGACGCCGGCCGCTACACGCTCAAGACCGTCACCCTGCAGGGCAAGACCGACACGCTGGAGGTGGAGCTGCAGGTGGCCC

>Tbe_CEACAM18N

CCAATCTGCTGACCTGTAGGATGTTCCAGGCCTCTGGCCACATCTTCATCAGCCCCGACACCCTCGTAGGGGCTGTGGGATATCGGAGCATACTGATCCTTGAGAACGTCTCTGAGAATGTTCTGGAGTACAGCTGGCACCGGGGTGAAGAAGGCAACGCGGCCAACATGATTTTCAGCTACAGACCTCCCAGTTCCCGGCAGCCTGGGCCCATGTACAGCGGCCGAGAGGGCGTGACCCGCAACGGCGACCTGGTGATCAGGCCATCTGCACTGAATGACACGGGGAACTACACTGTCCAGGTGGACCTCGGCAACGAGACCCAGACAGCCACCGGCTGGCTCCAGATTATAG

>Tbe_CEACAM19N incomplete

CCTCAGTCCTGGTTCTCTGGATGCCTCAAAGCTCCTGGGCAGCCCTTTATATCCAGAAGATTCCAGAGCAGCCTCAAAAGAACCAGGATCTTCTCCTGTCTGTCCTGGGAGTCCCAGACACCTTTCAGGACTTCAACTGGTACCTGGGGGAGGAGACCAGTGGGGGCACAATGCTATTCTCCTACTTCCCCGAGCAACTGCGGCCCCAGAGAGACGGCAGTGCCATGGGAAACCGAGACAGCACCTACCACGTAGCAGTCACCATCAACCCTGCCTGGTCACCGGCTTCCCCAACGGCTCCATGCTGCTGCGTCGCGCCCAGCCTACAGACAGC
